# Review: Mean-Square Displacements of Simulated Polymers

**DOI:** 10.3390/polym17091193

**Published:** 2025-04-27

**Authors:** George D. J. Phillies

**Affiliations:** Department of Physics, Worcester Polytechnic Institute, Worcester, MA 01690, USA; phillies@4liberty.net; Tel.: +1-508-754-1859

**Keywords:** polymer dynamics, mean-square displacement, polymer melt, polymer solution, computer simulation, scaling behavior, scaling exponents, power-law behavior

## Abstract

We review simulations of polymeric fluids that report mean-square displacements g(t) of polymer beads, segments, and chains. By means of careful numerical analysis, but contrary to some models of polymer dynamics, we show that hypothesized power-law regimes $g\left(t\right)∼t^{\alpha }$ are almost never present. In most but not quite all cases, plots of log(g(t)) against log(t) show smooth curves whose slopes vary continuously with time. We infer that models that predict power-law regimes for g(t) are invalid for melts of linear polymers.

## 1. Introduction

In this paper, we consider a few aspects of polymer motion in simulated bead–bond polymer melts. Our interest is the time-dependent mean-square displacement of individual beads, of polymer centers of mass, and of polymer beads with respect to the center of mass of their polymer chain. We advance via a review of representative papers from the literature, applying a quantitative approach based on standard numerical methods. A short letter demonstrating the approach has been published previously [1].

The behaviors of the mean-square displacements are predicted by tube-reptation-scaling models of polymer dynamics [2,3]. For long polymers in a melt, the tube model proposes that the polymer chains surrounding a chain of interest form a transient pseudolattice that restricts the chain of interest’s lateral motions. The transient pseudolattice is described as a *tube*, an irregular curving barrier having statistical diameter *a* and contour length *L*, within which the chain of interest is momentarily confined. According to the tube model, the polymer chain of interest is said to be constrained to move more or less parallel to its tube.

For long polymer molecules, the tube model introduces three characteristic times. The hypothesized times are claimed to separate four characteristic time scales for polymer motion. At times shorter than an entanglement time $\tau_{e}$, beads are said not to have encountered the walls of the tube, so bead motions are claimed to be described by the simple Rouse model. At times between $\tau_{e}$ and a later time $\tau_{R}$, Rouse relaxations continue but are perturbed by the tube walls until they completely relax at a time $\tau_{R}$. $\tau_{R}$, the *Rouse time*, is the time scale on which the polymer’s longest-lived Rouse mode would relax, if the polymer’s dynamics were described by the Rouse model for a bead–spring chain [4]. $\tau_{R}$ is defined in terms of other chain properties as

(1) $$\tau_{R}=\frac{\zeta N\langle R^{2}\rangle }{3\pi^{2}k_{B}T}.$$

Here, ζ is a bead drag coefficient, *N* is the number of beads in the polymer, $\langle R^{2}\rangle$ is the polymer’s mean-square radius of gyration, $k_{B}$ is Boltzmann’s constant, and *T* is the absolute temperature.

Between $\tau_{R}$ and a *disengagement time* $\tau_{d}$, the polymer diffuses back and forth along the length of its tube. The tube’s ends fray and disappear whenever the polymer backs away from them. Over times greater than $\tau_{d}$, the polymer is no longer confined by the tube and instead performs simple diffusion. The tube model indicates that it does not describe the motion of short chains. Instead, for short chains, there are only two time regimes, as described by the Rouse model, the time regimes being separated by the time $\tau_{R}$.

Tube models of polymer dynamics make a series of predictions that are accessible to simulative tests. Of particular interest are predictions of mean-square displacements, including displacements of individual beads and displacements of the chain center of mass. The atomic mean-square displacement for a chain is described by

(2) $$g_{1}\left(t\right)=\frac{1}{N}\sum_{i=1}^{N}\langle |r_{i}\left(t+t_{0}\right)-r_{i}\left(t_{0}\right){|}^{2}\rangle .$$

Here, Looks correct to me $t_{0}$ and $t+t_{0}$ are times. The sum may be over the positions $r_{i}\left(t_{0}\right)$ of all *N* beads of a chain, or over the positions of a subgroup of *n* beads in a longer chain. Motions of a subgroup centered on the chain center are typically considered. The average 〈⋯〉 includes averages over all chains and over all values of the initial time $t_{0}$.

The center-of-mass mean-square displacement is

(3) $$g_{3}\left(t\right)=\langle |r_{cm}\left(t+t_{0}\right)-r_{cm}\left(t_{0}\right){|}^{2}\rangle ,$$

where $r_{cm}\left(t\right)$ is the position of a chain center of mass at time *t*, the average being over all chains and all initial times $t_{0}$. Center-of-mass displacements have been reported using the center of mass of all beads in a chain or separately by using the center of mass of the central beads of a chain.

The mean-square displacement of monomer beads relative to their polymer’s center of mass $r_{cm}\left(t\right)$ is described by

(4) $$g_{2}\left(t\right)=\frac{1}{N}\sum_{i=1}^{N}\langle |r_{i}\left(t+t_{0}\right)-r_{cm}\left(t+t_{0}\right)-\left(r_{i}\left(t_{0}\right)-r_{cm}\left(t_{0}\right)\right){|}^{2}\rangle ,$$

where *n* is the number of beads in a chain.

When needed, we refer to the mean-square displacement functions collectively as g(t).

The Rouse and tube models predict the time dependence of these mean-square displacements, the predictions all having the form $g\left(t\right)∼t^{\alpha }$. The exponent α depends on the polymer chain length and the time scale over which the time dependence is being described.

For melts of short chains, the tube model predicts that polymers have the properties predicted by the Rouse model, namely,

(5) $$\begin{array}{ccc} g_{1}\left(t\right) & ∼ & t^{1/2}\;\;\mathrm{for}\;\;t\leq \tau_{R}, \end{array}$$

(6) $$\begin{array}{ccc} g_{1}\left(t\right) & ∼ & t\;\;\mathrm{for}\;\;t\geq \tau_{R}, \end{array}$$

(7) $$\begin{array}{ccc} g_{3}\left(t\right) & ∼ & t\;\;\mathrm{for}\;\;t\leq \tau_{R} \end{array}$$

(8) $$\begin{array}{ccc} g_{3}\left(t\right) & ∼ & t\;\;\mathrm{for}\;\;t\geq \tau_{R}. \end{array}$$

At times $t<\tau_{R}$, chain motions in a polymer melt are said to be described by the relaxation of pure Rouse modes, leading to α=1/2 for $g_{1}\left(t\right)$ and α=1 for $g_{3}\left(t\right)$. At longer times, $t>\tau_{R}$, α=1 is predicted both for $g_{1}\left(t\right)$ and for $g_{3}\left(t\right)$.

For long chains, the tube model predicts for single-bead mean-square displacements that

(9) $$\begin{array}{ccc} g_{1}\left(t\right) & ∼ & t^{1/2}\;\;\mathrm{for}\;\;t\leq \tau_{e}, \end{array}$$

(10) $$\begin{array}{ccc} g_{1}\left(t\right) & ∼ & t^{1/4}\;\;\mathrm{for}\;\;\tau_{e}\leq t\leq \tau_{R}, \end{array}$$

(11) $$\begin{array}{ccc} g_{1}\left(t\right) & ∼ & t^{1/2}\;\;\mathrm{for}\;\;\tau_{R}\leq t\leq \tau_{d}, \end{array}$$

(12) $$\begin{array}{ccc} g_{1}\left(t\right) & ∼ & t\;\;\mathrm{for}\;\;\tau_{d}\leq t. \end{array}$$

$g_{2}\left(t\right)$ and $g_{1}\left(t\right)$ have the same behavior until $t=t_{d}$, where $\tau_{d}$ is the typical time required for a chain to escape its tube. At later times, $g_{2}\left(t\right)$ reaches a plateau at $g_{2}\left(t\right)=R_{g}^{2}$.

For long chains, the tube model predicts for center-of-mass mean-square displacements that

(13) $$\begin{array}{ccc} g_{3}\left(t\right) & ∼ & t\;\;\mathrm{for}\;\;t\leq \tau_{e}, \end{array}$$

(14) $$\begin{array}{ccc} g_{3}\left(t\right) & ∼ & t^{1/2}\;\;\mathrm{for}\;\;\tau_{e}\leq t\leq \tau_{R} \end{array}$$

(15) $$\begin{array}{ccc} g_{3}\left(t\right) & ∼ & t\;\;\mathrm{for}\;\;\tau_{R}\leq t. \end{array}$$

The $t^{1/4}$ regime for long chains during $\tau_{e}\leq t\leq \tau_{R}$ is often viewed as a signature that the tube model is correct. However, as noted by Puetz et al. [5], in the same time regime, the Schweizer mode-coupling model [6,7] predicts a very similar $t^{0.28}$ time dependence for $g_{1}\left(t\right)$, even though Schweizer’s model gives an entirely different description of bead motion. Similarly, in the following regime, $g_{1}\left(t\right)$ is predicted by the tube model to be proportional to $t^{1/2}$, while the Schweizer model predicts $t^{0.56}$. From $g_{1}\left(t\right)$, it may therefore be difficult to distinguish between the tube and Schweizer models.

A few authors calculate the full distribution P(r,t) of displacements *r* at each time *t*. When P(r,t) is non-Gaussian, a non-Gaussianity parameter $\alpha_{2}\left(t\right)$ has been introduced. $\alpha_{2}\left(t\right)$ is defined as

(16) $$\alpha_{2}\left(t\right)=\frac{3\langle r^{4}\left(t\right)\rangle -5{\langle r^{2}\left(t\right)\rangle }^{2}}{5{\langle r^{2}\left(t\right)\rangle }^{2}}.$$

If $\alpha_{2}\left(t\right)\neq 0$, interpreting $g_{1}\left(t\right)$ as supplying a (time-dependent) diffusion coefficient is invalid, because particle motion does not have the Gaussian displacement distribution required by Doob’s theorem [8] if the particle motion is Brownian diffusion. In addition, if $\alpha_{2}\left(t\right)\neq 0$, it would be incorrect to relate $g_{1}\left(t\right)$ to the dynamic structure factor S(q,t) by using a Gaussian approximation $S\left(q,t\right)∼\exp(-q^{2}g_{1}\left(t\right)/2)$, because that form is only correct if particle displacements have a Gaussian distribution of values at all times [9]. If P(r,t) is not a Gaussian, the Gaussian approximation for S(q,t) may be incorrect even as an order-of-magnitude approximation.

Here, we consider simulated determinations of polymer mean-square displacements and what they say about the validity of the deGennes–Doi–Edwards tube model and other models of polymeric fluids. Our focus is on a careful numerical study of the time-dependent mean-square displacements, comparing with predictions, covering chains of all lengths, showing that there are regimes in which the mean-square displacements $g_{1}\left(t\right)$, $g_{2}\left(t\right)$, and $g_{3}\left(t\right)$ have power-law dependence $t^{\alpha }$ on time. Our interest here is less which exponents are found and more whether or not a power-law dependence is present at all.

How might power-law dependence be demonstrated? In the simplest case, if there were a power-law dependence $g\left(t\right)∼t^{\alpha }$, a graph of log(g(t)) against log(t) would reveal a straight line having slope α. Multiple straight-line regions might well be separated by smooth curves marking crossover regimes. Here, we replace this crude graphical approach with a systematic quantitative method.

This paper represents an extension of our prior reviews on the phenomenology of the dynamics of polymeric liquids. In our prior volume *Phenomenology of Polymer Solution Dynamics* [10], we considered the concentration, molecular weight, and time dependence of polymer solution properties, including centrifugation, electrophoresis, polarized and depolarized light scattering spectroscopy, solvent diffusion, segmental relaxation, dielectric relaxation, mutual diffusion, probe diffusion, viscosity, viscoelasticity, non-linear viscoelastic behavior, and outcomes of the same techniques as applied to solutions of spherical colloids. A systematic comparison of experiments on polymer solutions with theoretical models found that hydrodynamic scaling models [11] worked well, but reptation-scaling forms [2], e.g., $D∼c^{\nu }M^{\delta }$ for the self-diffusion coefficient, ν and δ being constants, were uniformly inconsistent with experiments. At that time, we were challenged to discuss whether our results applied to melts but were not prepared to do so. This paper is part of a series now responding to that challenge. In a recent paper [12], we extended our reviews to simulations of polymer melts, notably the behavior of the nominal Rouse modes, finding that Rouse mode amplitudes of polymers in melts do not have the properties predicted by Rouse. Here, we continue this study, turning to the behavior of the mean-square displacements g(t) as found in polymer simulations.

The remainder of this paper has three sections. The next section presents our quantitative method for analyzing g(t) to determine actual time dependence. We previously presented tests of the method against representative data [1]. The following section applies these quantitative methods to results from the simulation literature. A final section discusses what is found. The verisimilitude of our analysis arises from the graphical presentations of our numerical analyses, so the figures in Supplementary Materials present at full scale all of our results.

## 2. Methods

In this paper, we re-analyze literature reports of the g(t) of simulated polymeric systems. Most measurements in the literature are reported as smooth curves, not as the discrete points normally generated by molecular dynamics simulations. The digitization of reported g(t) determinations was accomplished using UN-SCAN-IT (Silk Scientific, Provo, UT, USA) under manual control. The significant obstacle, at least in some papers, was that the authors conserved space by plotting multiple sets of results on a single graph. In some cases, data points were sufficiently overlapped that determinations of g(t) became impossible.

We advance by curve fitting. Identifying y(t)=log(g(t)) and x=log(t), we fit log(g(t)) to polynomials

(17) $$y\left(t\right)=\sum_{n=0}^{N}k_{n}x^{n}.$$

Here, the $k_{n}$ are the fitting parameters, to be obtained from a linear least-mean-squares analysis, and *N* is the truncation limit, the largest value of *n* in a given fit. Which value of *N* is appropriate? We repeatedly fit measurements to Equation (17) with progressively increasing values of *N*. The mean-square difference between the fitted curve and the measurements was obtained. We consistently found that there was an upper limit on *N* above which further increases in *N* gave at most a very limited reduction in the mean-square difference between the simulative measurements and Equation (17). N=8 was generally used as the cutoff in the studies here. As seen below and in the Supplemental Materials, our fitted polynomials accurately followed simulative determinations of the g(t), with no indication of a need to fit different time regimes with distinct polynomials employing different sets of $k_{n}$.

In our analysis,

(18) $$K_{1}=\frac{dy(t)}{dx}.$$

is the logarithmic derivative dlog(g(t))/dlog(t) of our polynomial. For each *t*, $K_{1}$ gives the instantaneous value of α in a local-in-time scaling law $g\left(t\right)∼t^{\alpha }$. If power-law behavior were present in some time regime, within that regime, $K_{1}$ would be constant up to noise. To test for constancy, we also calculated the second logarithmic derivative

(19) $$K_{2}=\frac{d^{2}y\left(t\right)}{dx^{2}}.$$

In general, $K_{1}$ and $K_{2}$ both depend on *t*. In a power-law regime, one expects $K_{2}\approx 0$ over an extended range of times. Noise in the simulation, the digitization process, and the numerical fitting steps lead to noise in $K_{2}$, so in a power-law regime, $K_{2}$ is in general not exactly zero. $K_{2}\approx 0$ is also found over a narrow range of times wherever $K_{1}$ has a maximum, a minimum, or a saddle point, but these cases are readily distinguished from power-law behavior.

Our analytical approach appears to be more sophisticated than what has oft-times been applied to identify power-law regimes. This observation is not meant as a criticism of earlier work. The earlier processes were entirely adequate for the needs of their users. We only claim that our more powerful mathematical technique reveals hitherto obscure features that were actually present in the original studies. In particular, we do not attempt to force the interpretation that $g\left(t\right)∼t^{\alpha }$, i.e., that log(g(t)) actually has linear dependence on log(t) over some range of times. We instead determine the dependence of log(g(t)) on log(t) and inquire as to whether that dependence is linear in log(t), as would be the case if $g\left(t\right)∼t^{\alpha }$.

Our procedure creates smooth curves that, at least in the studies discussed here, always visibly pass within noise through the measured g(t). The logarithmic derivative $K_{1}$ of the fitted function provides, for each time, the local value of the exponent, as could in principle be obtained by a fit of a power law to log(g(t)) over a narrow range of log(t). As a practical matter, we found that direct fits of $t^{\alpha }$ to g(t) over short ranges in time were quite noisy, so we tried but did not pursue examining $K_{1}$ as obtained from a multitude of local fits. In contrast, logarithmic derivatives of Equation (17) are nearly noiseless. This distinction between our approach and local fits is the well-known issue that, while numerical integration tends to suppress noise, numerical derivatives tend to amplify noise. We emphasize that polynomial fits are interpolants, not extrapolants. They fill in the space between measured points but are unreliable as predictors outside of the range of times over which the fit was made.

## 3. Analysis

In this section, we use our numerical analysis approach to consider g(t) as reported by a series of authors. Strong conclusions, as presented here, need strong support. We therefore include eighty determinations of g(t). While we could have selected from these an exemplary set of a few systems, this path would have led to suggestions that we had cherry-picked data to support our conclusions. On the other hand, there is a certain uniformity in the time dependence of g(t) and of $K_{1}$. In almost every case: g(t) increases monotonically on a log–log plot more rapidly at early and late times and less rapidly at intermediate times. With increasing time, the first logarithmic derivative $K_{1}$ decreases more or less smoothly to a minimum, corresponding to an inflection point of g(t), and then increases more or less smoothly again. In a very few cases, we find that $K_{1}$ has a single extended region in which it is constant, corresponding to a power-law dependence of g(t) on *t*. In no system do we observe more than one power-law regime for g(t), other than at the short- or long-time limits. Turning to individual studies:

Behbahani and Schmid [13] reported simulations of a Grest–Kremer bead–spring polymer model [14]. We thank them for supplying us with numerical tables of g(t) as determined by their simulations. Beads had mass *m* and a purely repulsive Weeks–Chandler–Anderson potential [15] with length scale σ and energy ϵ. Bead–bead bonds were represented with a FENE (Finite Extensible Nonlinear Elastic) potential [16]. Studies were conducted on polymers having between 5 and 1000 beads with a minimum of 192 chains in a simulation box. Time was measured in natural units $\tau ={\left(m\sigma^{2}/\epsilon \right)}^{1/2}$, simulations being extended out from $10^{5}\tau$ to $10^{8}\tau$ depending on chain lengths. Behbahani and Schmid noted a series of estimates of the entanglement length, one group of estimates in a cluster near 50 beads and another in a cluster of 80 or a few more beads. The authors used the former number to estimate that their thousand-bead polymer had 20 entanglements on average. The estimated distance between entanglements, which was identified as the tube diameter *a*, was estimated to be approximately 9σ. From their simulations, Behbahani and Schmid calculated the mean-square bead displacements, the mean-square displacements of the central beads, the mean-square center-of-mass displacements, the time dependence of the polymer end-to-end vector, the single-chain dynamic structure factor, the stress relaxation modulus, and the zero-shear viscosity.

**Figure 1:**
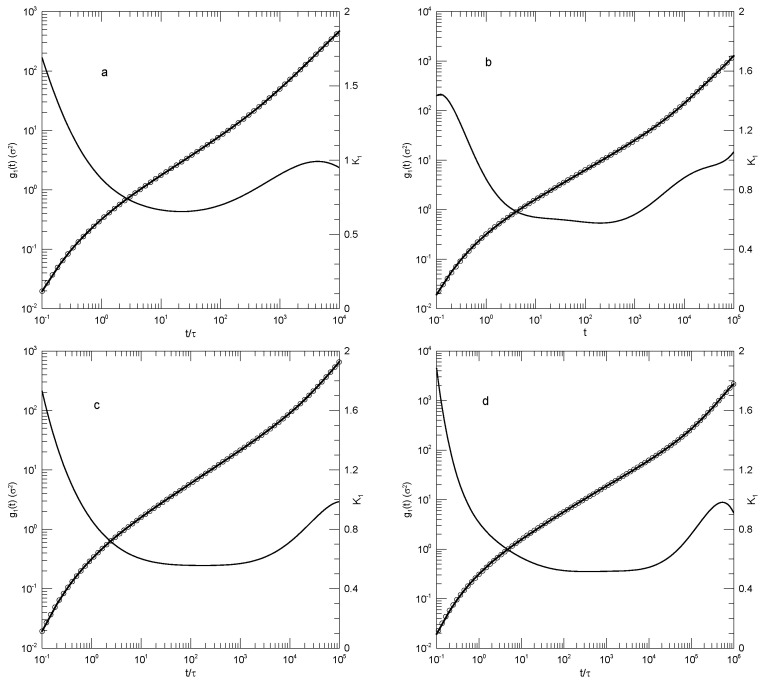
Mean–square bead displacements $g_{1}\left(t\right)$ (thick lines) of melts of short Kremer–Grest bead–spring chains, based on the simulations of Behbahani and Schmid [13], together with fits to eighth-order polynomials (circles) and the corresponding first logarithmic derivatives $K_{1}$ (thin lines). Chains contained (**a**) 10, (**b**) 30, (**c**) 50, or (**d**) 100 beads.

**Figure 2:**
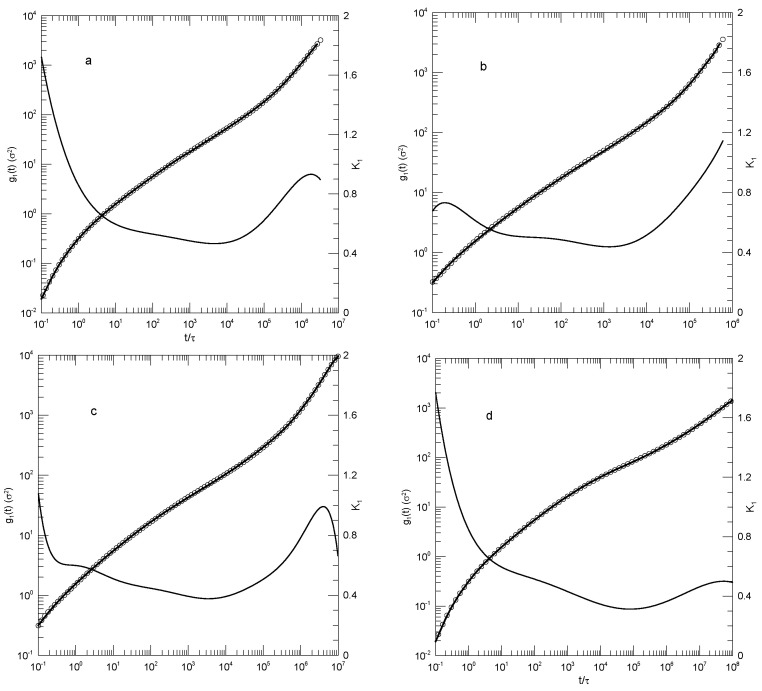
Mean –square bead displacements $g_{1}\left(t\right)$ (thick lines under circles) of melts of long Kremer–Grest bead–spring chains, based on the simulations of Behbahani and Schmid [13], together with polynomial fits (circles) and the corresponding first logarithmic derivatives $K_{1}$ (thin lines). Chains contained (**a**) 150, (**b**) 200, (**c**) 400, or (**d**) 1000 beads. Polynomial fits were to eighth-order polynomials via linear least squares, except for the 400-bead polymers, for which a tenth-order polynomial was used.

We first considered single-bead motions. Figure 1 and Figure 2 show Behbahani and Schmid’s determinations of mean-square single-bead displacements $g_{1}\left(t\right)$ in their systems, and our fits of $\log(g_{1}\left(t\right))$ to a power series in log(t). We used fits to eighth-order polynomials, except that in one system (the 400-bead polymer, Figure 2c) the eighth-order fit to $g_{1}\left(t\right)$ was not entirely satisfactory, so a tenth-order fit was used instead. Which features were revealed by $K_{1}$? For the 10-bead chains, $K_{1}$ had a minimum, and correspondingly, $g_{1}\left(t\right)$ had an inflection point near t=22. For longer chains, there appeared to be two features that emerged and separated with increasing chain length. The 1000-bead chains showed these clearly. First, $K_{1}$ had a minimum, so $g_{1}\left(t\right)$ had an inflection point. Second, at shorter times before reaching its minimum, $K_{1}$ had an extended region in which it decreased linearly with log(t) (and, correspondingly, $K_{2}$ was a constant). For shorter chains, the near-linear region acquired weak distortions from linearity. Finally, for the 100- and 50-bead polymers, the linear region and the minimum in $K_{1}$ blended into each other, so that one could reasonably describe the region near the apparent inflection point as showing power-law behavior. The value of $K_{1}$ at its minimum, the slope of $\log(g_{1}\left(t\right))$ at the inflection point, decreased with the increasing chain length, from 0.65 for the 10-bead chains to 0.31 for the 1000-bead chains. Over the same series of simulations, the location of the minimum increased, from t/τ=22 for the 10-bead chains to $t/\tau =8.1\times 10^{4}$ for the 1000-bead chains. Table 1 shows the full dependence of $K_{1}$ on chain length.

Close to the shortest and longest times studied, we sometimes saw an additional feature in the time dependence of $K_{1}$. As seen in Figure 1a,d and Figure 2a,c, the feature was a local maximum in $K_{1}$ in these figures, located just before the largest times studied. We noted the feature but were not certain whether it was real or a consequence of the operation of our polynomial fitting process near end points.

**Figure 3:**
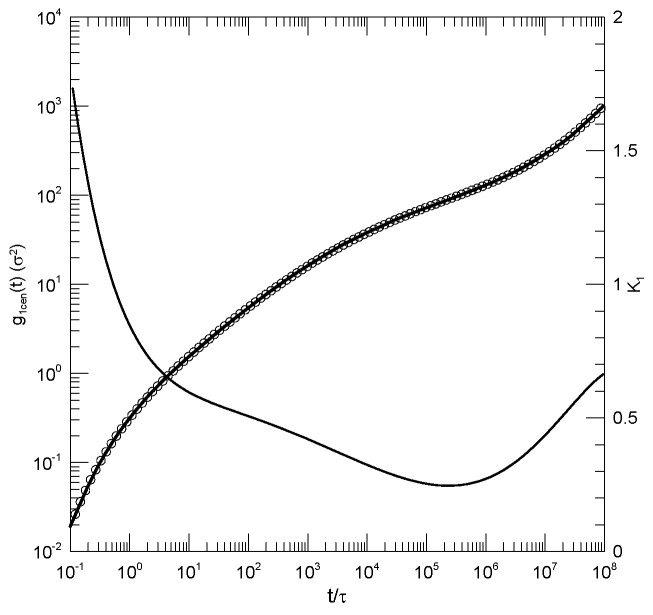
Mean-square bead displacements $g_{1}\left(t\right)$ of central beads (thick lines under circles) of melts of 1000-bead Kremer–Grest bead–spring chains, based on simulations of Behbahani and Schmid [13], together with a fit to an eighth-order polynomial (circles) and its first logarithmic derivatives $K_{1}$ (thin lines).

Behbahani and Schmid also reported $g_{1}\left(t\right)$ as calculated using only the displacements of the central beads of the polymer. It is generally stated that polymer end beads are more mobile than beads near the center, for reasons not arising from core parts of the reptation-tube model, so a study of central beads may well be a better test of that model. Figure 3 shows their results for the 1000-bead polymer, and our fit to their results. $\log(g_{1}\left(t\right))$ for central beads indeed had an inflection point (a minimum in $K_{1}$) for $K_{1}\approx 0.25$, this being the slope predicted by the reptation-tube theory as a central outcome of the model. Those authors justifiably proposed that they observed the predicted $t^{1/4}$ region emerging from surrounding transition regions. One notes, however, that the minimum of $K_{1}$ progressively decreased with the increasing polymer chain length, so a needed experiment would study somewhat longer polymers, say 1300 or 1500 beads, to determine whether for longer chains, the minimum in $K_{1}$ remained near $K_{1}\approx 0.25$, or whether with an increasing chain length, the minimum in $K_{1}$ continued to decrease, in which case the observed 0.25 would be a coincidence arising from a fortunate choice of chain length. As with $g_{1}\left(t\right)$, at times shorter than that at which the inflection point was seen, there was an extended region in which $\log(g_{1}\left(t\right))$ decreased linearly with log(t). The physical interpretation of this observation is unclear.

**Figure 4:**
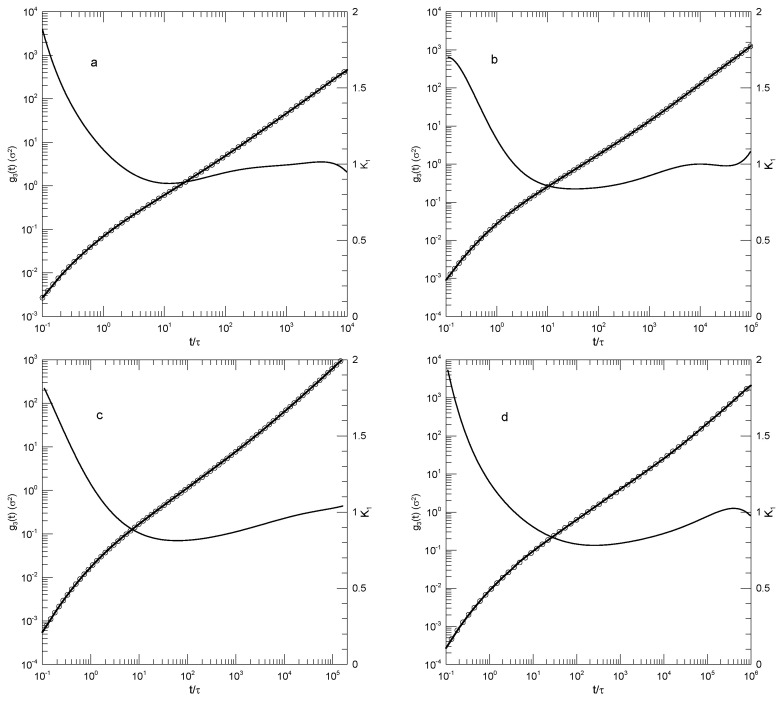
Mean-square center-of-mass displacements $g_{3}\left(t\right)$ (thick lines under circles) of melts of short Kremer–Grest bead–spring chains, based on simulations of Behbahani and Schmid [13], together with polynomial fits (circles) and their first logarithmic derivatives $K_{1}$ (thin lines). Chains contained (**a**) 10, (**b**) 30, (**c**) 50, or (**d**) 100 beads. Polynomial fits were to eighth-order polynomials via linear least squares.

**Figure 5:**
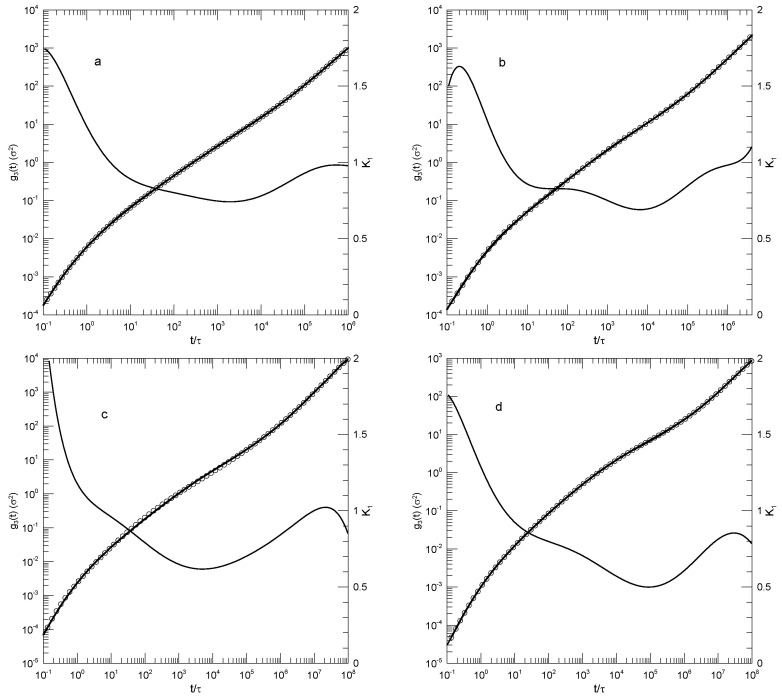
Mean-square center-of-mass displacements $g_{3}\left(t\right)$ (thick lines under circles) of melts of long Kremer–Grest bead–spring chains, based on simulations of Behbahani and Schmid [13], together with polynomial fits (circles) and their first logarithmic derivatives $K_{1}$ (thin lines). Chains contained (**a**) 150, (**b**) 200, (**c**) 400, or (**d**) 1000 beads. Polynomial fits were to eighth-order polynomials via linear least squares.

We finally considered Behbahani and Schmid’s determinations of center-of-mass motions. Figure 4 and Figure 5 show their measurements of mean-square center-of-mass displacements $g_{3}\left(t\right)$. Once again, the time dependence of $K_{1}$ showed that for each polymer length, $g_{3}\left(t\right)$ had an inflection point. The slope $K_{1}$ at the inflection point decreased with the increasing polymer length, from $K_{1}=0.87$ for the 10-bead polymers to $K_{1}=0.50$ for the 1000-bead polymers. With increasing chain length, the time at which the inflection point was found also increased, from t≈12 for the 10-bead polymer to $t\approx 8.8\times 10^{4}$ for the 1000-bead polymer. At times before the inflection point, $K_{1}$ had distinct short- and long-chain behaviors. For the shorter chains, ≤150 beads per chain, $K_{1}$ decreased smoothly with a decreasing slope until it reached the inflection point. For the longer chains, 200 beads or more per chain, $K_{1}$ acquired a shoulder covering 2–3 decades in time before it reached its minimum.

Chang and Yethiraj [17] explored a polymer model in which polymer molecules may or may not be allowed to pass through each other, depending on the value of a single parameter. They modeled polymers as chains of hard spheres having diameter σ coupled by bonds whose lengths could fluctuate freely over a range of lengths $(1\pm \delta_{B})\sigma$. Beads interacted via elastic collisions. In different simulations, chains had lengths *N* between 8 and 512 beads at bead volume fractions of 0.3 or 0.4, with $\delta_{B}$ ranging from 0.05 to 0.9. Chain crossings were blocked for $\delta_{B}\leq 0.4$, marginally possible for $\delta_{B}=0.45$, and became more frequent as $\delta_{B}$ was further increased. They calculated a diffusion coefficient *D* for the chains, finding that DN was approximately constant for N<64 but decreased at larger *N* and inferred that N≈64 marked the onset of entanglement. Static chain properties, the pair correlation function, and the static structure factor were nearly independent of $\delta_{B}$. Chain dynamic properties were sensitive to the chain crossing parameter, showing one behavior for $\delta_{B}\geq 0.5$ and a different behavior for $\delta_{B}\leq 0.45$. In particular, for long chains, the chain self-diffusion coefficient scaled as 1/N for large $\delta_{B}$ and as $1/N^{2}$ for small $\delta_{B}$.

Of particular interest here, Chang and Yethiraj [17] calculated the mean-square displacements of the chain centers of mass and the chain’s central monomers for $\delta_{B}=0.4$, 0.45, 0.5, and 0.6. The 0.4 and 0.45 results were nearly indistinguishable. These were the chain nearly non-crossing simulations, so we considered only the $\delta_{B}=0.45$ simulation.

**Figure 6:**
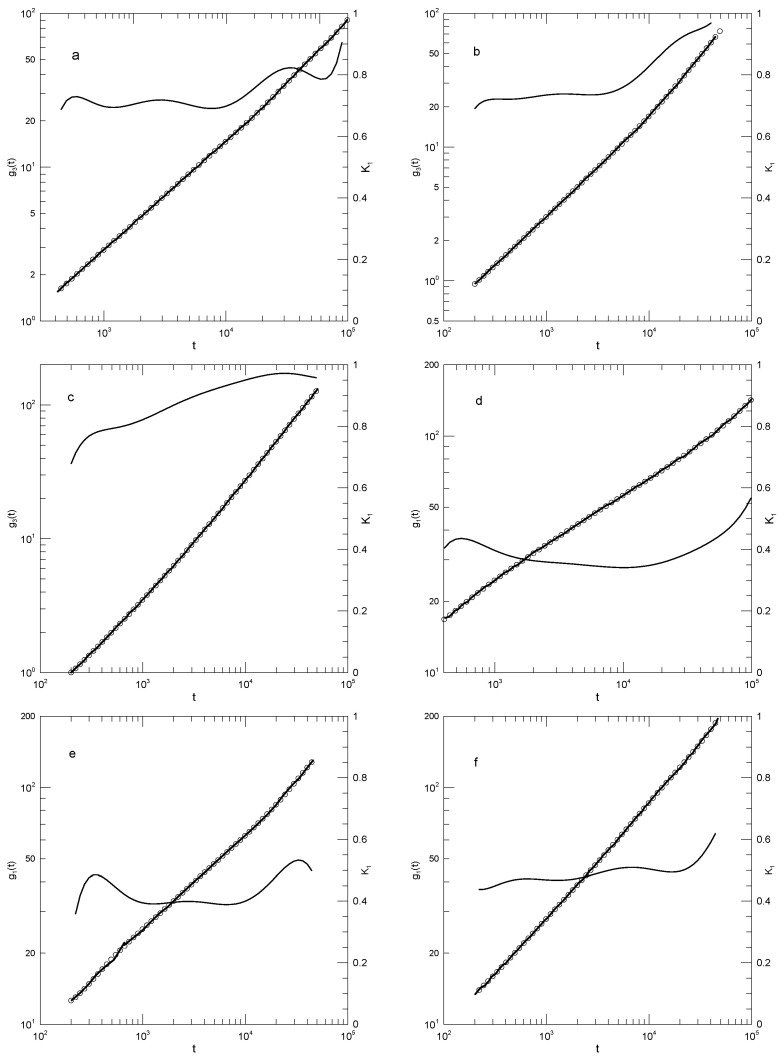
Mean-square center-of-mass (**a**–**c**) and central-bead (**d**–**f**) mean-square displacement measurements (heavy lines) of a melt of 256-bead chains, based on the simulations of Chang and Yethiraj [17], together with polynomial fits (circles) and first logarithmic derivatives $K_{1}$ (thin lines). Here, $\delta_{B}$ takes values (**a**,**d**) 0.4 or 0.45, (**b**,**e**) 0.5, and (**c**,**f**) 0.6. Polynomial fits were to eighth-order polynomials via linear least squares.

As seen in Figure 6, Chang and Yethiraj’s [17] determinations of g(t) were all described well by polynomial curves. The first derivatives were equally well behaved. Beginning with the center-of-mass displacement measurements as described by $g_{3}\left(t\right)$: As seen in Figure 6a, for $\delta_{B}=0.45$, $K_{1}$ increased smoothly from approximately 0.7 to 0.8 with increasing *t*, and at the largest *t*, it appeared to increase to 0.9 or so. Figure 6b reveals for $\delta_{B}=0.5$ that at earlier times, $K_{1}\approx 0.74$ was nearly constant, increasing by 0.03 over its near-constant range; at t/τ>4000, $K_{1}$ increased progressively toward $K_{1}=1$. From Figure 6c, for $\delta_{B}=0.6$, $K_{1}$ at first increased rapidly and then rose gradually from 0.8 to a plateau near 0.95.

Chang and Yethiraj [17] also calculated $g_{1}\left(t\right)$ for the central monomers of their chains. For $\delta_{B}=0.45$, the corresponding $K_{1}$ lay in the range 0.35–0.40, except at the longest and shortest times reported, as seen in Figure 6d. Figure 6e gives the results for $\delta_{B}=0.5$, for which $K_{1}$ was consistently near 0.39, except at the two extrema, where it rose to 0.45 or 0.55. On increasing $\delta_{B}$ to 0.6, $K_{1}$ became larger and increased smoothly from 0.45 to 0.5 over most of the observed time range, with a modest further increase to about 0.6 at the largest times studied.

Chang and Yethiraj proposed that the power-law time regimes of the reptation-scaling model were seen in their central-bead results for $\delta_{B}=0.4$, these results being shown in Figure 6d. In that figure, we find that $g_{3}\left(t\right)$ follows a smooth curve with a not-quite-constant slope in the range 0.35–0.4, the slope never decreasing to the 0.25 of the scaling model.

**Figure 7:**
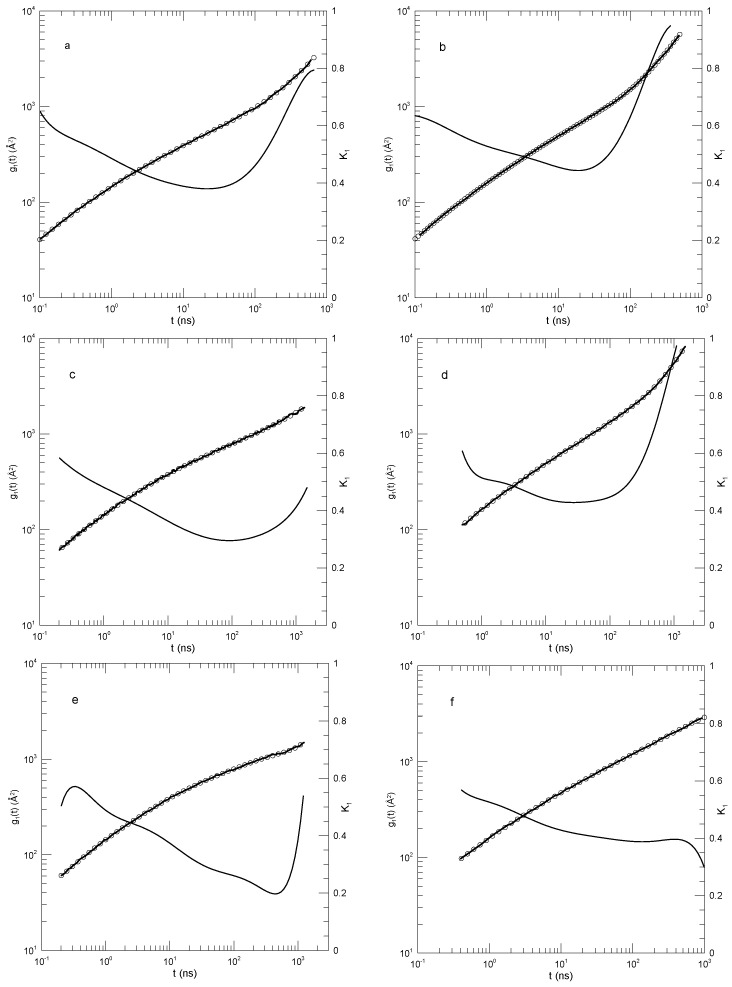
Mean-square bead displacements (heavy lines) of linear (**a**,**c**,**e**) and ring (**b**,**d**,**f**) polyethylene oxides, as determined by the united-atom simulations of Tsalikis et al. [18], together with polynomial fits (circles) and their first logarithmic derivatives $K_{1}$ (thin lines). Polymer lengths were 120 beads (**a**,**b**), 227 beads (**c**,**d**), and 455 beads (**e**,**f**). Polynomial fits were to eighth-order polynomials via linear least squares.

Tsalikis et al. [18] reported simulations of ring and linear polyethylene oxide molecules in the melt. They examined monodisperse systems of chains having 120, 227, or 455 monomers, corresponding to molecular weights of 5330, 10,044, or 20,086 Da, which they referred to as the PEO-5K, PEO-10K, and PEO-20K melts. For their systems, they estimated $N_{e}\approx 45$. Simulations were carried out using Fischer’s united-atom force field [19]. For the PEO-20K melt, the simulation cell contained more than 50,000 united atoms. Tsalikis et al. reported detailed studies of static and dynamic properties of their polymers, including $g_{1}\left(t\right)$. Figure 7 shows $g_{1}\left(t\right)$ from their Figure 13, our fits to their simulation data, and the corresponding first derivatives. The ring data correspond to mean-square displacements of all monomers, while the linear chain data are for the “most central” (their words) monomers. They terminated their measurements at the Rouse times for each system. At each molecular weight, the linear chain’s central monomers moved more rapidly than the monomers of the corresponding ring. Tsalikis, et al. interpreted their results on the PEO-5K and PEO-10K systems as showing two distinct breaks in $g_{1}\left(t\right)$, the lower being near 2 nS and the higher being at 150 or 490 nS.

In Figure 7a, the 120-bead linear molecules, the slope $K_{1}$ falls from 0.6 at 0.2 nS to 0.38 at 22 nS and then rises again to 0.8 at the longest time reported. $K_{1}=0.38$ is visibly a local minimum. Figure 7b shows the corresponding results for the 120-bead ring polymer. As a function of time, $K_{1}$ had a minimum at 20 nS, very nearly the same time as for the linear polymer, but here, $K_{1}$ was 0.44. At longer times, $K_{1}$ rose to 0.95, which did not appear to be a maximum. Figure 7c,d show the corresponding results for the 227-bead linear and ring polymers. In these two systems, $K_{1}$ had minima at 0.30 and 0.43, respectively. For the ring polymer, a case can be made that there is a region within which $K_{1}$ is nearly constant, namely, $K_{1}$ was within 1% of its 0.43 minimum for 10≤t≤80 nS. Figure 7e,f, for the 455-bead linear and ring polymers, somewhat repeat the behavior seen for the 227-bead polymers. For the linear 455-bead polymer, $K_{1}$ fell from 0.57 at 0.33 nS to a minimum of 0.2 near 450 nS and then rose steeply at later time delays. The 455-bead ring polymer, like its 227-bead ring counterpart, had an extended minimum between 70 and 250 nS with $K_{1}\approx 0.39$.

For each of the larger rings, we therefore found a single power-law region, though not with the same exponent in the two cases. With an increasing chain length, the time dependence of $g_{1}\left(t\right)$ softened, but key characteristic features only changed numerically. In describing their results, Tsalikis et al., instead said that there were clear breaks in $g_{1}\left(t\right)$ separating extended regions in which $g_{1}\left(t\right)$ followed one power law or another. We did not see these multiple power-law regions in our analysis.

Takahashi et al. [20] reported extended simulations of a united-atom model for polyethylene. They used the TraPPE-UA model, including Lennard–Jones potentials between non-bonded atoms, rigid bonds using the LINCS algorithm, bond bending with a harmonic potential, and torsional potentials along the chain backbones. Chain molecular weights ranged from 423 to 2807 Da, with 150 to 1000 chains in a periodic box. They estimated $N_{e}\approx 80$ or equivalently $M_{e}\approx 1100$, implying that their chains were unentangled or slightly entangled, with at most 2–3 entanglements on a single chain. The mean-square displacement $g_{1}\left(t\right)$ of the chain’s central bead was calculated as a function of time. From the time correlation function of the chain end-to-end vector, a nominal relaxation time $\tau_{R}$ was inferred. A transition in the molecular weight dependence of $\tau_{R}$ was observed near a molecular weight of 1000, with $\tau_{R}∼M^{2.1}$ at smaller molecular weights and $\tau_{R}∼M^{2.7}$ at larger molecular weights.

**Figure 8:**
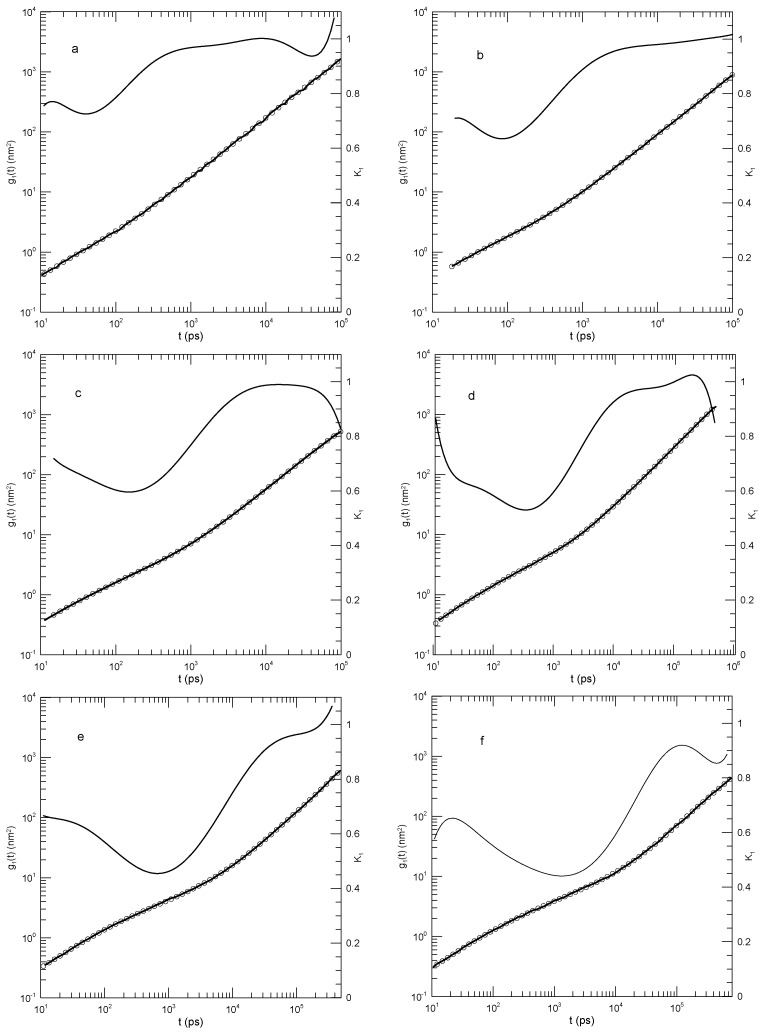
Mean-square bead displacements (thick lines) of linear polyethylenes, nominal molecular weights (**a**) 422, (**b**) 703, (**c**) 983, (**d**) 1405, (**e**) 2106, and (**f**) 2807 Da, from Takahashi et al. [20], together with eighth-order polynomial fits (circles) and their first logarithmic derivatives $K_{1}$ (thin lines).

Figure 8 shows our digitization of their measurements of $g_{1}\left(t\right)$, our polynomial fits to those measurements, and the computed logarithmic first derivatives $K_{1}$. Agreement between the measured $g_{1}\left(t\right)$ values and the polynomial fits was uniformly excellent. Of particular interest is Figure 8b. At longer times, $>10^{3}$ pS, $\log(g_{1}\left(t\right))$ lay on a straight line having slope unity. Correspondingly, the plot of $K_{1}$ against time shows a straight horizontal line, confirming that $g_{1}\left(t\right)$ exhibited power-law behavior with $g_{1}\left(t\right)∼t^{1}$. This result confirms that our method does reveal power-law behavior when it occurs. At shorter times, the slope of $g_{1}\left(t\right)$ in each of these systems had a local minimum, corresponding to $g_{1}\left(t\right)$ having a shorter-time inflection point. The slope at the inflection point decreased with the increasing polymer molecular weight, the slopes at the six minima in order of increasing polymer length being 0.73, 0.64, 0.60, 0.53, 0.46, and 0.44, respectively.

For the 2807 Da polymer, Takahashi et al. noted that the sequence of straight-line slopes from the reptation-scaling model approximately resembled the curving g(t), except that the “…threshold values …”, the moments in time at which one transitions from one power law to the next, were “…unclear …” in the $g_{1}\left(t\right)$ graph. We agree with their conclusion, namely, that except at the longest time, $g_{1}\left(t\right)$ follows a smooth curve with no “transitions”.

Peng et al. [21] reported simulations of dynamically asymmetric A–B blends as functions of chain lengths and concentrations. Under some conditions, the A and B chains became immiscible. The authors studied both phase-separated systems and systems which did not phase separate over the duration of their simulations at the simulated temperature. Their polymer beads interacted via a Lennard–Jones potential and, along each chain, a FENE potential. A and B chains all used the same Lennard–Jones and FENE parameters. The A chains were made stiffer by endowing them with a harmonic bond-bending potential and a torsional potential. B chains had a fixed length $N_{B}=50$; A chains had lengths in the range 3–100. Nominal entanglement lengths were given as 28–45 for the A chains and 85 for the B chains, so the B chains were not entangled and the A chains would at most have been weakly entangled. The simulation cells all contained approximately 40,000 beads, all having the same mass. Simulations calculated an extended series of dynamic properties, including bead and center-of-mass mean-square displacements, Rouse-mode temporal autocorrelation functions, and deviations from a Gaussian distribution of displacements, as represented by the non-Gaussianity parameter $\alpha_{2}\left(t\right)$ of Equation (16).

**Figure 9:**
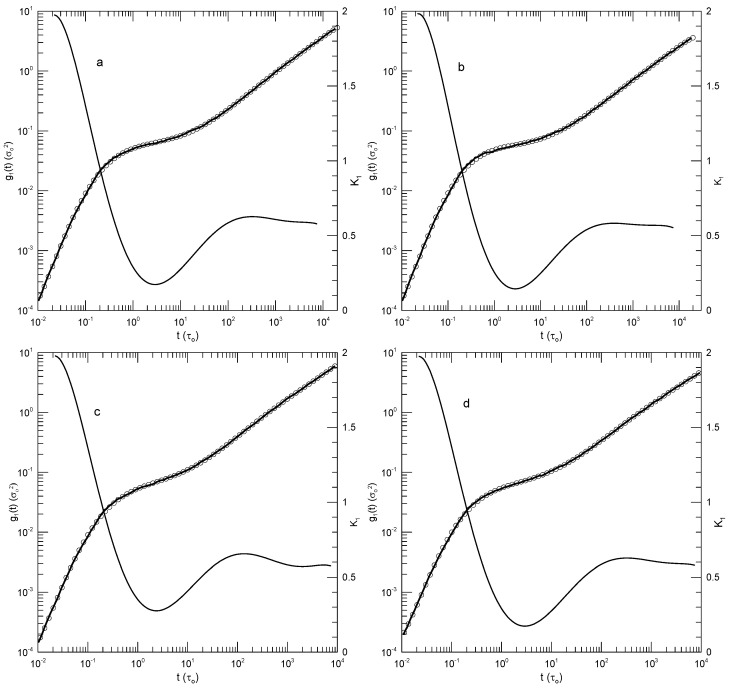
Mean-square bead displacements (thick lines) of B beads in (**a**,**b**) non-phase-separated mixtures, (**c**) the pure-B melt, and (**d**) in a phase-separated mixture, from Peng et al. [21], together with polynomial fits (circles) and first logarithmic derivatives $K_{1}$ (thin lines). The A chains had lengths of (**a**) 5, (**b**) 20, and (**d**) 50 beads.

Figure 9 shows a representative sample of Peng et al.’s determinations of the mean-square displacements of B beads in a pure-B melt and in A–B mixtures, both in phase-separated and phase-non-separated systems. The A beads, when present, were at a mole fraction of 0.1. The dependence of $g_{1}\left(t\right)$ on the chain length of the A beads was very weak.

These were rather short polymers. In each system, at the shortest times, $K_{1}$ approached 2.0. $K_{1}$ then declined rapidly to an inflection point, the minimum of $K_{1}$ being in the range 0.15–0.28 at times 2–2.5. $K_{1}$ then increased again to 0.55–0.59 in the four displayed systems. From the measurements, $g_{1}\left(t\right)$ in these systems showed shorter-time asymptotic behavior, an inflection point at intermediate times, and near-power-law behavior at the longest times studied. There were no indications of additional power-law regimes at intermediate times. Our finding of a long-time power-law regime is in quantitative agreement with the analysis of Peng et al. [21] who reported power laws with α∈ 0.56–0.58 at times $t>10^{3}$.

Peng et al. [21] also reported that the distribution function for displacements of the B beads was substantially non-Gaussian at intermediate times 1≤t≤1000. In the pure-B melt, $\alpha_{2}\left(t\right)$ at maximum was >0.4, while in the mixture, $\alpha_{2}\left(t\right)$ at maximum was >0.9, the maxima in the two systems being seen for 10<t<100. $\alpha_{2}\left(t\right)$ did not go to zero at the largest observed times. It follows from Doob’s theorem [8] that the forces on the individual beads did not have the uncorrelated Gaussian random distributions assumed by some polymer models.

Hsu and Kremer [22] examined static and dynamic properties of long-chain linear polymers with lengths of 500, 1000, or 2000 beads in simulated melts. Simulations each had 1000 chains in a periodic box, so simulated systems contained between 0.5 and 2 million beads, depending on the chain length. Polymer beads interacted with a truncated Lennard–Jones potential, a FENE potential representing chemical bonds, and a bond–bond bending potential. The authors determined the mean-square displacements $g_{1}\left(t\right)$ of individual beads in the center half of each chain, the mean-square displacements $g_{2}\left(t\right)$ of beads relative to the centers of mass of their respective chains, and the mean-square displacements $g_{3}\left(t\right)$ of the centers of mass of the chains. Hsu and Kremer inferred two estimates of $N_{e}$, viz., 26 or 32 beads, from which they found that the chains were nominally strongly entangled.

**Figure 10:**
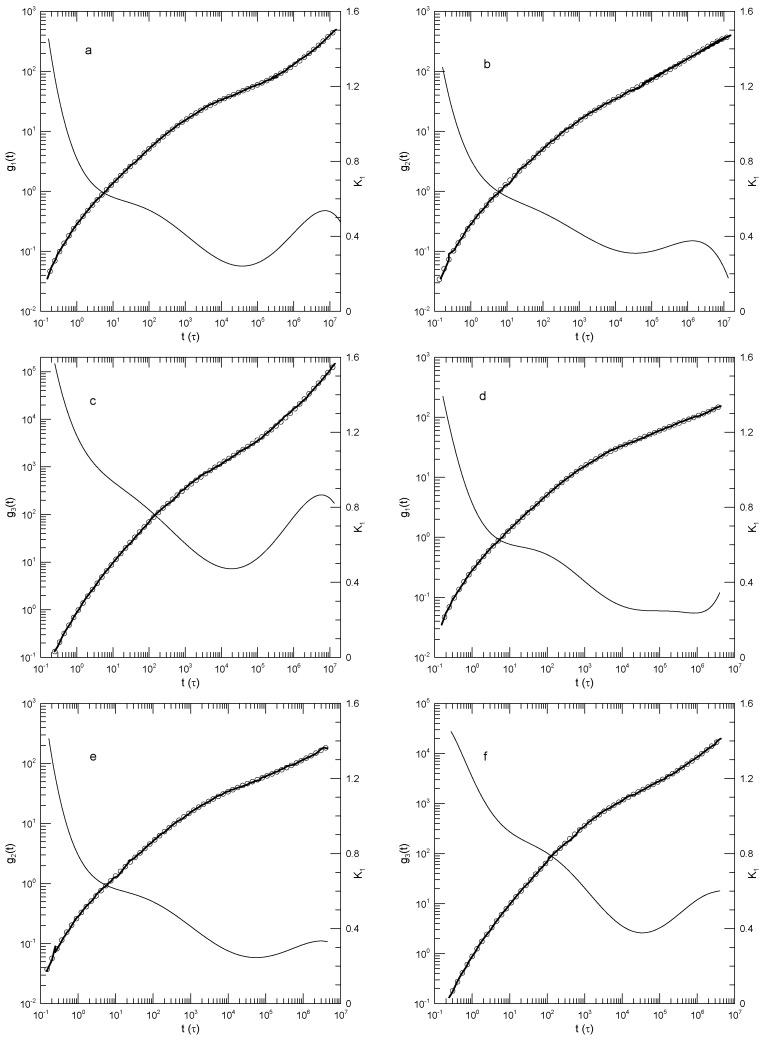
Hsu and Kremer’s [22] results for (**a**–**c**) 500-bead and (**d**–**f**) 2000-bead chains [22]. Graphs show mean-square motions of (**a**,**d**) individual beads, (**b**,**e**) bead motions relative to chain centers of mass, and (**c**,**f**) chain centers of mass. Heavy lines are the simulations, circles are polynomial fits, and thin solid lines represent first derivatives $K_{1}$.

Figure 10 shows their measurements of the mean-square displacements for the 500- and 2000-bead chains and our analysis of their measurements. Several features are noteworthy. First, the polynomial fits to the measurements visibly and accurately represented the reported mean-square displacements over a full range of reported times and displacements. Second, considering the first derivatives $K_{1}$ of the fitted curves, at early times, the g(t) all increased rapidly, so $K_{1}$ was close to 2.0. $K_{1}$ decreased rapidly until it reached times in the range 1–10, following which it decreased less rapidly until it reached a single minimum. Correspondingly, each g(t), while increasing, was concave downward until it reached an inflection point.

An interesting exception is seen in Figure 10d, showing the mean-square displacements of the central single beads of the 2000-bead chain. At long times, $K_{1}$ is in the near-constant range of 0.26–0.24, while the second derivative of the fitted curve becomes $K_{2}=0.0\pm 0.01$. That is, there is a region at times $2.7\cdot 10^{4}$–$1\cdot 10^{6}$ in which $K_{1}\approx 0.25$ is very nearly constant. Correspondingly, g(t) in this region follows a power-law with $g_{1}\left(t\right)∼t^{0.25\pm 0.01}$.

The slope of g(t) at an inflection point does not correspond to a power-law exponent, but it might nonetheless be of interest as reflecting a ghostly shadow of a power law almost but not quite buried under two adjoining non-power-law regimes. The slopes at the inflection points and the single power-law regime appear as Table 2.

The exception in Figure 10d, the revealed power-law behavior, has general significance for the analysis in this paper. Polynomials describe smooth curves, not (in general) straight lines. One might therefore be concerned that because we used polynomial fits, our fitting function might somehow have masked the presence of legitimate power-law behavior. As seen in this figure, a power-law dependence of g(t) on *t* was captured by our fitting process, when the dependence was present.

Hsu and Kremer interpreted their mean-square measurements by assuming that the reptation-scaling model predictions of power laws $g\left(t\right)∼t^{\alpha }$ and the model-predicted exponents were correct. They were able to identify times near which power-law curves were tangents to the g(t) results. Good agreement between the reptation-scaling power laws and the simulated curves was therefore found.

The results of Brodeck et al. [23] are of general interest for this paper, because Brodeck et al. ’s g(t) results did have power-law regimes that were revealed by our approach, thus demonstrating that our approach would find power-law behavior when present. Brodeck et al. reported simulations of polyethylene oxide/polymethylmethacrylate (PEO–PMMA) and simple bead–spring blends. PEO–PMMA is notable for its high degree of dynamical asymmetry, the glass temperatures of the two components being separated by 200 K. The PEO–PMMA simulations were based on the COMPASS force-field; the corresponding simulation cell contained equal numbers of PMMA and PEO chains, each chain containing 21 monomers.

**Figure 11:**
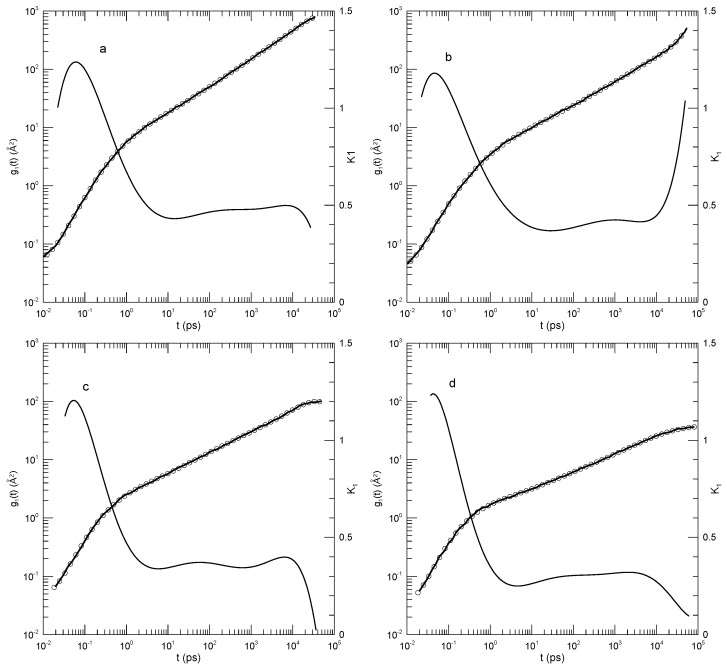
Mean-square displacements $g_{1}\left(t\right)$ of individual PEO hydrogen atoms in PEO–PMMA blend melts at temperatures (**a**) 500, (**b**) 400, (**c**) 350, and (**d**) 300 K, from Brodeck et al. [23]. Heavy lines indicate Brodeck et al. ’s results, circles are the eighth-order polynomial fits, and thin solid lines represent first logarithmic derivatives $K_{1}$.

**Figure 12:**
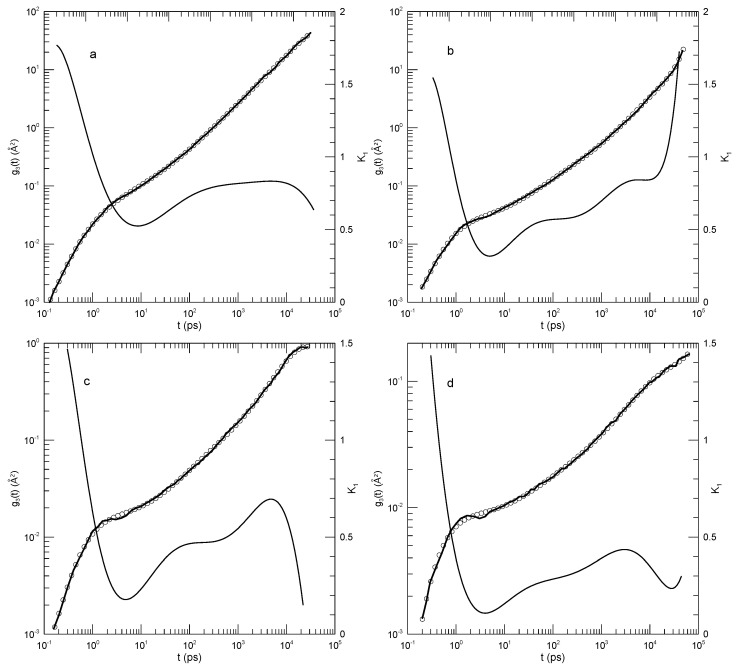
Mean-square displacements $g_{3}\left(t\right)$ of chain centers-of-mass of PEO molecules in PEO-PMMA blend melts at temperatures (**a**) 500, (**b**) 400, (**c**) 350, and (**d**) 300 K from Brodeck et al. [23]. Heavy lines indicate Brodeck et al.’s results, circles are the eighth-order polynomial fits, and thin solid lines represent first logarithmic derivatives $K_{1}$.

Figure 11 and Figure 12 show the mean-square displacements of single hydrogen atoms and of chain centers of mass, respectively, for the PEO chains in the PEO-PMMA mixtures. Simulations were performed at each of four temperatures and times 0.01–$10^{5}$ in natural units.

Figure 11 shows $g_{1}\left(t\right)$ for PEO in a PMMA melt at four temperatures. In each case, $g_{1}\left(t\right)$ clearly had a power-law region, as confirmed by our quantitative analysis. For the four Figure 11a–d being in order of decreasing temperature, $K_{1}$ took on values 0.48, 0.42, 0.36, and 0.31, respectively. For each $g_{1}\left(t\right)$, there was an extended region from $t\approx 10^{2}$ to *t* in the range 3000–8000 in which the second derivative satisfied $|K_{2}|\leq 0.01$. Between the shortest and longest times at which the power-law behavior was seen, $K_{1}$ was either constant or perhaps increased by 0.02 over the full time range.

In each figure, the ability of our method to capture minor features of $g_{1}\left(t\right)$, features that would not necessarily be apparent to the eye, is evident. As remarked by Brodeck et al., in all four studies of $g_{1}\left(t\right)$, at the longest times, $g_{1}\left(t\right)$ deviated from a power law. These deviations were captured by our fitting procedure. In Figure 11b, at long times, g(t) increases rapidly, while $K_{1}$ increases to unity. In Figure 11c,d, $g_{1}\left(t\right)$ appears to approach a plateau, so that $K_{1}\to 0$. Less obvious is the behavior of $g_{1}\left(t\right)$ at early times, times before the power-law regime is reached. In each case, at early times, $g_{1}\left(t\right)$ increases rapidly, with $K_{1}$ as large as 1.2. With increasing *t*, $K_{1}$ then rolls over until it reaches the slope associated with the power-law regime. However, in each Figure, before $K_{1}$ reaches its power-law value, it has a hitherto-unmentioned local minimum, so that $g_{1}\left(t\right)$ has an inflection point, and $K_{1}$ actually approaches its power-law behavior from values below its value in the power-law regime.

Brodeck et al. [23]. reported that the measured distribution functions for the displacements of the hydrogen atoms were not Gaussian in form. The non-Gaussianity parameter $\alpha_{2}\left(t\right)$ was small at early times but increased with increasing time.

Figure 12 shows $g_{3}\left(t\right)$ for PEO molecules in PMMA melts, these being the same systems as those described in Figure 11. Of these, as seen in Figure 12a, at 500 K, there was a somewhat narrow power-law region in which $K_{1}\approx 0.82$. Figure 12b–d show a fundamentally different behavior for $g_{3}\left(t\right)$, namely, $g_{3}\left(t\right)$ had an initial rapid increase, then a near-plateau and corresponding local minimum in $K_{1}\left(t\right)$, and finally a continuously increasing slope until a long-time behavior was reached. The local minimum in $K_{1}$ became deeper with decreasing *T*, namely, from $K_{1}=0.53$ at 500 K to $K_{1}=0.11$ at 300 K. The long-time limiting behaviors in Figure 12b–d were unique, namely, in the three figures, $g_{3}\left(t\right)$ at long times showed a progressive increase, a plateau, and an inflection point, respectively.

Brodeck et al. [23] continued their study by simulating a two-component bead–spring model of a polymer melt. The polymer A and B components differed in their interaction diameters, the slower A component having larger diameter beads. Studies were conducted at each of six temperatures between 0.4 and 1.5 in natural units. Their results were qualitatively the same as those they had already found in their PEO/PMMA simulations, confirming that the behaviors seen above were not peculiarities unique to PEO–PMMA blend melts.

Stephanou et al. [24] reported an extensive effort to apply Everaers et al.’s Primitive Path Analysis [25] approach to a discussion of the dynamics of polymer bead–spring models. Their approach was to reanalyze previously published simulations [26,27] of linear polyethylenes and linear polybutadienes. The primary effort was a consideration of the average rate at which a polymer molecule diffusively escaped from the hypothesized tube of the deGennes–Doi–Edwards [2] model of polymer dynamics. However, as part of the study, Stephanou et al. reported the atomistic mean-square displacement $g_{1}\left(t\right)$ of the center section of polyethylenes in a melt. They also reported for the same chains the mean displacement N(t) of primitive path segments perpendicular to a nominal primitive path.

**Figure 13:**
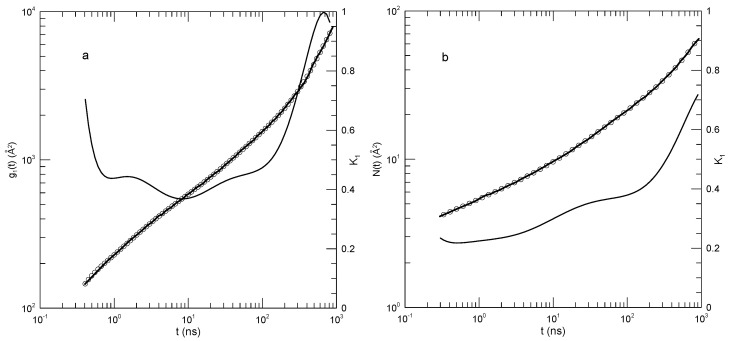
(**a**) Mean-square displacement $g_{1}\left(t\right)$ of the central beads of a chain and (**b**) mean displacement of individual primitive path segments perpendicular to the chain’s primitive path, based on Stephanou et al. [24]. Heavy lines are digitizations of Stephanou et al.’s data, circles are from the two eighth-order least-mean-square polynomial fits, and the thin solid line represents the first derivative $K_{1}$ of the polynomial fit.

Stephanou et al.’s [24] measurements of $g_{1}\left(t\right)$ and N(t) over times 0.4–900 appear in Figure 13 together with our polynomial fits to their results. Stephanou et al. asserted for $g_{1}\left(t\right)$ that *“Four distinct regimes are seen in the figure exactly as proposed by the reptation theory"*. We would describe $g_{1}\left(t\right)$ and N(t) differently, namely, to our eyes, $g_{1}\left(t\right)$ and N(t) both followed smooth, monotonically increasing curves. Our analysis thus did not find the four power-law regimes that Stephanou et al. saw in their data. Namely, for $g_{1}\left(t\right)$, we found that $K_{1}$ had one pronounced local minimum and correspondingly, an inflection point in $g_{1}\left(t\right)$ and one time domain of significant extent within which $K_{1}$ was constant, namely, $K_{1}\approx 0.5$ for times t≈1, corresponding to a single $t^{0.5}$ power-law regime at early times. Stephanou et al. ’s determinations of N(t) also found a smooth curve, whose slope $K_{1}$ increased monotonically with time. We did not find power-law behavior in N(t).

Likhtman et al. [28] reported a study of bead–spring polymers as described by the Kremer–Grest model. Beads had Lennard–Jones potential energies; springs were described by the FENE potential; springs were tuned to prevent chain crossing. Their primary interest was calculating the linear viscoelastic spectra of linear polymer melts, but they also calculated mean-square polymer displacements. Their measurements covered more than six orders of magnitude in time. We considered here their results for the mean-square displacement of the chain’s central monomers of chains containing 50, 100, 200, or 350 beads. Likhtman et al. [28] estimated that even their longest chains were only modestly entangled, with no more than seven entanglements per chain. They explored the temporal form of $g_{1}\left(t\right)$ by dividing their results for $g_{1}\left(t\right)$ by a time dependence $t^{1/2}$ identified with the Rouse model and separately with a time dependence $t^{1/4}$ associated with the reptation model and examined graphs of $g_{1}\left(t\right)/t^{1/2}$ and $g_{1}\left(t\right)/t^{1/4}$. They found that plots of these functions were “…very rich and full of features”. Here, we confined ourselves to the $g_{1}\left(t\right)$ of the central monomer, of which they said “…we find it very difficult to identify clear power-laws in this plot …”, a conclusion with which we agree.

**Figure 14:**
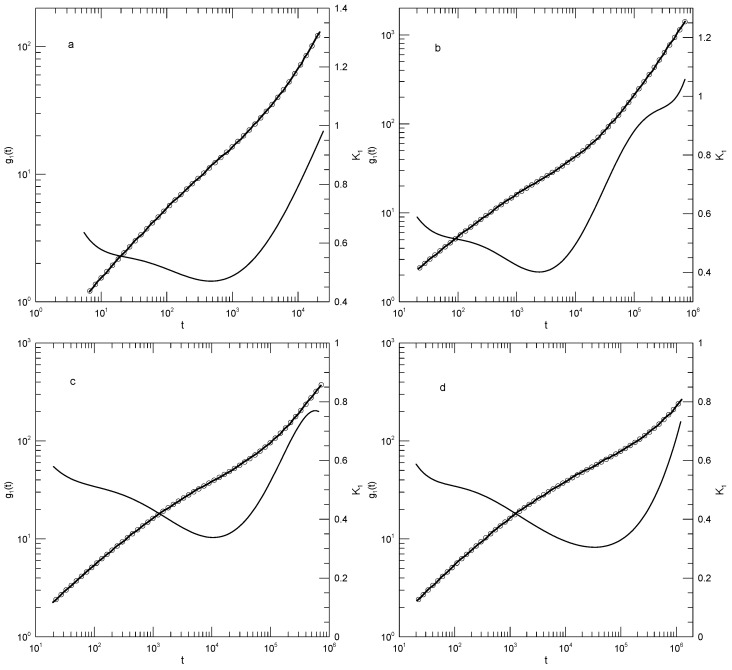
Mean-square displacements of central beads of (**a**) 50-, (**b**) 100-, (**c**) 200-, and (**d**) 350-bead simulated Grest–Kremer polymer melts, based on Likhtman et al. [28]. Here, heavy lines are digitizations of Likhtman et al. ’s data, circles represent eighth-order polynomial fits, and thin solid lines show the first derivatives of the two polynomial fits.

Figure 14 shows their determinations of $g_{1}\left(t\right)$, our polynomial fits, and its logarithmic derivative $K_{1}$ for each polymer size. Qualitatively, the four figures are very similar. In each case, the polynomial fit (circles) accurately describes the measurements. The first derivative curves are all qualitatively the same. $K_{1}$ slowly decreases to a local minimum, and then, at longer times, increases more rapidly. The minimum value of $K_{1}$ decreases with the increasing polymer length, namely, to 0.47, 0.40, 0.34, and 0.30, respectively. These are the only minima, the only regions where the slope of $K_{1}$ changes sign from negative to positive. Correspondingly, each $g_{1}\left(t\right)$ curve has a single inflection point. The time at which the inflection point is found increases from ≈470 to ≈37,000 with the increasing chain length. There are other regions, notably at times 10≤t≤100, where $K_{1}$ is relatively slow-changing, but in all those regions the time derivative $K_{2}$ of $K_{1}$ keeps a single sign at all points.

Zhou and Larson [29] calculated $g_{2}\left(t\right)$, the mean-square monomer motion relative to the chain center of mass, for a bead–spring polymer model incorporating a Lennard–Jones potential, a FENE potential for each bead–bead bond, and a three-bead bending potential. Their polymer chains contained either 150 or 300 beads; chains were estimated to be subject to an average of 6.5 or 13 entanglements, respectively. Bead motions were calculated using molecular dynamics; the constant-temperature condition was maintained by applying to each bead a random force and a velocity-determined friction force. The calculations of $g_{2}\left(t\right)$ were part of a larger paper in which the relaxation modulus G(t) and the potential energy of the confining tube, as determined from the excursion distance of each bead from its primitive path, were calculated.

**Figure 15:**
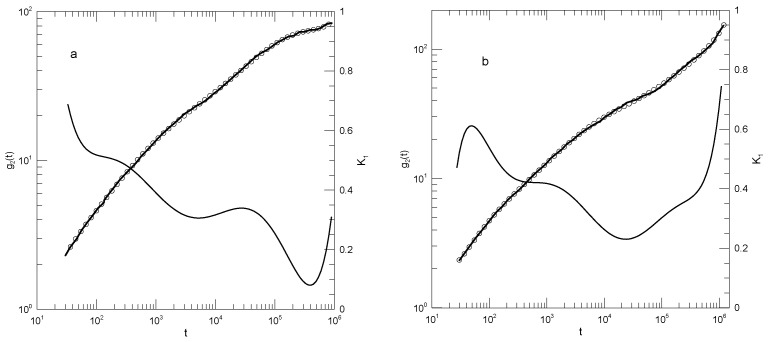
Mean-square displacement $g_{2}\left(t\right)$ of a polymer’s beads relative to its center of mass, for bead–spring polymers having lengths (**a**) 150 and (**b**) 300 beads, based on Zhou and Larson [29]. Heavy lines are digitizations of Zhou and Larson’s data, circles represent eighth-order polynomial fits, and thin solid lines show the first derivative of each polynomial fit.

Figure 15 shows $g_{2}\left(t\right)$ and its first logarithmic derivative for the two polymers studied by Zhou and Larson. $K_{1}$ had the same dominant features for the N=150 and N=300 polymers, namely, at short times, $K_{1}$ was relatively large (0.69 or 0.61, respectively), as time advanced, $K_{1}$ fell and reached a minimum (0.08 or 0.23, respectively), and at longer times, $K_{1}$ increased. For the N=150 polymer, near time t=5000, $K_{1}$ had a local minimum and was then nearly constant, with $K_{1}\in$ (0.31–0.34). There were several other narrow regions where $K_{1}$ could be said to be nearly constant, notably for the N=300 polymer near times 400–900, with $K_{1}\approx 0.41$.

Zhou and Larson compared their results with a theoretical model that predicted power-law behavior. They fit sections of $g_{2}\left(t\right)$ to $t^{1/4}$ and $t^{1/2}$ power laws, finding regions where the measured $g_{2}\left(t\right)$ was not transparently inconsistent with these power laws. However, on a log–log plot, power laws were straight lines, while as seen above, $g_{2}\left(t\right)$ for each polymer followed a smooth curve with a continuously changing slope. From our analysis, their interpretation of $g_{2}\left(t\right)$ corresponds to a valid best fit of their results to a particular theoretical model, even though the results might be better described otherwise.

Moreno and Colmenero [30] reported simulations of a binary blend of two species of bead–spring polymers. Each polymer molecule contained ten beads. Beads interacted via an $r^{-12}$ and an $r^{2}$ potential with a cutoff distance. Springs were represented with a FENE potential. The A polymer beads were 60% larger than the B polymer beads and thus had appreciably slower dynamics. Mean-square displacements were reported for the pure B polymer, and for the B polymer in a blend with a B mole fraction $x_{B}=0.3$, at temperatures between 0.4 and 1.5 in natural units. Periodic boxes contained between 250 and 600 chains.

**Figure 16:**
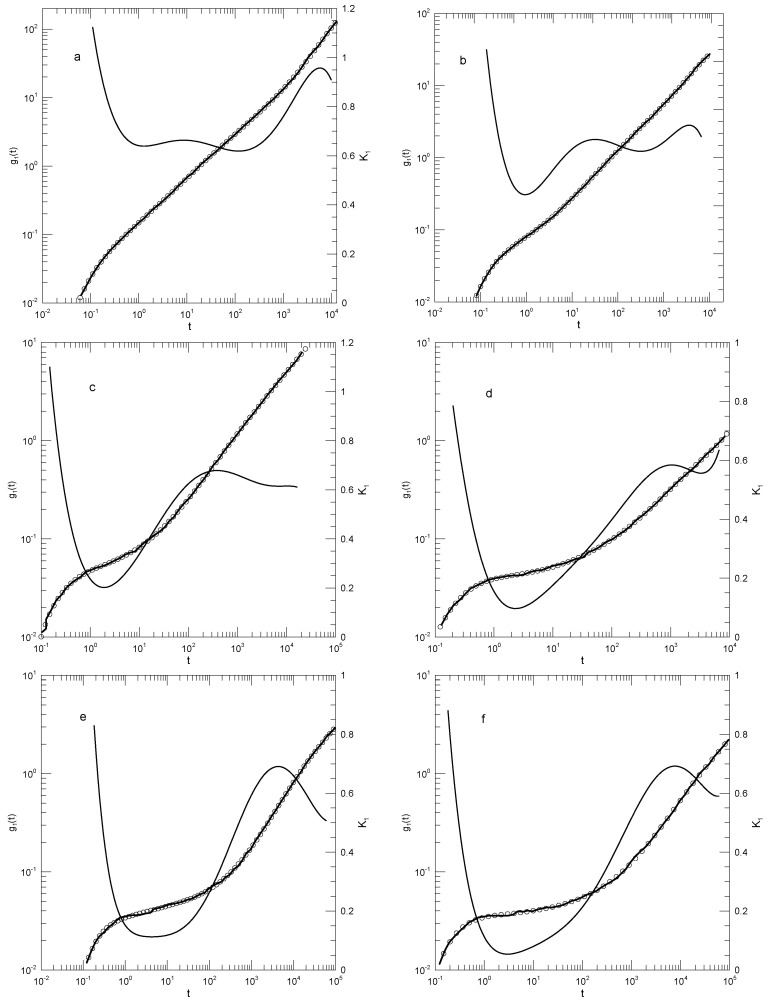
(**a**) Mean-square displacement $g_{1}\left(t\right)$ of individual beads of a pure B melt from data from Morenoand Colmenero citemeansqmoreno2006a at temperatures (**a**) 1.5, (**b**) 1.0, (**c**) 0.70, (**d**) 0.60, (**e**) 0.57, and (**f**) 0.55. Heavy lines are digitizations of Moreno et al.’s data, circles represent eighth-order polynomial fits, and thin solid lines are first derivatives $K_{1}$ of the polynomial fits.

Our fits to Moreno and Colmenero’s [30] data on the pure B polymer melts, as seen in Figure 16, showed systematic changes with decreasing temperature. At the largest temperature studied, T=1.5, $g_{1}\left(t\right)$, as seen in Figure 16a, had an initial rapid increase and then climbed approximately as a power law in *t*, the exponent being 0.64±0.02. At the longest times reported, $g_{1}\left(t\right)$ increased more rapidly, the exponent of an inferred power-law description reaching 0.95 at long times. At lower temperatures, there were qualitative changes in the form of $g_{1}\left(t\right)$. At temperatures T=1.0 and below, the slope $K_{1}$ decreased to a local minimum and then increased again. At the local minimum, for T=1.0, $K_{1}=0.45$; with decreasing temperature, $K_{1}$ at the minimum decreased monotonically. At times longer than the minimum in $K_{1}$, at larger temperatures, $K_{1}\approx 2/3$ was nearly constant. At lower temperatures and times beyond its minimum, $K_{1}$ was not a constant; it instead increased monotonically with increasing time until a maximum was reached.

Moreno and Colmenero [30] ’s description of their results on the pure B polymer was qualitatively the same as ours. With decreasing temperature, at shorter times, “...a plateau developed...”, that is, there was a region of small slope that was increasingly manifested. At longer times, we found an area with a slope close to 0.65, as already described by Moreno et al., though at the lowest temperatures, the increase in slope from the plateau area was extended, so that the region with $K_{1}\approx 2/3$ was reduced to a local maximum in $K_{1}$ corresponding to an inflection point in $g_{1}\left(t\right)$. Around that local maximum, a slope close to 2/3 that could be approximated as a naked-eye near-straight line was seen.

**Figure 17:**
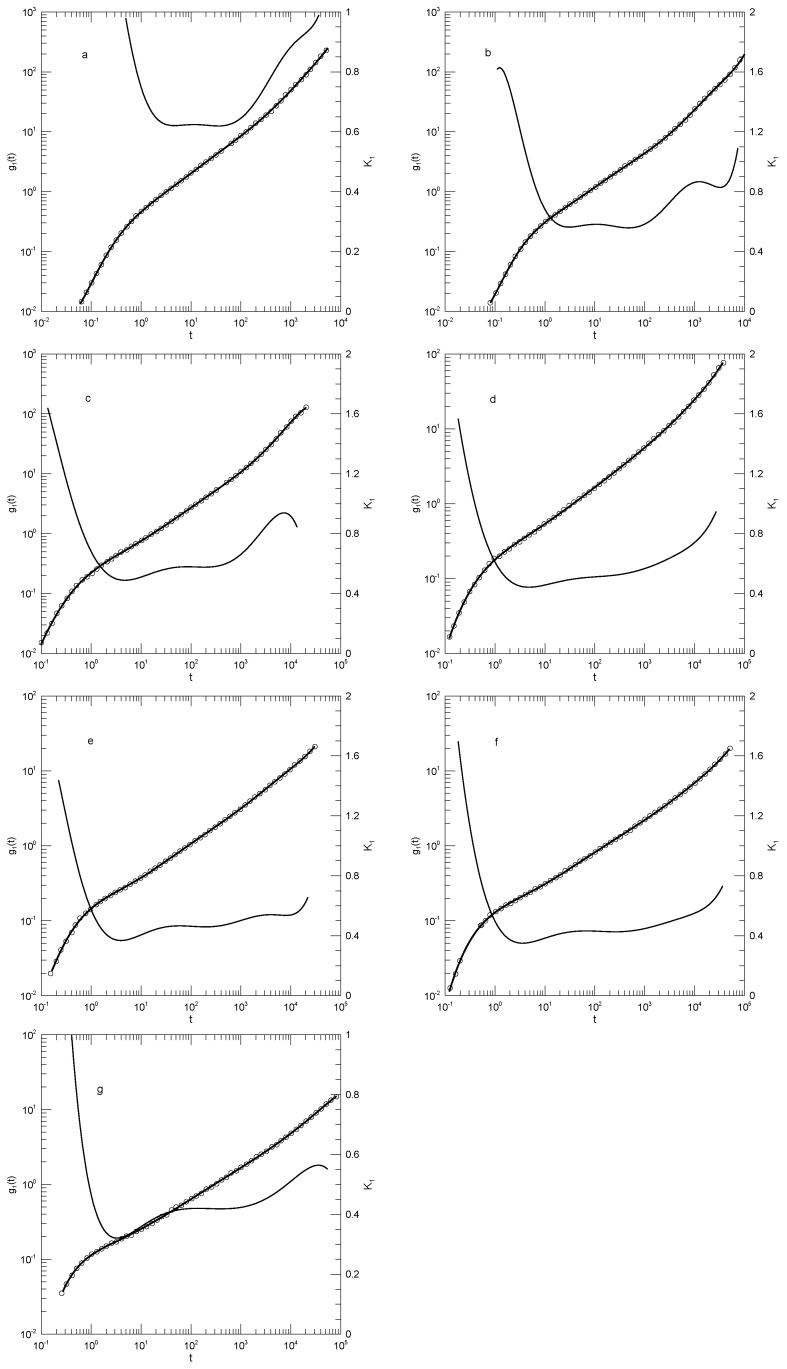
(**a**) Mean-square displacement $g_{1}\left(t\right)$ of the B beads of a melt of an A–B polymer blend with a *B* mole fraction $x_{B}=0.3$, based on Moreno et al. [30] at temperatures (**a**) 1.5, (**b**) 1.0, (**c**) 0.75, and (**d**) 0.60. Heavy lines represent Moreno et al.’s data, circles are eighth-order polynomial fits, and thin solid lines are first derivatives $K_{1}$ of the fits. Mean-square displacement $g_{1}\left(t\right)$ of the B beads in an A–B polymer blend melt with $x_{B}=0.3$, based on Moreno et al. [30] at temperatures (**e**) T=0.50, (**f**) T=0.45, and (**g**) T=0.40. Heavy line show Moreno et al.’s data, circles represent the eighth-order polynomial fits, and thin lines are the first derivatives $K_{1}$ of the polynomials.

The time dependence of $g_{1}\left(t\right)$ in the $x_{B}=0.3$ blend, as seen in Figure 17, had a somewhat simpler structure than did $g_{1}\left(t\right)$ in the pure melt. After an initial increase, for temperatures T≥1.0, $K_{1}$ became nearly constant, changing almost not at all. In this temperature range, for times 3≤t≤100, $g_{1}\left(t\right)$ did follow a power law, with exponents 0.63 or 0.56, respectively. At temperatures T≤1.0 and a broad range of times, we found that $K_{1}$ increased by perhaps 0.1 or 0.2 with increasing time, but it was clearly not a constant. At lower temperatures, $g_{1}\left(t\right)$ therefore did not follow a power law in time.

Moreno et al.’s [30] description of their results on the $x_{B}=0.3$ blend was broadly consistent with what is seen in Figure 17. There was an early ballistic regime followed by a rollover to a much shallower slope. Moreno et al. presented straight-line descriptions of $g_{1}\left(t\right)$ in the shallower regime with slopes decreasing from 0.635 to 0.415 as temperature was reduced. Our results were quantitatively fairly close to theirs, except that we found that not-quite-straight lines having small curvatures gave a more accurate description of $g_{1}\left(t\right)$ in that regime.

Padding and Briels [31,32,33,34] reported simulations of linear polyethylene melts with chain lengths extending from $C_{80}$ to $C_{1000}$, including calculations of $g_{1}\left(t\right)$, the dynamic structure factor S(q,t), and Rouse mode amplitudes and time correlation functions. They used molecular dynamics and a united atom model with harmonic bead–bead stretch and bend potential energies, a dihedral angle potential, and van der Waals forces between atoms that were not adjoining atoms along each polymer chain. For some purposes, united atoms were aggregated into *blobs*, typically of 20 atoms. Blob dynamics were inferred from united-atom dynamics; the purpose of the blobs was to allow the simulation of much longer polymers than would otherwise be possible with then-available computing power. Of relevance here, Padding and Briels studied the time-dependent mean-square displacements of individual atoms $g_{1}\left(t\right)$, chain centers of mass $g_{3}\left(t\right)$, and individual blobs $g_{\mathrm{bl}}\left(t\right)$. Other dynamic properties were also considered but are outside the scope of this paper.

**Figure 18:**
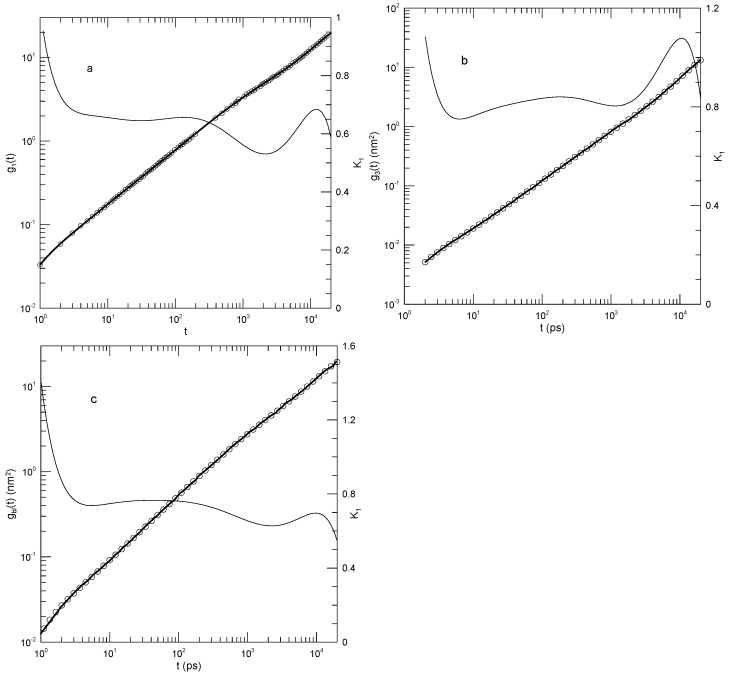
Mean-squaredisplacements of (**a**) individual atoms, (**b**) chain centers of mass, and (**c**) individual blobs $g_{\mathrm{bl}}\left(t\right)$ for a simulated T=450 K melt of *n*-C_120_H_242_. Heavy lines indicate Padding and Briel’s data [31], circles represent eighth-order polynomial fits, and thin solid lines represent first derivatives $K_{1}$ of the polynomial fits.

Their first paper [31] considered simulations of an *n*-C_120_H_242_ melt. Their results for $g_{1}\left(t\right)$, $g_{3}\left(t\right)$, and $g_{\mathrm{bl}}\left(t\right)$ appear in Figure 18. This figure is noteworthy for identifying a temporal region in which the mean-square displacement $g_{1}\left(t\right)$ clearly increases as a power law in time, namely, for 6≤t≤220, $g_{1}\left(t\right)∼t^{\alpha }$ for α≈0.66. At larger *t*, α first decreases to ≈0.53 and then increases to ≈0.68. In contrast, $g_{3}\left(t\right)$ only approaches power-law behavior, namely, for the center-of-mass motion, $K_{1}$ drops rapidly to 0.75, then over close to two decades in time increases to 0.84, and finally increases to 1.0 or a bit larger. The behavior of $g_{\mathrm{bl}}\left(t\right)$ is qualitatively similar to that of $g_{1}\left(t\right)$, namely, after an initial rapid decrease in $K_{1}$, there is an extended region in which α≈0.78 is encountered.

In a further paper, Padding and Briels [33] reported mean-square displacements of blobs—coarse-grained groups of 20 adjoining monomers—in simulated melts of linear polyethylene. Coarse-graining introduces complications. Most of the mechanical variables in the monomers of each blob must become bath variables, in the sense of the Mori–Zwanzig formalism, so that the blob equations of motion gain frictional and random thermal forces. The blob–blob potential energy, determined as the blob–blob potential of average force from a united-atom simulation of the same system, is soft, so an alternative procedure was introduced to create the chain’s non-crossing constraint.

**Figure 19:**
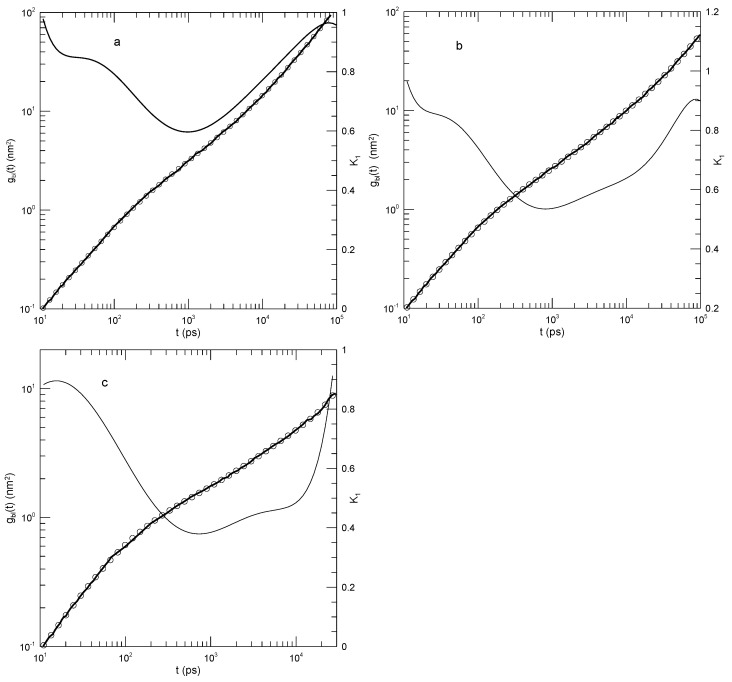
Mean-square displacements $g_{\mathrm{bl}}\left(t\right)$ of blobs in polyethylene melts containing (**a**) 4, (**b**) 6, or (**c**) 50 blobs (and, correspondingly, (**a**) 80, (**b**) 120, or (**c**) 1000 monomers). Heavy lines indicate Padding and Briel’s [33] data, circles show the eighth-order polynomial fits, and thin solid lines represent $K_{1}$, the first derivative of the polynomial fits.

Figure 19 shows their results for $g_{\mathrm{bl}}\left(t\right)$ for the 4-, 6-, and 50-blob chains. Their original figure (Figure 13 in their paper) also showed results for 10- and 20-bead polymer melts, but we encountered a limit of our procedure, namely, if the data points were sufficiently overlapped, it became impossible to digitize the graph reporting the data. Having said that, $g_{\mathrm{bl}}\left(t\right)$ curves for the three chain lengths were actually rather similar. The logarithmic derivative $K_{1}$ for each of the three $g_{\mathrm{bl}}\left(t\right)$ curves began at short times with a value close to 1.0, decreased to a minimum at times near t=1000, and then increased monotonically to a value close to 0.9. $K_{1}$ at the minimum was 0.60, 0.53, or 0.38, respectively, for the three chain lengths. Corresponding to the minimum in $K_{1}$, $g_{\mathrm{bl}}\left(t\right)$ had an inflection point. Except perhaps for the four-blob chains at early times (t<60), there were clearly no power-law regions at any time for any chain length. At times beyond the inflection point, $K_{1}\left(t\right)$ of the two longer chains showed some additional structure, namely, a region where it increased relatively slowly with increasing time.

In presenting their results, Padding and Briels [33] added to their log–log plot four straight lines to guide the eye, straight lines corresponding to power-law regimes, the labeled slopes being 1, 0.5, 0.4 (for the 50-blob chain), and 1. We found that the initial and terminal slopes were in reasonable agreement with Padding and Briels, namely, $K_{1}$ approached one. Where Padding and Briels suggested a power-law region with a slope of 0.4, we identified an inflection point whose minimum slope was $K_{1}=0.38$. The suggested $K_{1}=0.5$ line was in a region where there were few data points, and in which we found a slope that changed continuously, but their suggested line would be a tangent at some $10^{2}\leq t\leq 10^{3}$.

## 4. Discussion

This review considered simulations of polymer melts that determined polymer mean-square displacements g(t). The time dependence of g(t) is of specific interest because widely used theoretical models have as a core prediction that g(t) has the power-law behavior $g\left(t\right)∼t^{\alpha }$ in various time regimes. Equations (5)–(12) showed the predicted α in these regimes. Historically, a particular focus of simulations has been a search for the α=1/4 exponent of Equation (10).

We applied a new mathematical approach to test these predictions. As described in Section 2, for each simulated system, we determined the (time-dependent) logarithmic derivative $K_{1}$ of g(t). At each *t*, $K_{1}$ from our approach gave an effective local value for α. In any of the hypothetical power-law regimes, $K_{1}$ would be a constant over an appreciable range of times, as revealed by the second derivative $K_{2}$ approaching $K_{2}\approx 0$. With very few exceptions, such a phenomenon—a constancy of $K_{1}$—was not found. In almost all cases, we found that the power-law behavior of g(t) was not present. Instead, $K_{1}$ consistently showed a single more-or-less deep minimum, corresponding to a saddle point in the time dependence of g(t). Efforts to extract power-law exponents α from simulations of g(t) have largely been unsuccessful. Our analysis indicated that the efforts to extract a power law failed because the hypothesized power-law behavior was not there to begin with. Theoretical models that predict that power-law behavior is important are rejected by our analysis of previously published simulations.

We noted that in several cases, the final few moments of a measurement of g(t) deviated from what might have been expected to happen given the behavior of g(t) at slightly earlier times. Our fitting process replicated this behavior, leading to possible artifacts in $K_{1}\left(t\right)$ at the longest times studied, the artifacts being rapid changes in $K_{1}\left(t\right)$ at the largest *t* studied. We noted this behavior without expressing an opinion as to whether it was real or was a computational issue of no real consequence.

With specific regard to the deGennes–Doi–Edwards model and related scaling models, an obvious objection to our conclusion is that, even with modern computational techniques, simulations can only examine relatively short polymers, polymers that are not heavily entangled. Indeed, the longest polymers examined here had only 2000 beads, perhaps corresponding to as many as forty entanglements. The deGennes–Doi–Edwards description does not actually indicate how many entanglements per chain are needed for the tube-reptation model to be applicable, so this number of entanglements might be inadequate for the model to apply. There are also literature discussions as to what an entanglement actually is. A critic might therefore propose that the chains considered here were all unentangled. However, as seen in Equations (5)–(12), the deGennes–Doi–Edwards description also predicts the behavior of short chains, where there are few or no entanglements, and the behavior of long chains at short times, times too early for the hypothesized chain entanglements to affect polymer motion. These short-chain and short-time predictions were tested by our analysis. The predictions were incorrect; there was almost never temporal power-law behavior. When there was power-law behavior, the exponent did not have any of the predicted values.

It might be proposed that the predicted power-law regimes are separated by transition regimes in which power-law behavior is absent. It could be proposed that the transition regimes between power-law regimes are broad. Indeed, the latter proposal could be said to be supported by our analysis, in the sense that the hypothesized transition regimes could have completely swallowed up any hypothesized regions where power-law activity was found, reducing the power-law behavior to the slope at an inflection point. In that case, however, attempts to calculate hypothesized power-law exponents are pursuing a theoretical *Fata Morgana*. A satisfactory theory of polymer melt dynamics would then need to focus on hypothesized broad transition regimes, not the hypothesized regimes where power laws are encountered.

With specific regard to the Rouse model, there have long been indications that the Rouse model might not be appropriate for polymer molecules in a melt [35,36]. It would then be unsurprising that $g_{1}\left(t\right)$ and $g_{3}\left(t\right)$ of short polymers do not have the properties predicted in Equations (5)–(8). These equations describe polymers that move in accordance with the Rouse model. Our recent extensive review of simulations [12] that determined Rouse amplitudes $X_{p}\left(t\right)$ and their time correlation functions $\langle X_{p}\left(0\right)X_{q}\left(t\right)\rangle$ for polymers in simulated melts found none of the properties associated with a Rouse-model polymer. In particular:

(i) Mean-square Rouse mode amplitudes $\langle {\left(X_{p}\left(0\right)\right)}^{2}\rangle$ did not scale as predicted by the Rouse model;

(ii) Rouse-mode time correlation functions $\langle X_{p}\left(t\right)X_{p}\left(0\right)\rangle$ did not decay with time as the exponentials predicted by the Rouse model;

(iii) Polymer bead displacements were not described by the independent Gaussian random processes that are assumed by the Rouse model;

(iv) Rouse mode amplitudes were not always independent, i.e., it was not always true that $\langle X_{p}\left(0\right)X_{q}\left(t\right)\rangle =0$ for p≠q, as required by the Rouse model.

Proposals that polymer motions in the melt are described by the Rouse model, perhaps only at short times, are therefore systematically inconsistent with extensive published simulations. This result should not be surprising, because the underlying Hamiltonian (properly, the underlying Rayleigh dissipation function) for short polymer chains in their melt is not the function assigned by Rouse. Rouse assumed that the forces on the beads of a polymer could be modeled as a series of independent Gaussian random processes, one for each bead. This assumption was checked simulatively [12]. It was false. The displacement distribution function P(Δr,t) for the individual beads, the probability of finding a displacement Δr during a time *t*, was in general not a Gaussian; instead, the non-Gaussianity parameter $\alpha_{2}\left(t\right)$ for the polymer bead displacements was found to be non-zero and time-dependent.

We conclude that computer simulations serve to reject major core assumptions of the deGennes–Doi–Edwards models, and some other models, of a polymer melt, these being the Rouse-related predictions, the $g\left(t\right)∼t^{\alpha }$ prediction, and in consequence, predictions following from those predictions. There is then a need for a new approach to modeling polymer dynamics in the melt, for example, an approach based on the Altenberger–Dahler Positive-Function Renormalization Group [37,38].

## Figures and Tables

**Table 1:** Value of the logarithmic derivative $K_{1}$ at the inflection point, for different polymer lengths *N*, as determined from $g_{1}\left(t\right)$ or $g_{3}\left(t\right)$ as measured by Behbahani and Schmid [13].

*N*	$g_{1}\left(t\right)$	$g_{3}\left(t\right)$
10	0.65	0.87
30	0.59	0.84
50	0.56	0.82
100	0.52	0.78
150	0.47	0.74
200	0.44	0.69
400	0.38	0.62
1000	0.31	0.50

**Table 2:** Slopes at inflection points in the g(t) results of Hsu and Kremer [22]. * power-law regime.

Function	Figure	*N*	Slope
$g_{1}\left(t\right)$	Figure 10a	500	0.24
$g_{2}\left(t\right)$	Figure 10b	500	0.31
$g_{3}\left(t\right)$	Figure 10c	500	0.47
$g_{1}\left(t\right)$	Figure 10d	2000	0.25±0.01 *
$g_{2}\left(t\right)$	Figure 10e	2000	0.25
$g_{3}\left(t\right)$	Figure 10f	2000	0.38

## Data Availability

Data are contained within the article.

## Supplementary Materials

The following supporting information can be downloaded at: https://www.mdpi.com/article/10.3390/polym17091193/s1. The supporting information shows a full-page version of each graph in the text.
