# Supplementary material for: Review: Mean-Square Displacements of Simulated Polymers
Source: Polymers (Basel). 2025 Apr 27;17(9):1193. doi: 10.3390/polym17091193 (PMC12073395; doi:10.3390/polym17091193)
Supplement: Supplementary file 1 [file polymers-17-01193-s001.zip › polymers-3569370-supplementary.pdf]

Supplemental Material for
Review: Mean-Square Displacements of Simulated Polymers

George D. J. Phillies

Department of Physics, Worcester Polytechnic Institute, Worcester, MA 01609, USA

Abstract

The Supplemental Material here is a complete set of full-size figures, corresponding to the figures in the text, but on a scale permitting detailed examination, especially of our claim that there is good agreement between the simulational results and our polynomial fits.

* phillies@wpi.edu

Figure S-1a. Mean-square bead displacements $g_1(t)$ (thick line) of melts of 10-bead Kremer-Grest bead-spring chains, based on simulations of Behbahani and Schmid[1], together with a fit to an eighth-order polynomial (circles) and the corresponding first logarithmic derivative K_1 (thin line).

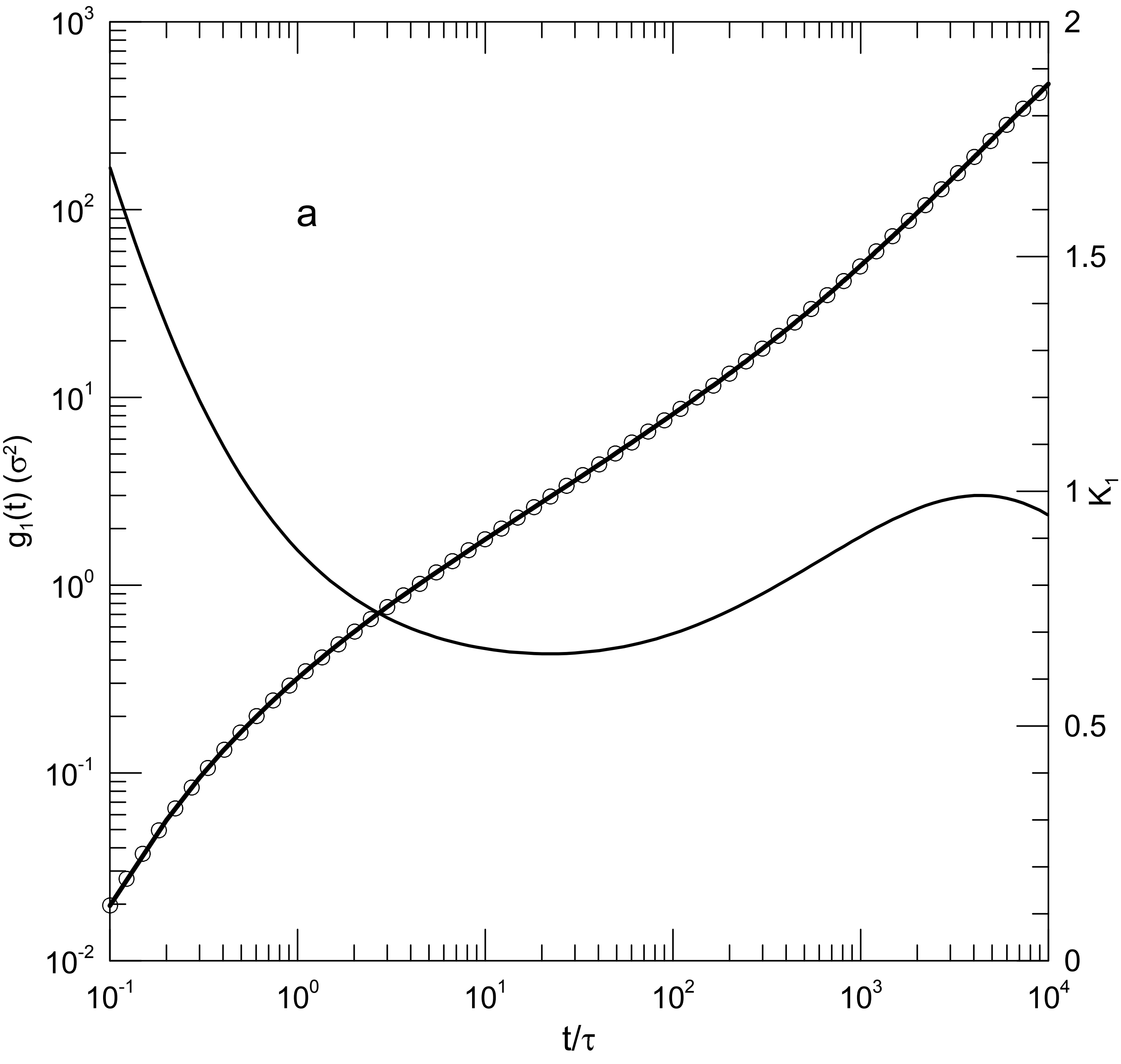

Figure S-1b. Mean-square bead displacements $g_1(t)$ (thick line) of melts of 30-bead Kremer-Grest bead-spring chains, based on simulations of Behbahani and Schmid[1], together with a fit to an eighth-order polynomial (circles) and the corresponding first logarithmic derivative K_1 (thin line).

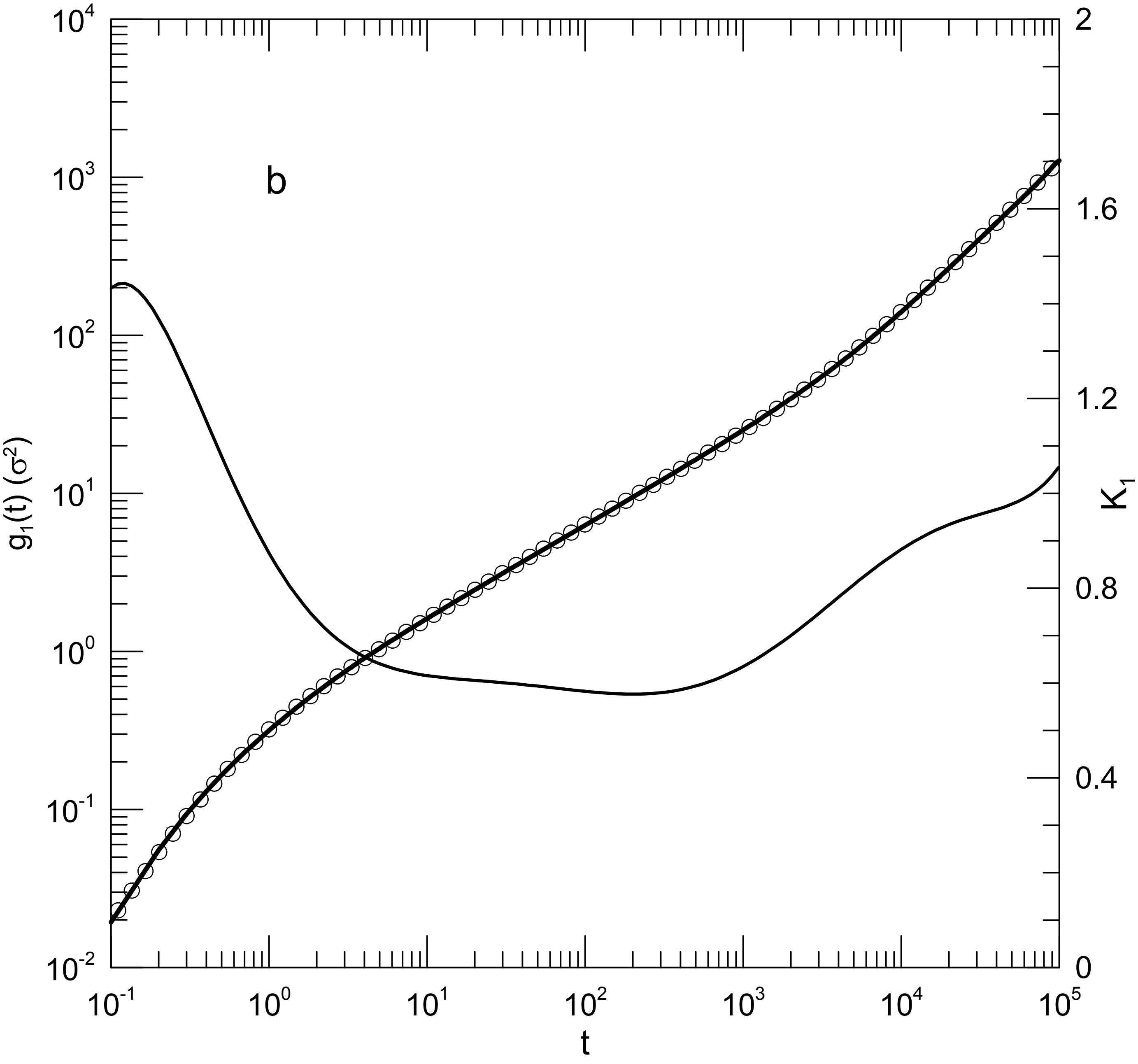

Figure S-1c. Mean-square bead displacements $g_1(t)$ (thick line) of melts of 50-bead Kremer-Grest bead-spring chains, based on simulations of Behbahani and Schmid[1], together with fits to eighth-order polynomials (circles) and the corresponding first logarithmic derivatives K_1 (thin line).

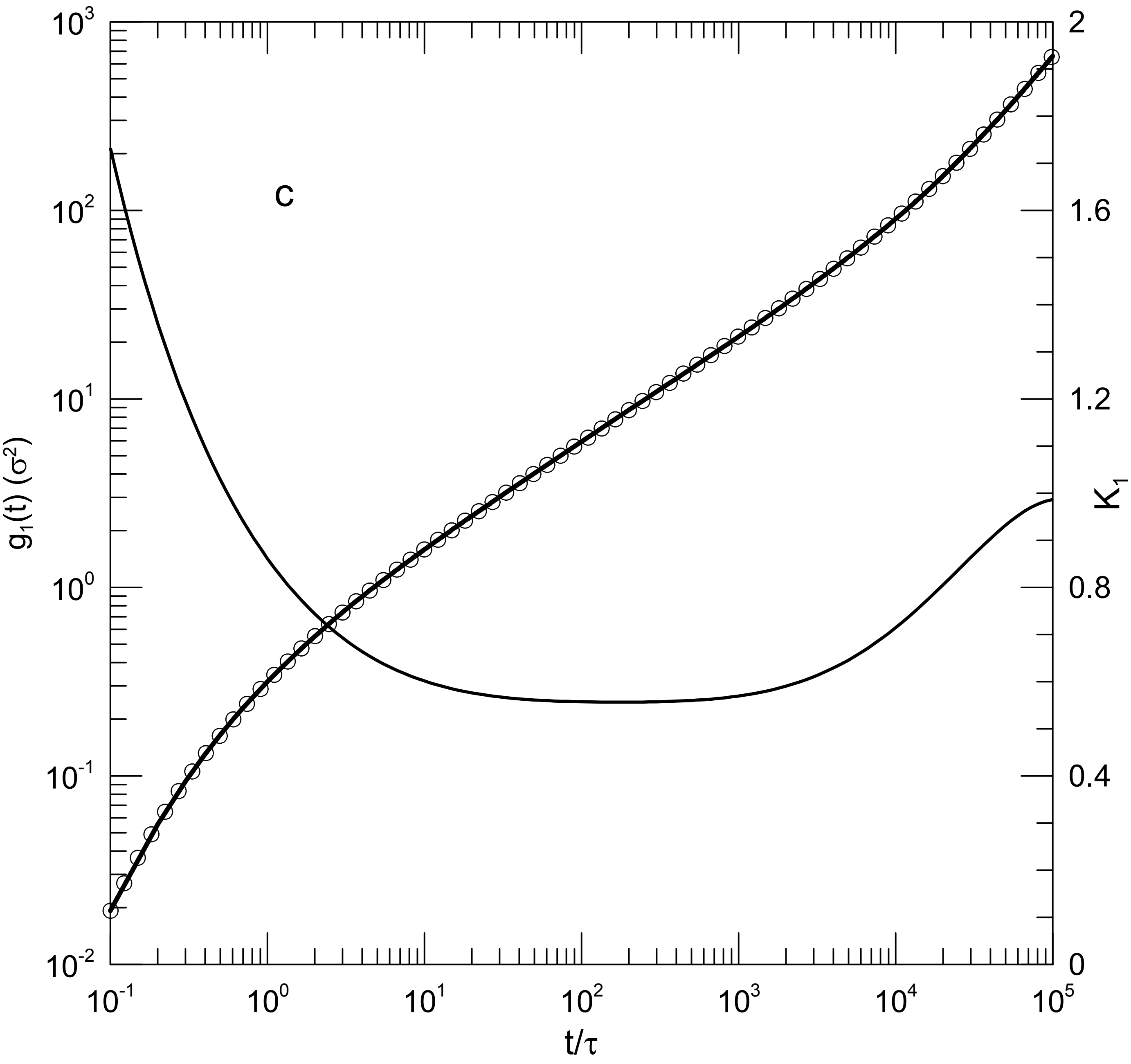

Figure S-1d.

Mean-square bead displacements $g_1(t)$ (thick line) of melts of 100-bead Kremer-Grest bead-spring chains, based on simulations of Behbahani and Schmid[1], together with fits to eighth-order polynomials (circles) and the corresponding first logarithmic derivatives K_1 (thin line).

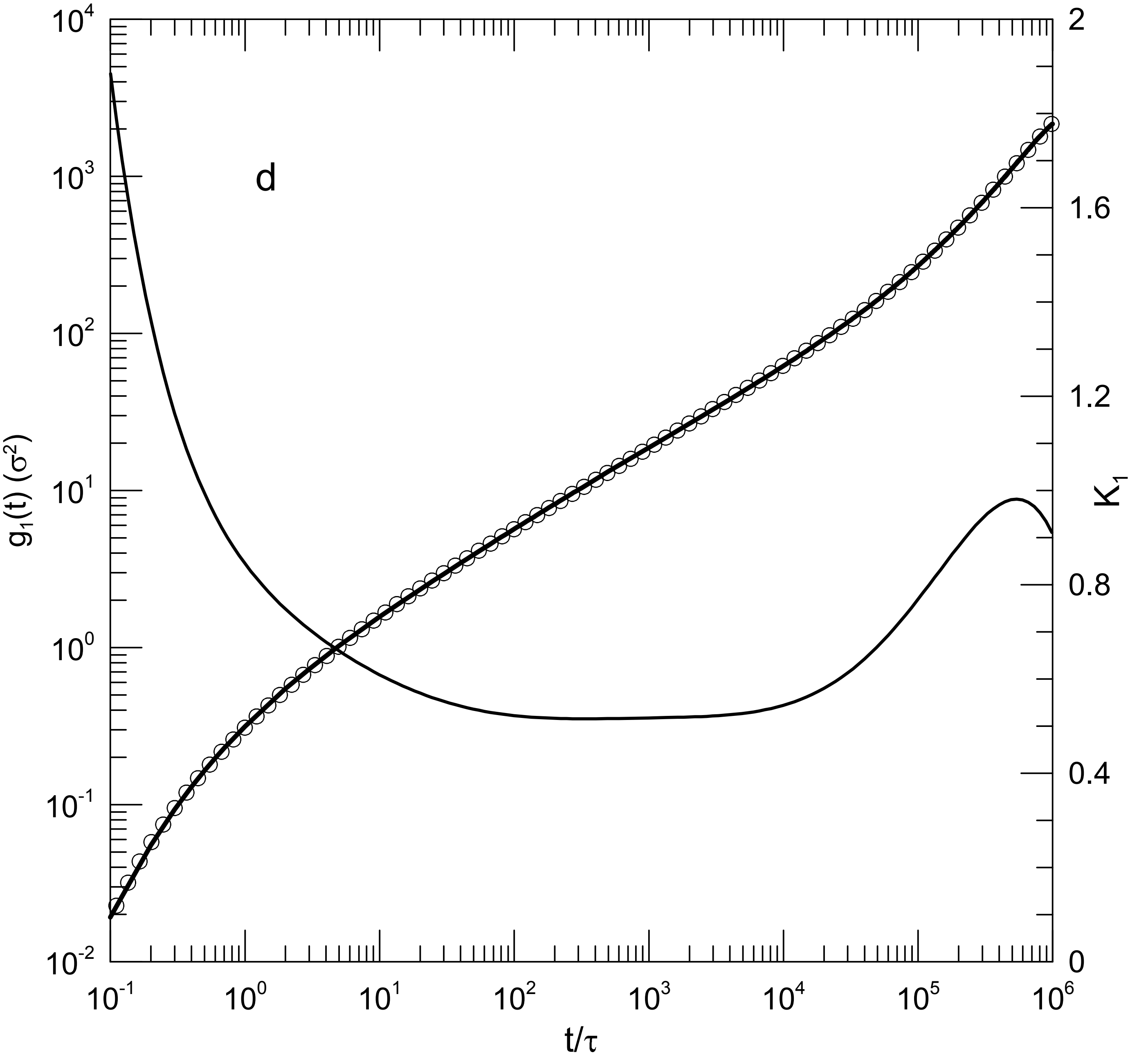

Figure S-2a. Mean-square bead displacements $g_1(t)$ (thick line) of melts of 150-bead Kremer-Grest bead-spring chains, based on simulations of Behbahani and Schmid[1], together with a polynomial fit (circles) and the corresponding first logarithmic derivative K_1 (thin line). The polynomial fit was to an eighth-order polynomial via linear-least-squares.

[h!]

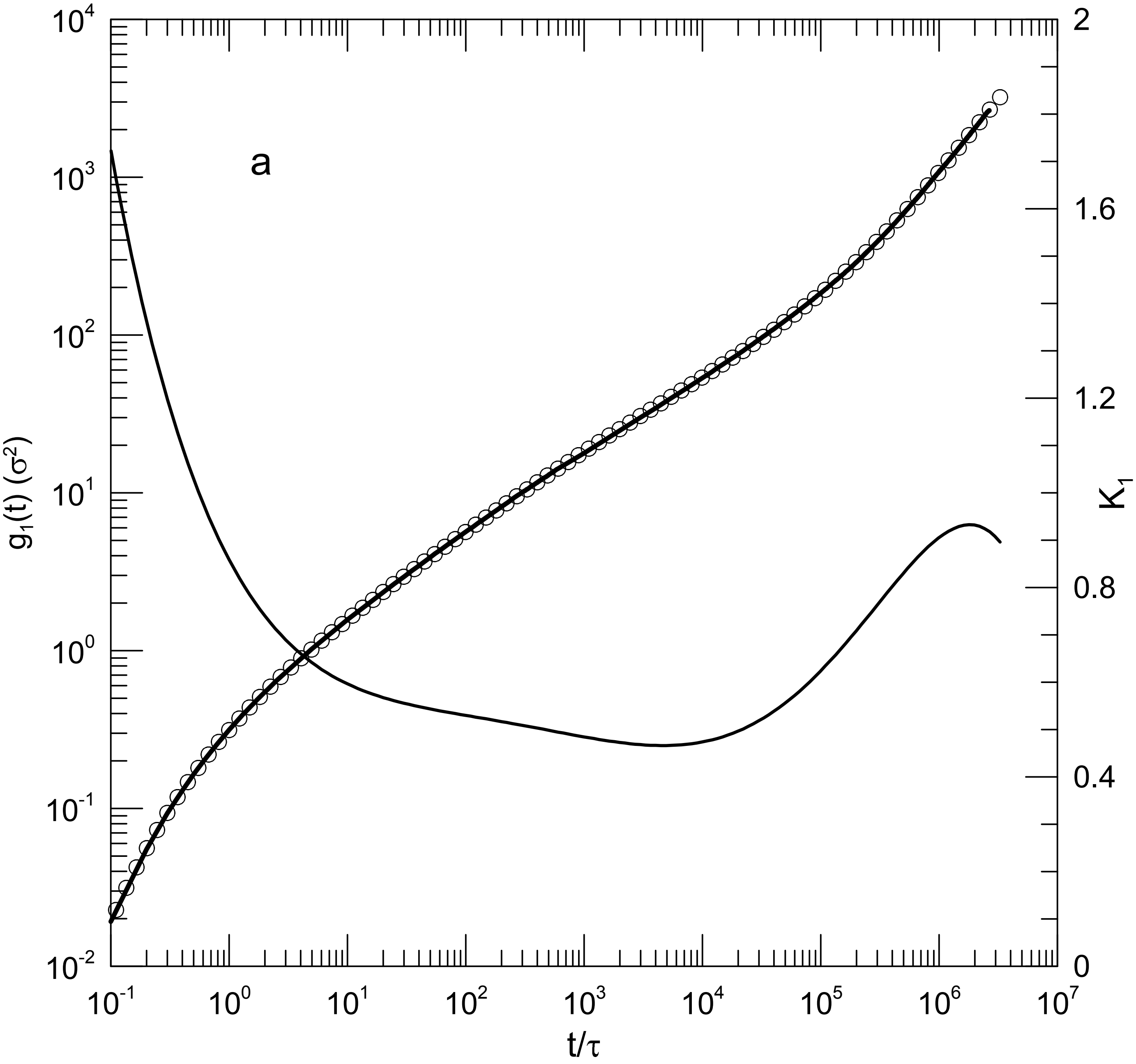

Figure S-2b. Mean-square bead displacements $g_1(t)$ (thick line) of melts of 200-bead Kremer-Grest bead-spring chains, based on simulations of Behbahani and Schmid[1], together with a polynomial fit (circles) and its first logarithmic derivative K_1 (thin line). The polynomial fit was to an eighth-order polynomial via linear-least-squares.

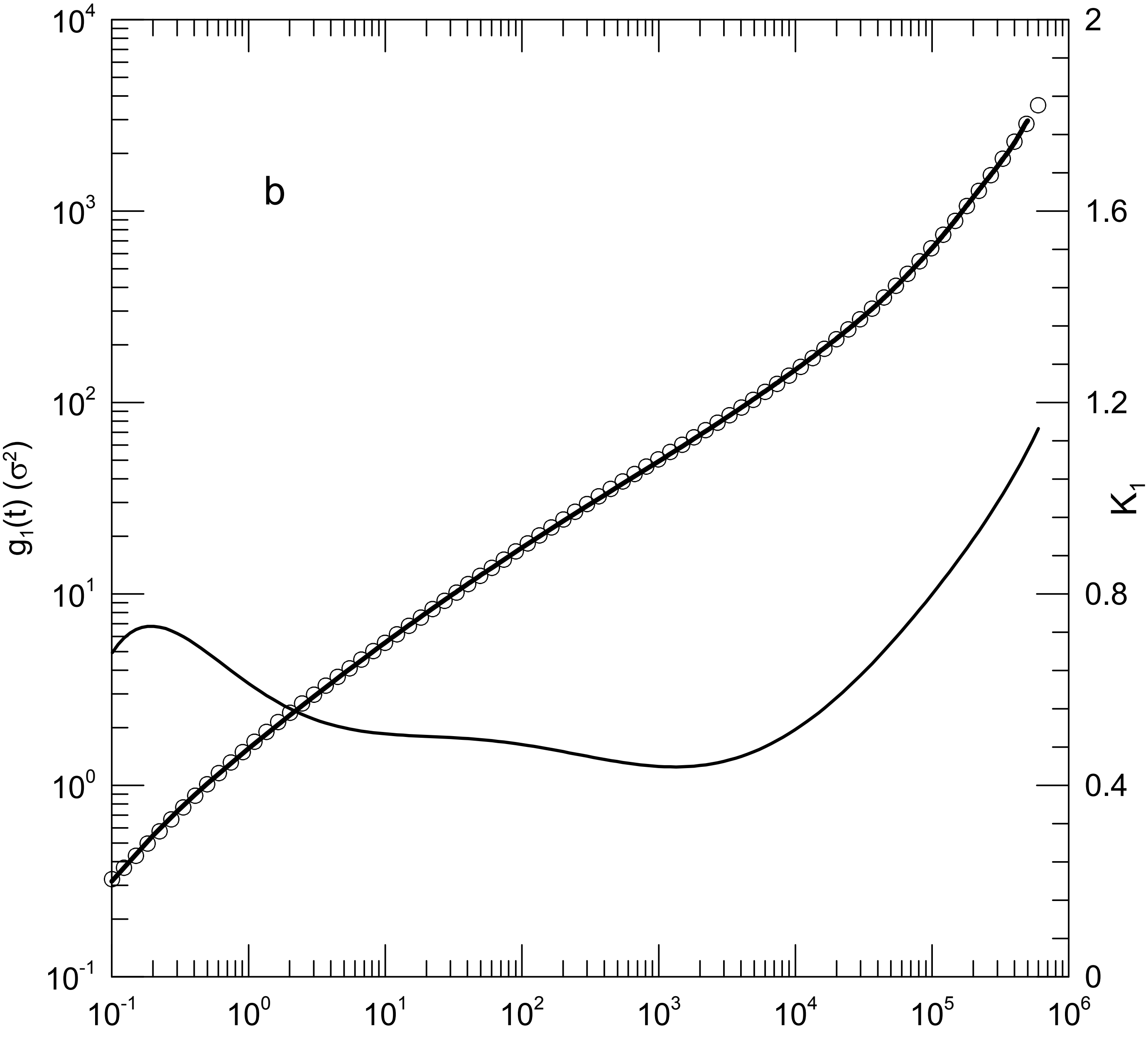

Figure S-2c. Mean-square bead displacements $g_1(t)$ (thick line) of melts of 400-bead Kremer-Grest bead-spring chains, based on simulations of Behbahani and Schmid[1], together with a polynomial fit (circles) and its first logarithmic derivatives K_1 (thin line). The polynomial fit was to an tenth-order polynomial via linear-least-squares.

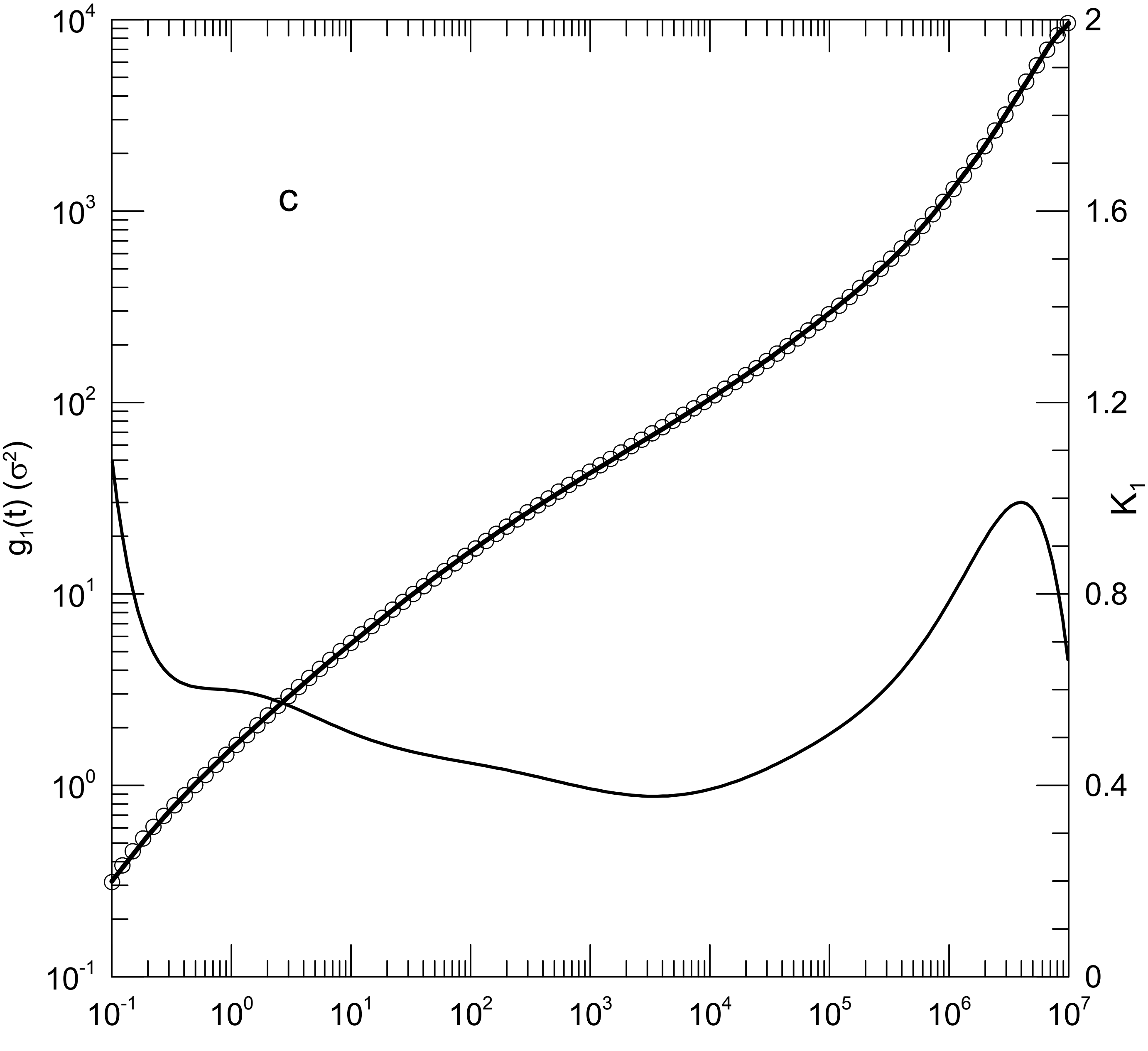

Figure S-2d. Mean-square bead displacements $g_1(t)$ (thick line) of melts of 1000-bead Kremer-Grest bead-spring chains, based on simulations of Behbahani and Schmid[1], together with a polynomial fit (circles) and its first logarithmic derivative K_1 (thin line). The polynomial fit was to an eighth-order polynomial via linear-least-squares.

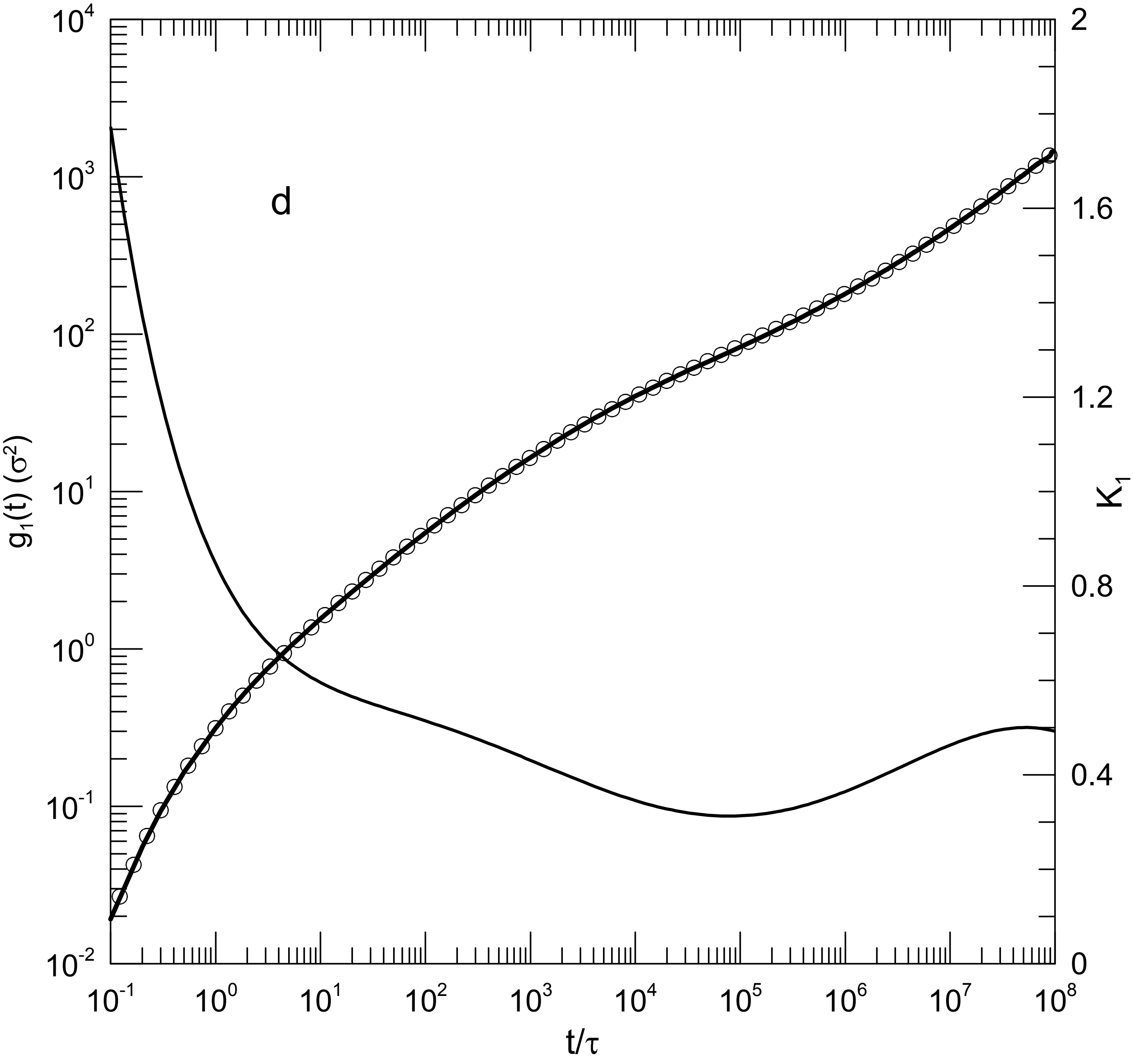

Figure S-3. Mean-square bead displacements $g_1(t)$ of central beads (thick line) of melts of 1000-bead Kremer-Grest bead-spring chains, based on simulations of Behbahani and Schmid[1], together with fits to eighth-order polynomials (circles) and their first logarithmic derivatives K_1 (thin line).

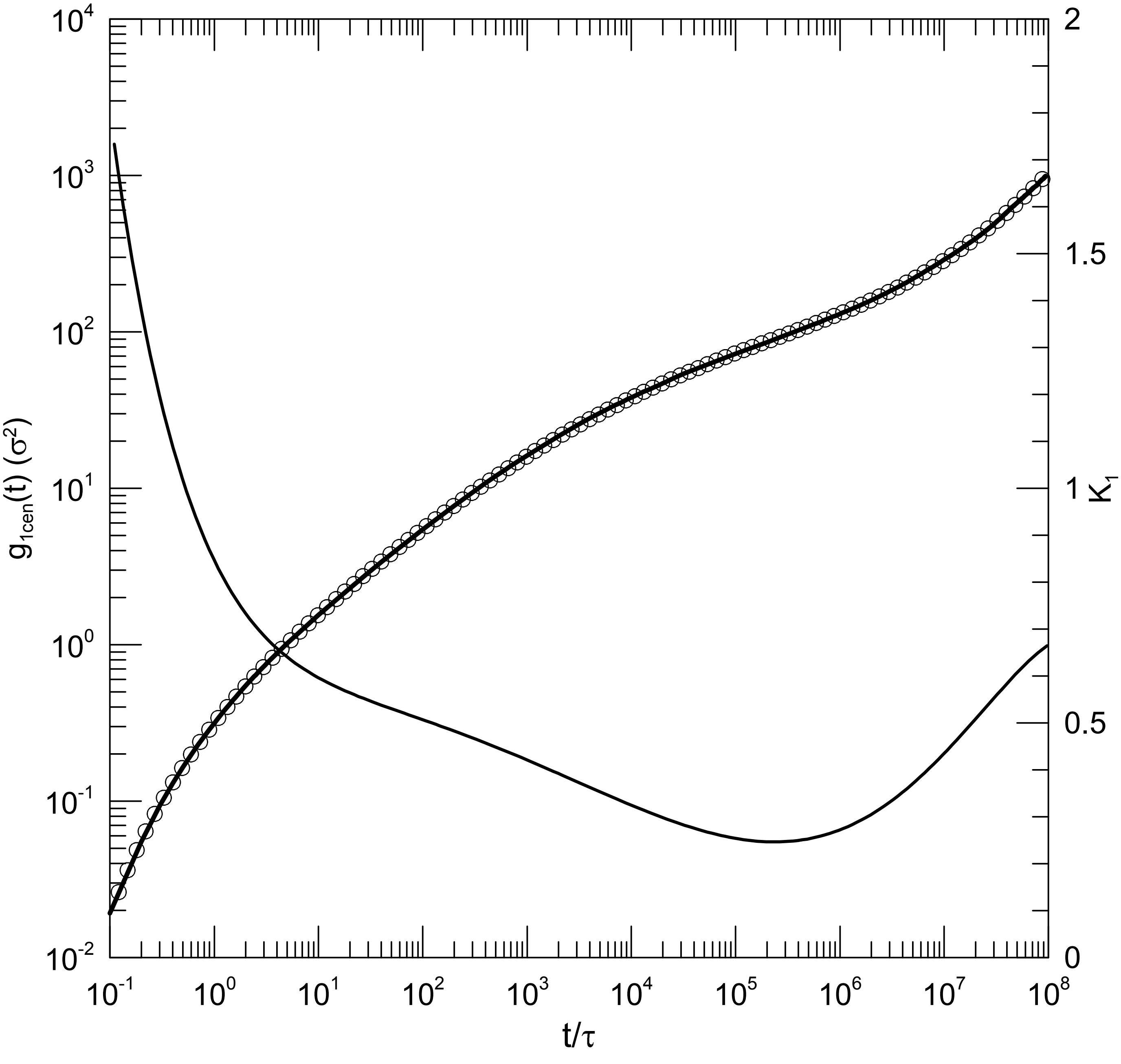

Figure S-4a.Mean-square center-of-mass displacements $g_3(t)$ (thick line) of melts of 10-bead Kremer-Grest bead-spring chains, based on simulations of Behbahani and Schmid[1], together with polynomial fit (circles) and its first logarithmic derivative K_1 (thin line). The fit was to an eighth-order polynomial via linear-least-squares.

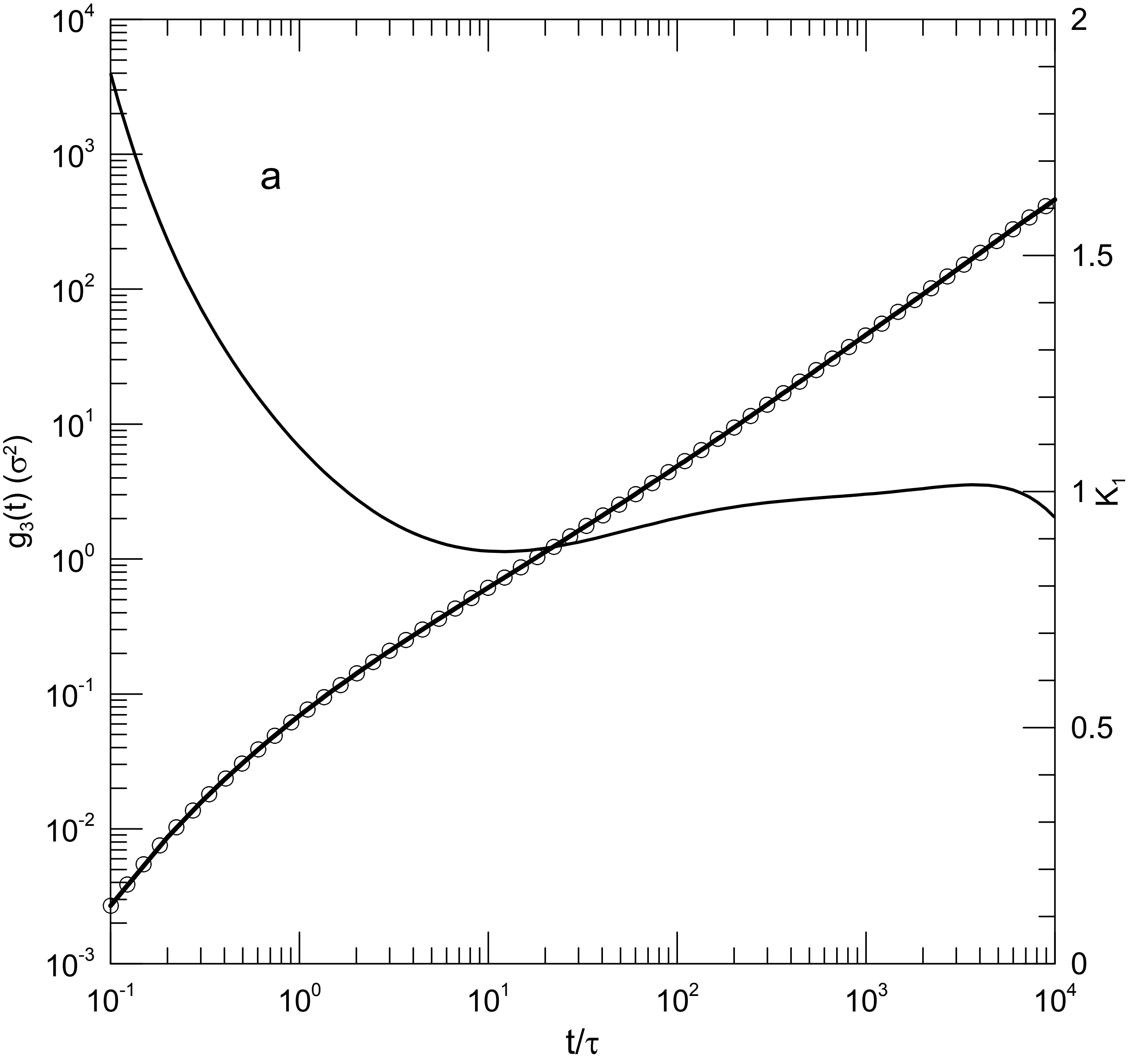

Figure S-4b. Mean-square center-of-mass displacements $g_3(t)$ (thick line) of melts of 30-bead Kremer-Grest bead-spring chains, based on simulations of Behbahani and Schmid[1], together with polynomial fit (circles) and its first logarithmic derivative K_1 (thin line). The fit was to an eighth-order polynomial via linear-least-squares.

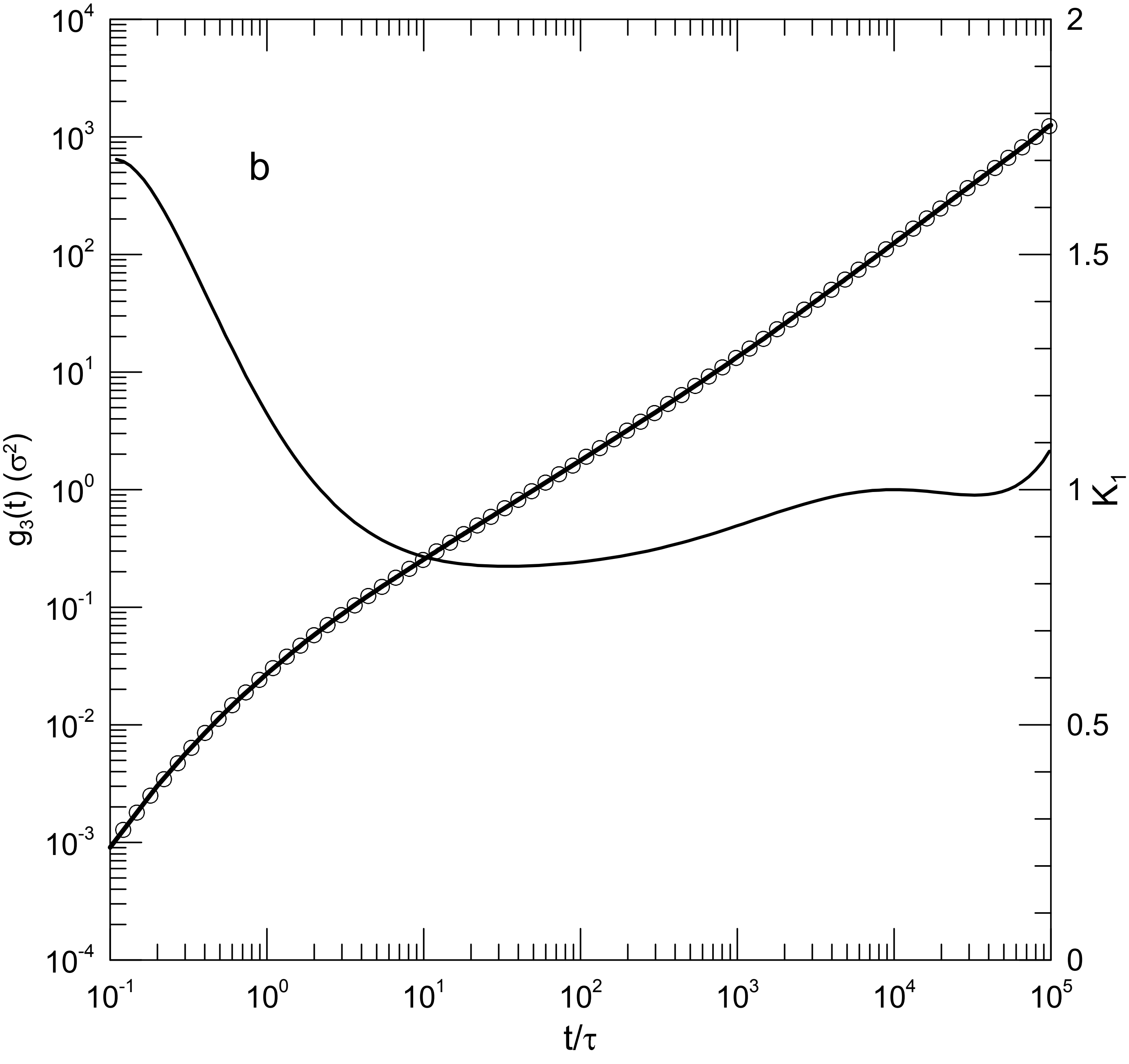

Figure S-4c.Mean-square center-of-mass displacements $g_3(t)$ (thick line under circles) of melts of 50-bead Kremer-Grest bead-spring chains, based on simulations of Behbahani and Schmid[1], together with polynomial fits (circles) and their first logarithmic derivative K_1 (thin line). The fit was to an eighth-order polynomial via linear-least-squares.

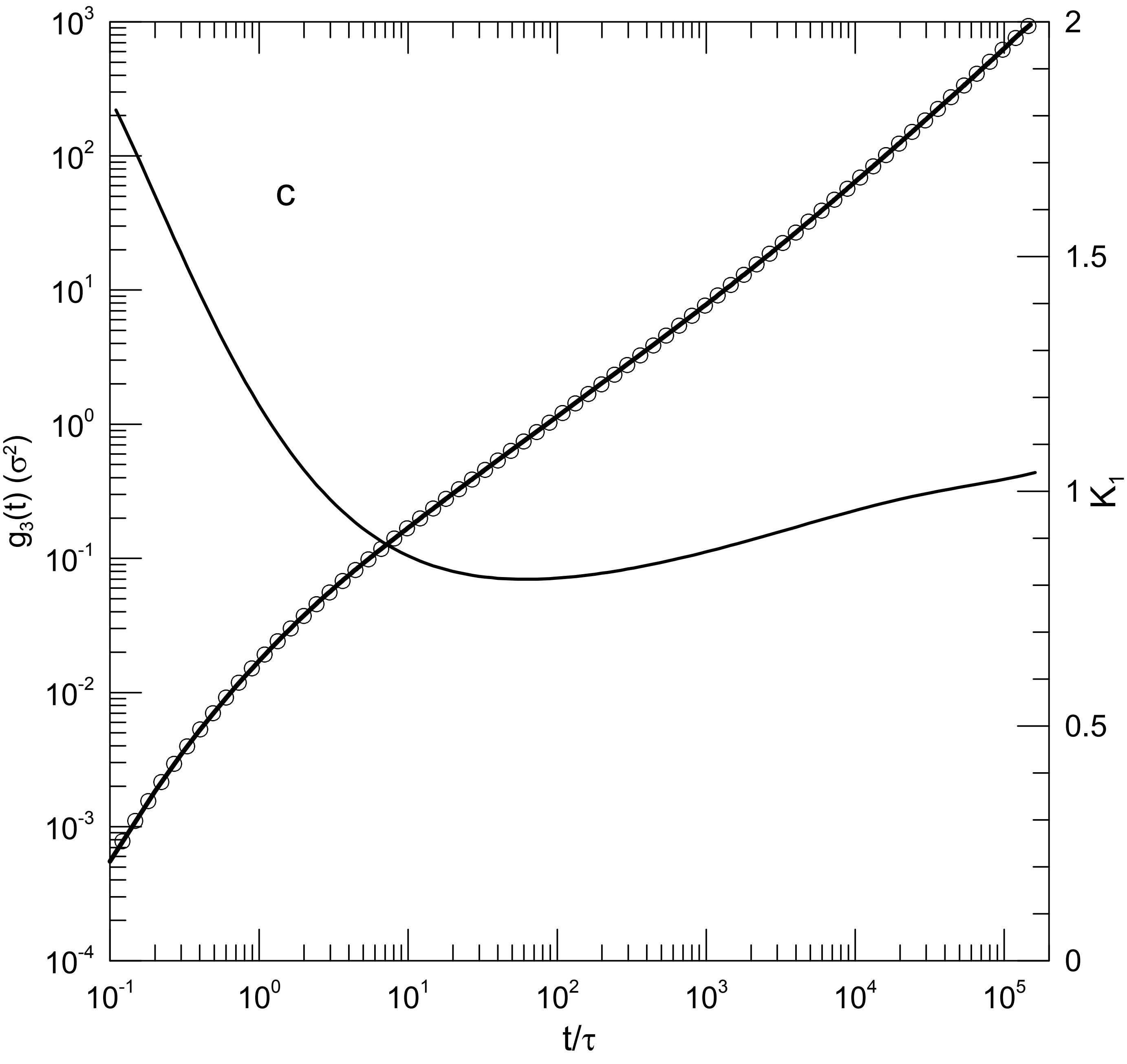

Figure S-4d. Mean-square center-of-mass displacements $g_3(t)$ (thick line) of melts of 100-bead Kremer-Grest bead-spring chains, based on simulations of Behbahani and Schmid[1], together with polynomial fit (circles) and its first logarithmic derivative K_1 (thin line). Chains contained (a) 10, (b) 30, (c) 50, or (d) 100 beads. The fit was to an eighth-order polynomial via linear-least-squares.

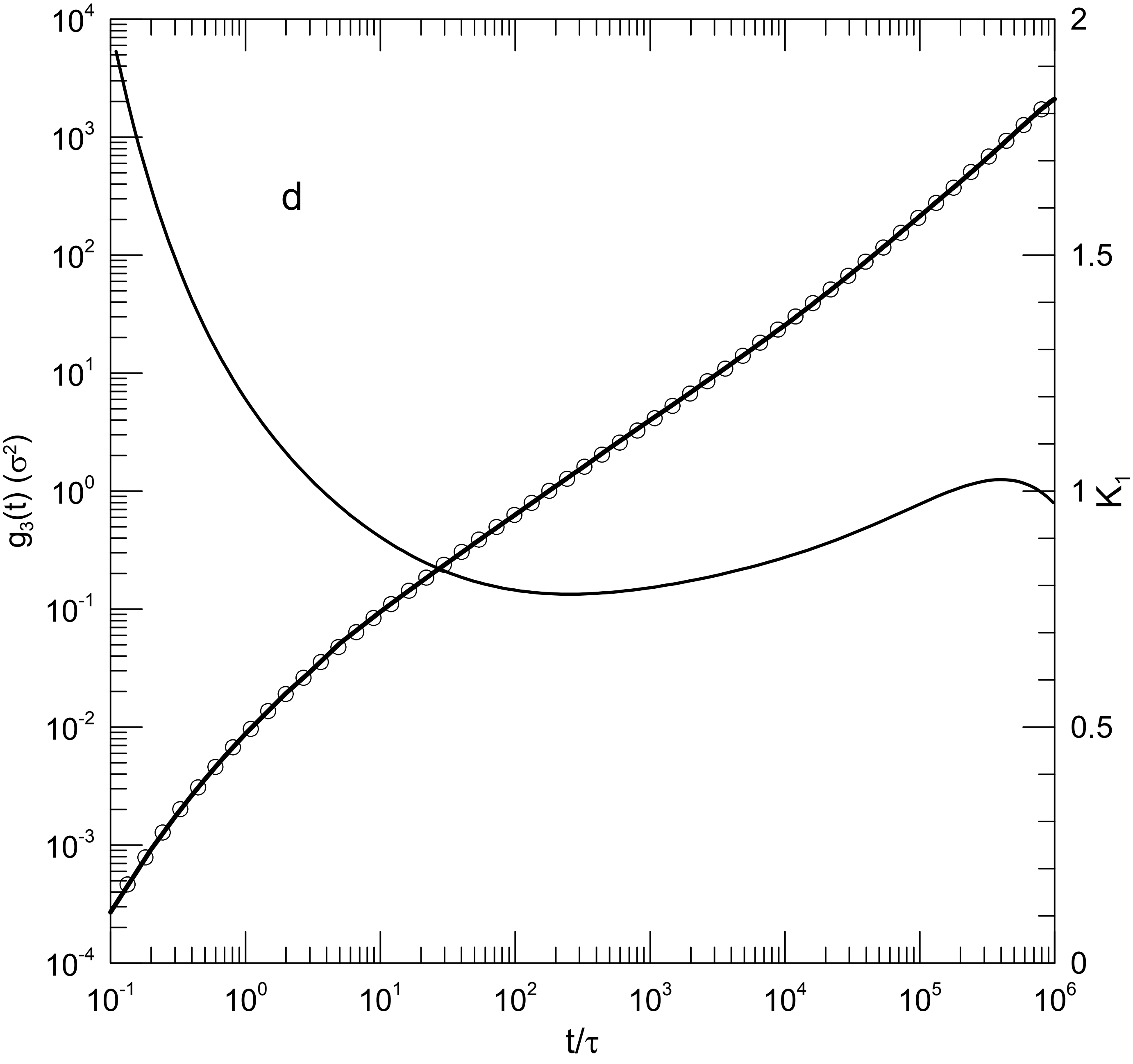

Figure S-5a. Mean-square center-of-mass displacements $g_3(t)$ (thick line) of melts of 150-bead Kremer-Grest bead-spring chains, based on simulations of Behbahani and Schmid[1], together with polynomial fit (circles) and its first logarithmic derivative K_1 (thin line). The polynomial fit was to an eighth-order polynomial via linear-least-squares.

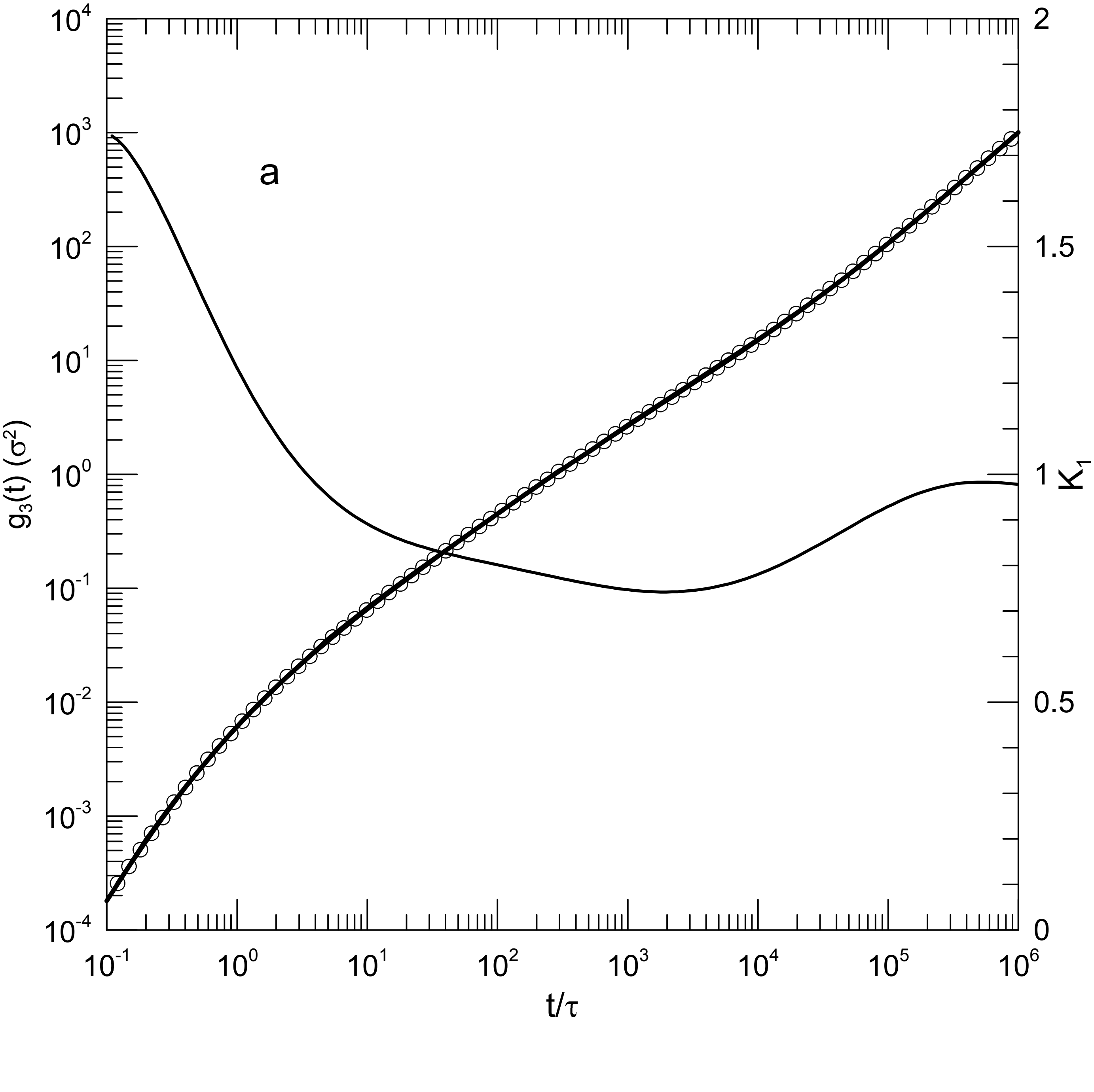

Figure S-5b. Mean-square center-of-mass displacement $g_3(t)$ (thick line) of melts of 200-bead Kremer-Grest bead-spring chains, based on simulations of Behbahani and Schmid[1], together with polynomial fit (circles) and its first logarithmic derivative K_1 (thin line). The polynomial fit was to an eighth-order polynomial via linear-least-squares.

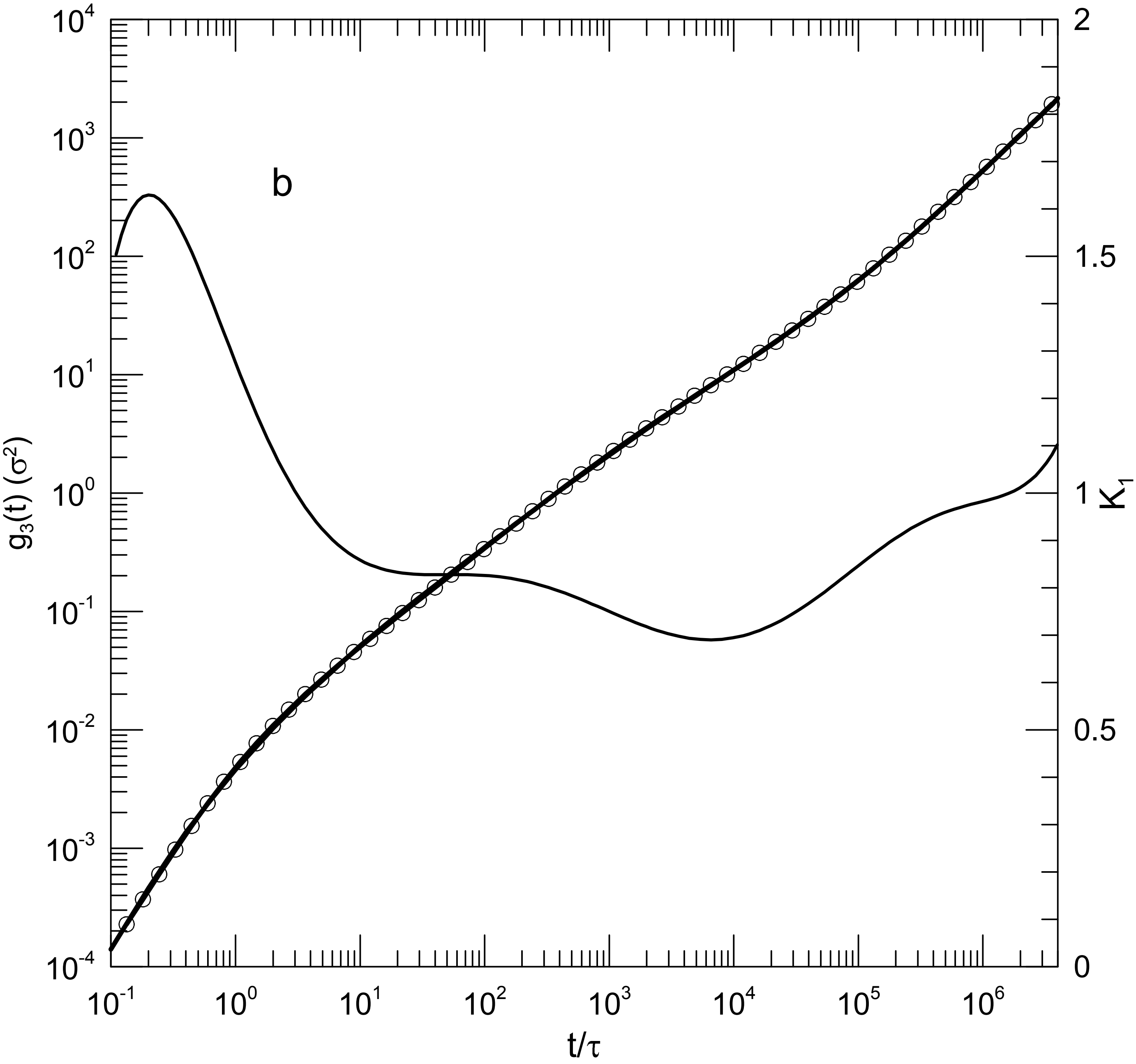

Figure S-5c. Mean-square center-of-mass displacement $g_3(t)$ (thick line) of melts of 400-bead Kremer-Grest bead-spring chains, based on simulations of Behbahani and Schmid[1], together with polynomial fit (circles) and its first logarithmic derivative K_1 (thin line). The polynomial fit was to an eighth-order polynomial via linear-least-squares.

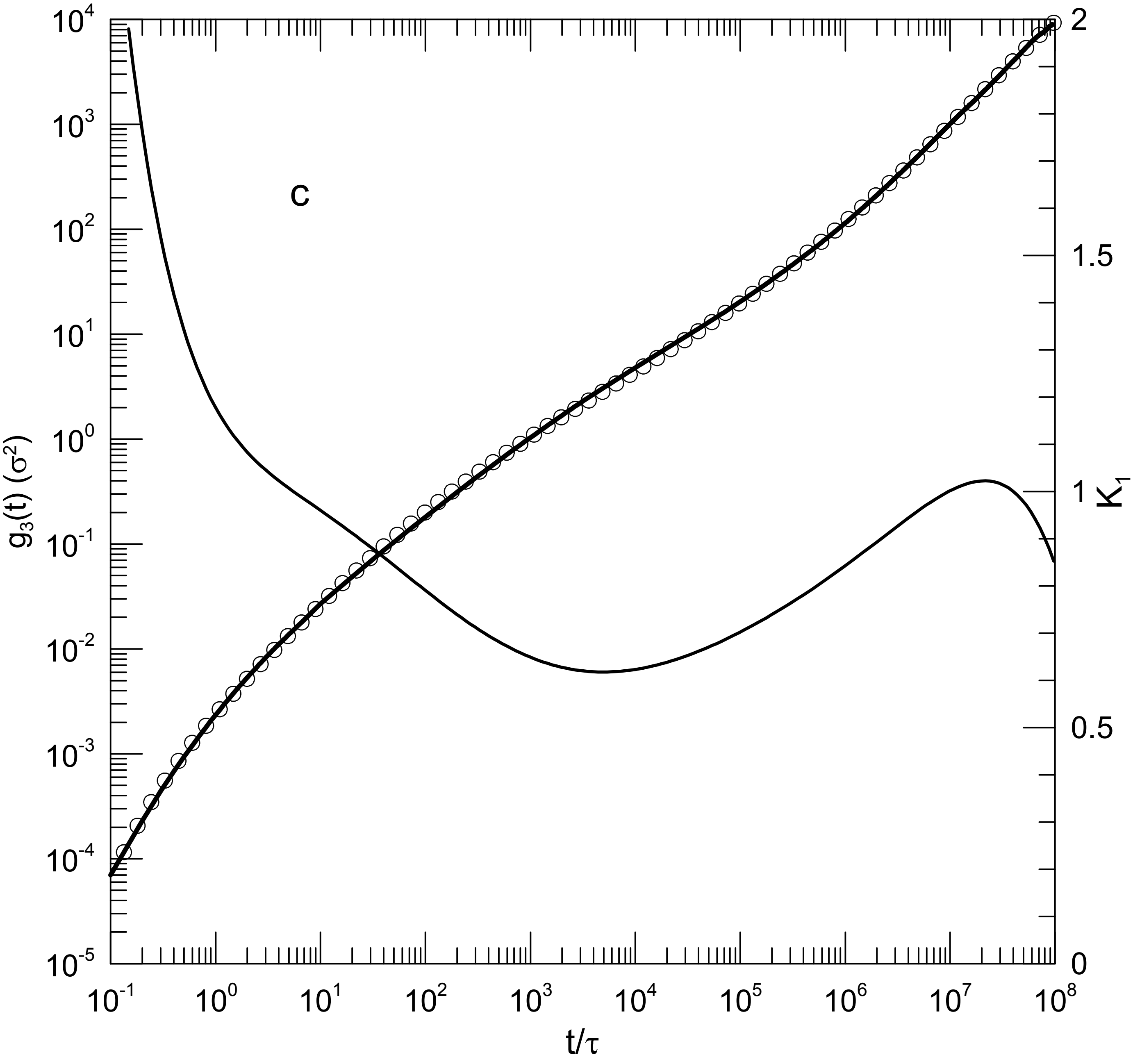

Figure S-5d. Mean-square center-of-mass displacement $g_3(t)$ (thick line) of melts of 1000-bead Kremer-Grest bead-spring chains, based on simulations of Behbahani and Schmid[1], together with polynomial fit (circles) and its first logarithmic derivative K_1 (thin line). The polynomial fit was to an eighth-order polynomial via linear-least-squares.

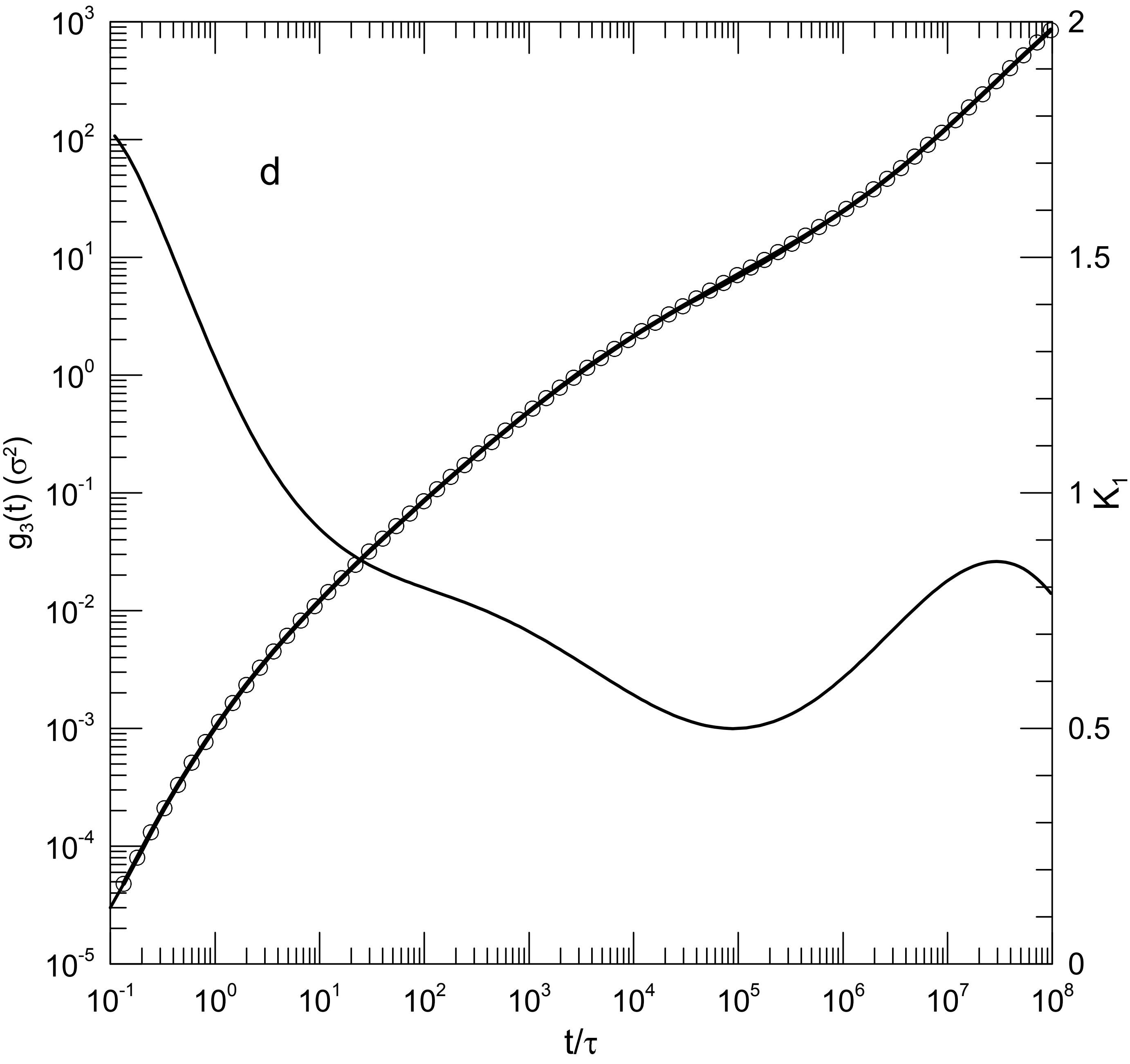

Figure S-6a. Mean-square center-of-mass displacement (thick line) of a melt of 256-bead chains with chain stretch parameter $\delta_B = 0.45$, based on simulations by Chang and Yethiraj[2], together with a fit to an eighth-order polynomial (open circles) and its first logarithmic derivative K_1 (thin line).

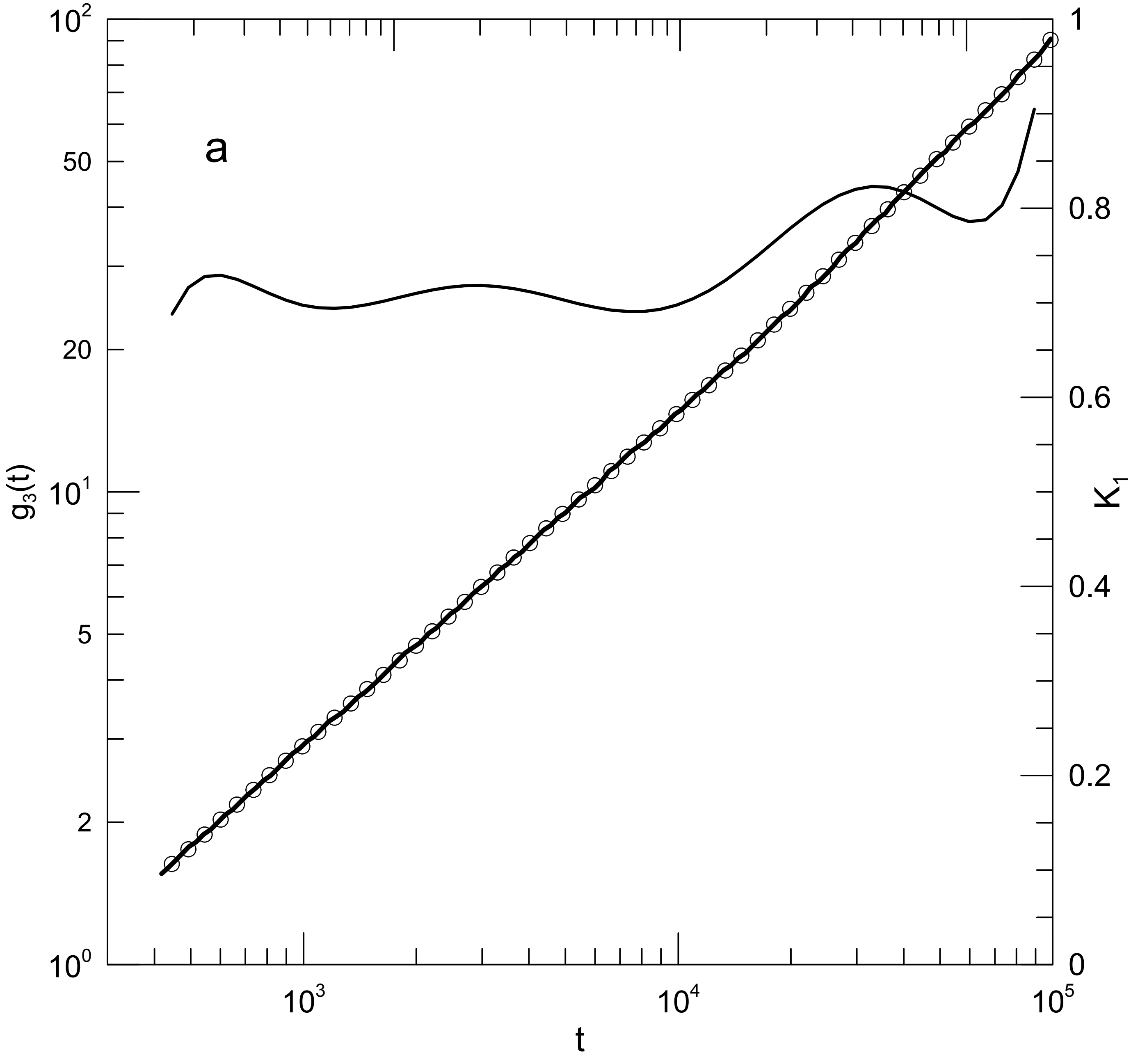

Figure S-6b. Mean-square center-of-mass displacement (thick line) of a melt of 256-bead chains with chain stretch parameter $\delta_B = 0.5$, based on simulations by Chang and Yethiraj[2], together with a fit to an eighth-order polynomial (open circles) and its first logarithmic derivative K_1 (thin line).

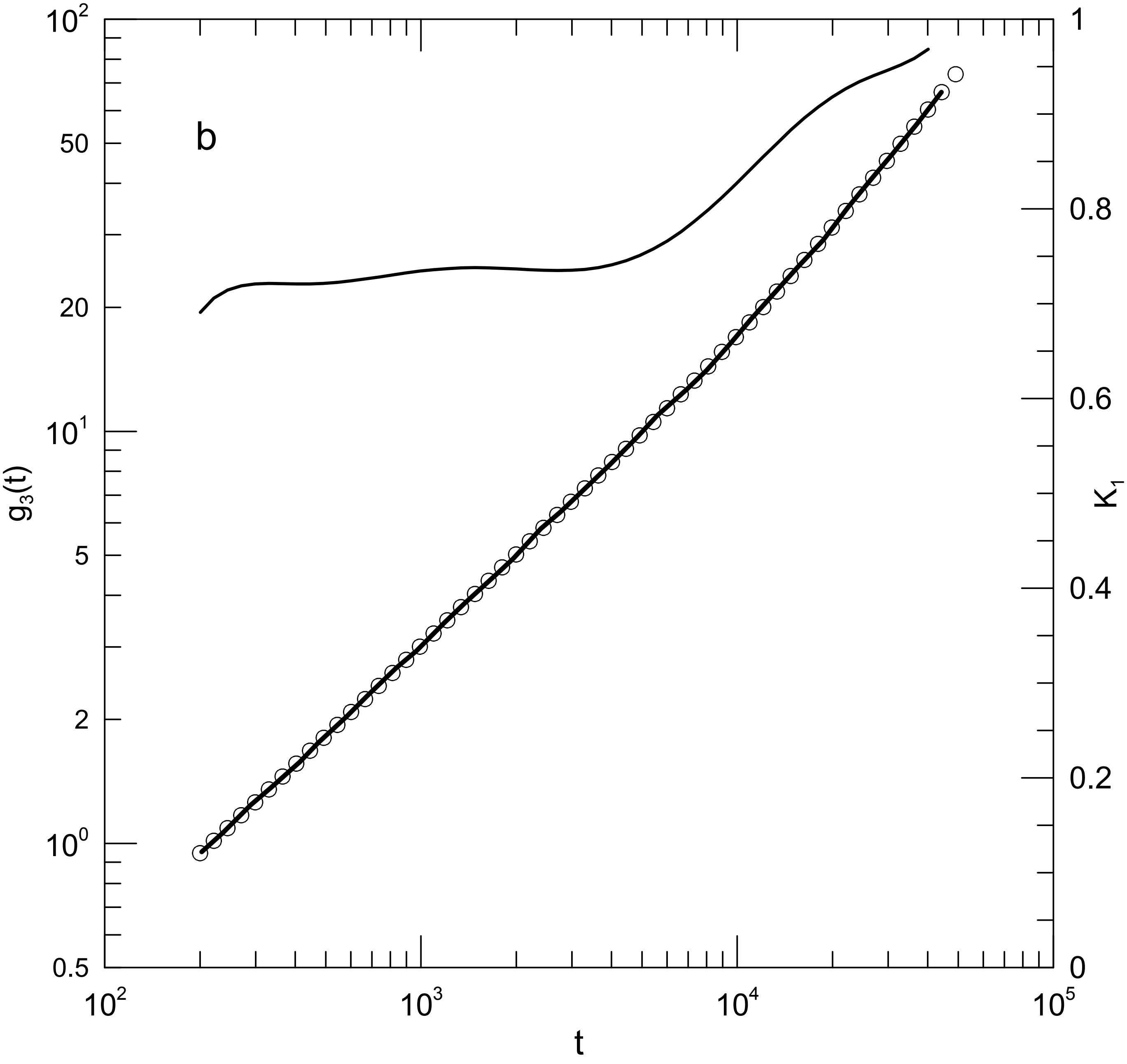

Figure S-6c. Mean-square center-of-mass displacement (thick line) of a melt of 256-bead chains with chain stretch parameter $\delta_B = 0.6$, based on simulations by Chang and Yethiraj[2], together with a fit to an eighth-order polynomial (open circles) and its first logarithmic derivative K_1 (thin line).

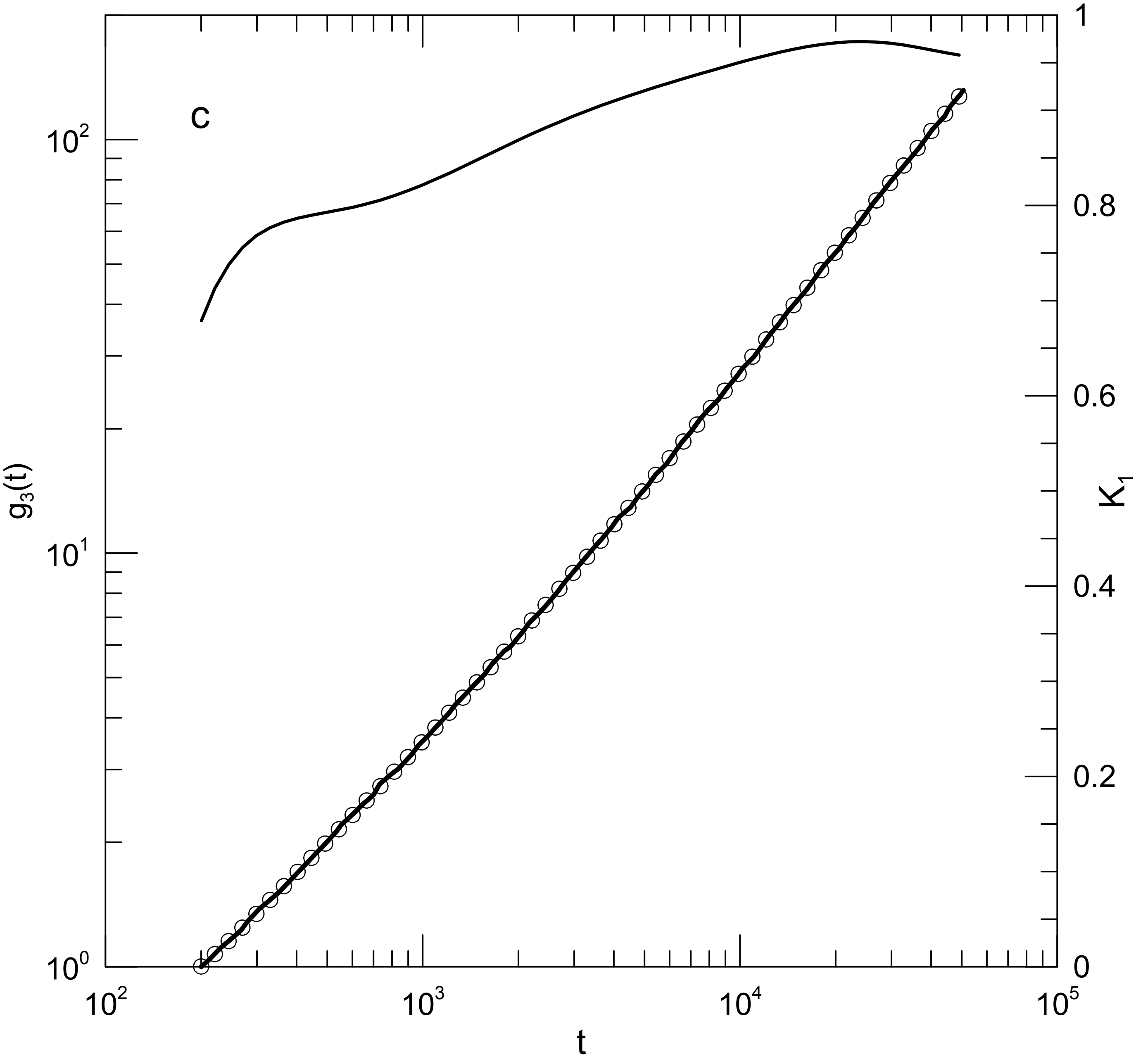

Figure S-6d. Mean-square central beads displacement (thick line) of a melt of 256-bead chains with chain stretch parameter $\delta_B = 0.45$, based on simulations by Chang and Yethiraj[2], together with a fit to an eighth-order polynomial (open circles) and its first logarithmic derivative K_1 (thin line).

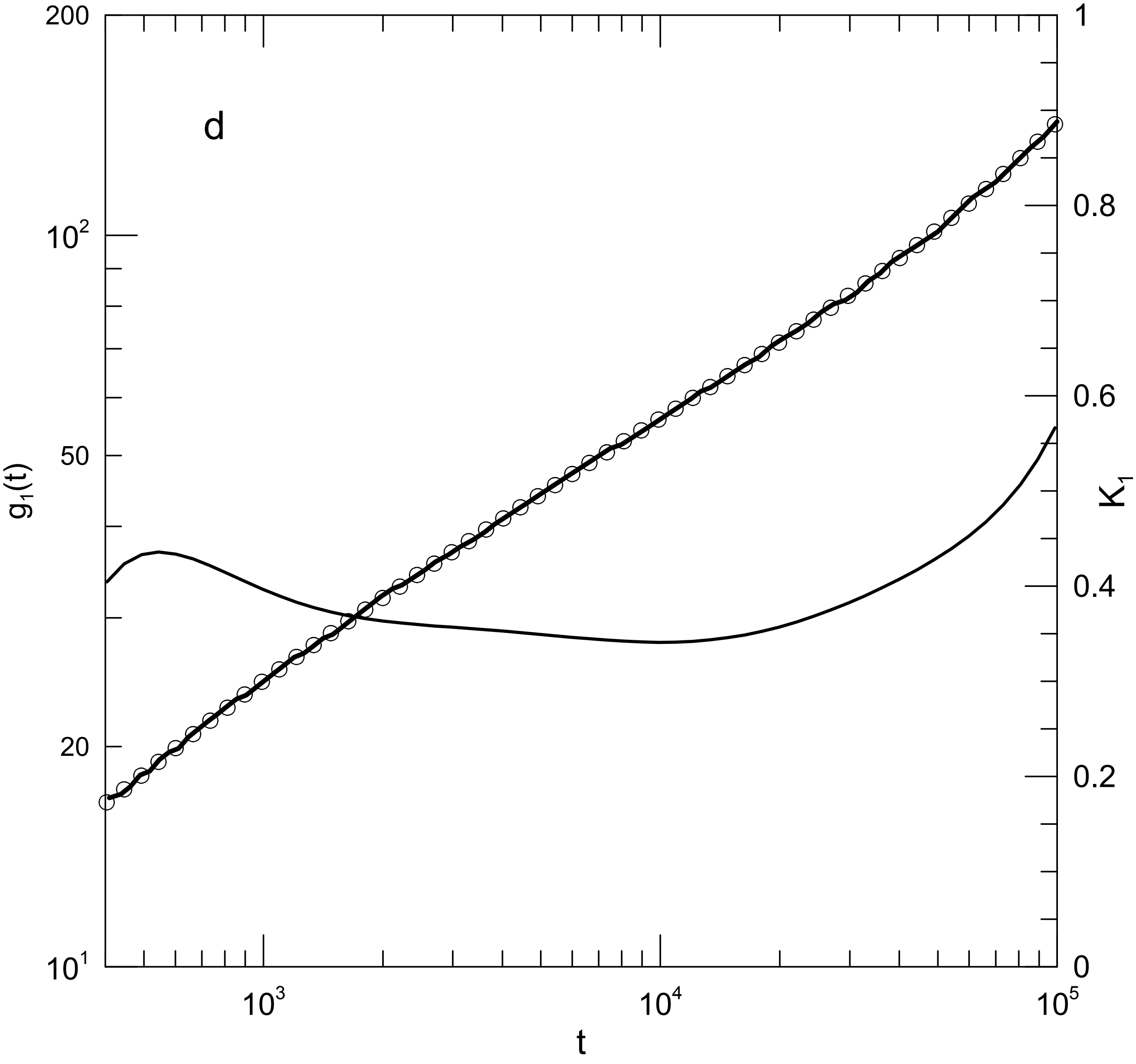

Figure S-6e. Mean-square central-beads displacement (thick line) of a melt of 256-bead chains with chain stretch parameter $\delta_B = 0.5$, based on simulations by Chang and Yethiraj[2], together with a fit to an eighth-order polynomial (open circles) and its first logarithmic derivative K_1 (thin line).

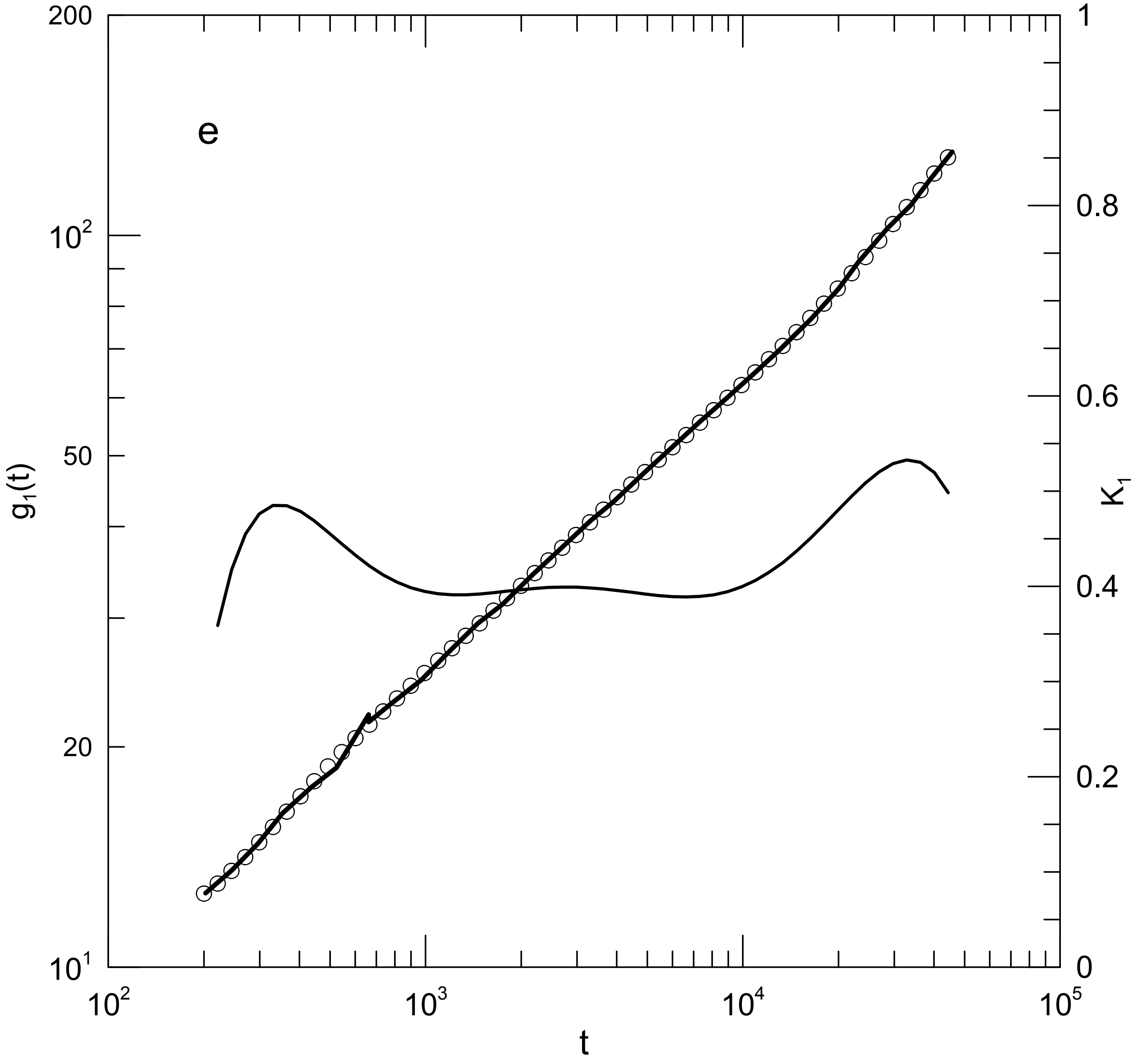

Figure S-6f. Mean-square central-beads displacement (thick line) of a melt of 256-bead chains with chain stretch parameter $\delta_B = 0.6$, based on simulations by Chang and Yethiraj[2], together with a fit to an eighth-order polynomial (open circles) and its first logarithmic derivative K_1 (thin line).

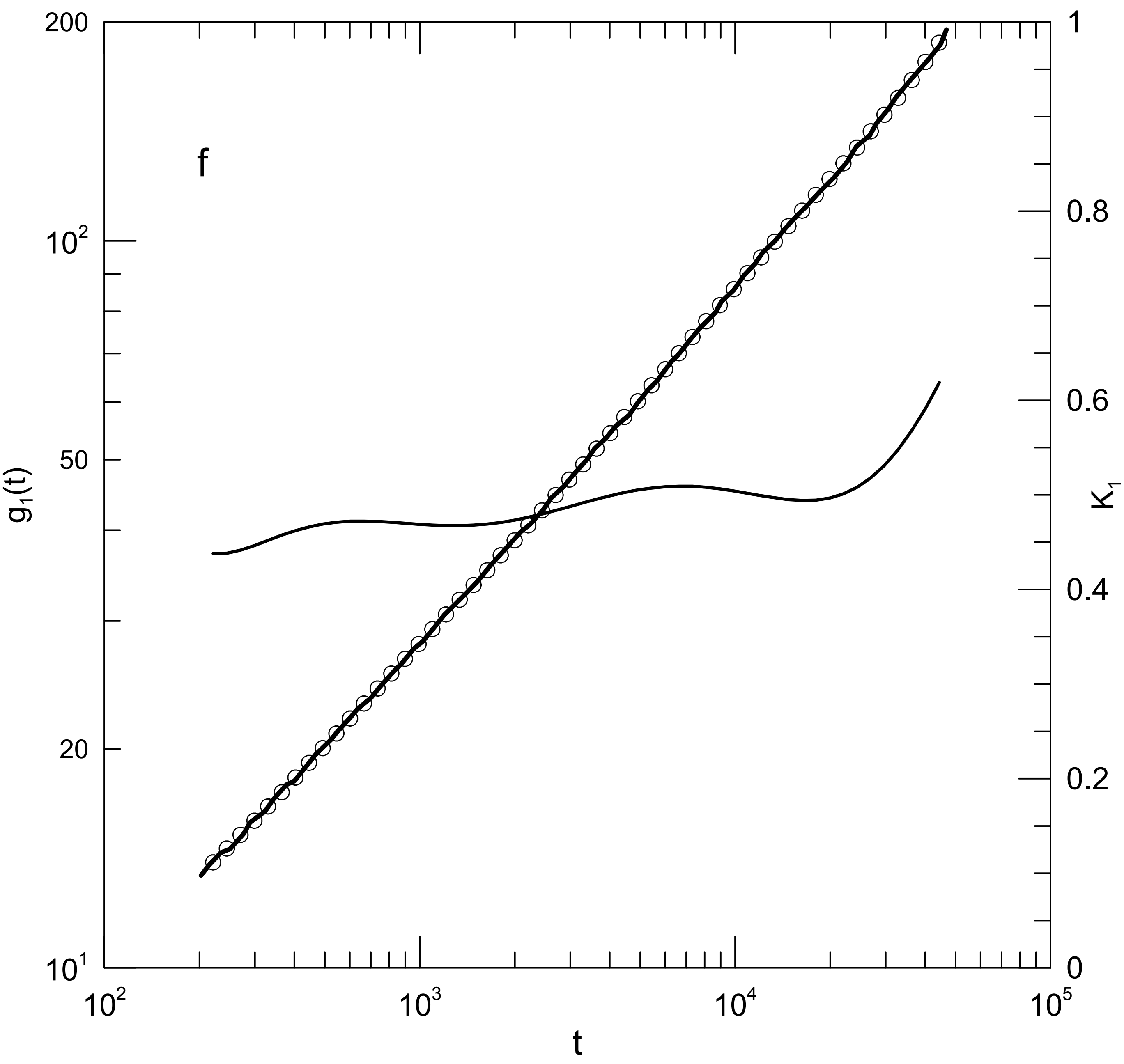

Figure S-7a. Mean-square bead displacement (heavy line) of linear 120-bead polyethylene oxide, from the united-atom simulations of Tsalikis, et al.[3], together with fits to eighth-order polynomials (circles) and their first logarithmic derivatives K_1 (thin line).

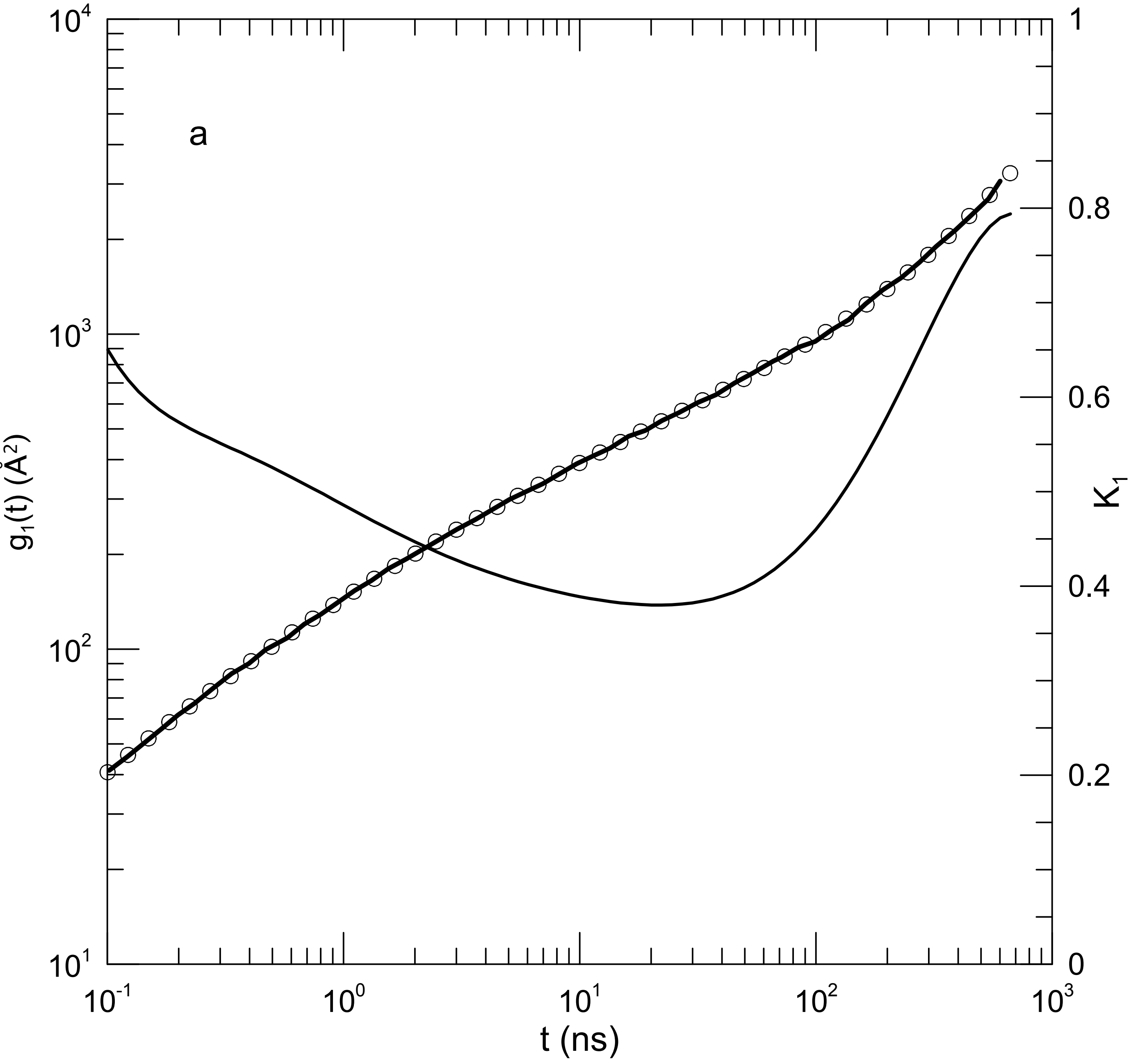

Figure S-7b. Mean-square bead displacement (heavy line) of ring 120-bead polyethylene oxide, from the united-atom simulations of Tsalikis, et al.[3], together with fits to eighth-order polynomials (circles) and their first logarithmic derivatives K_1 (thin line).

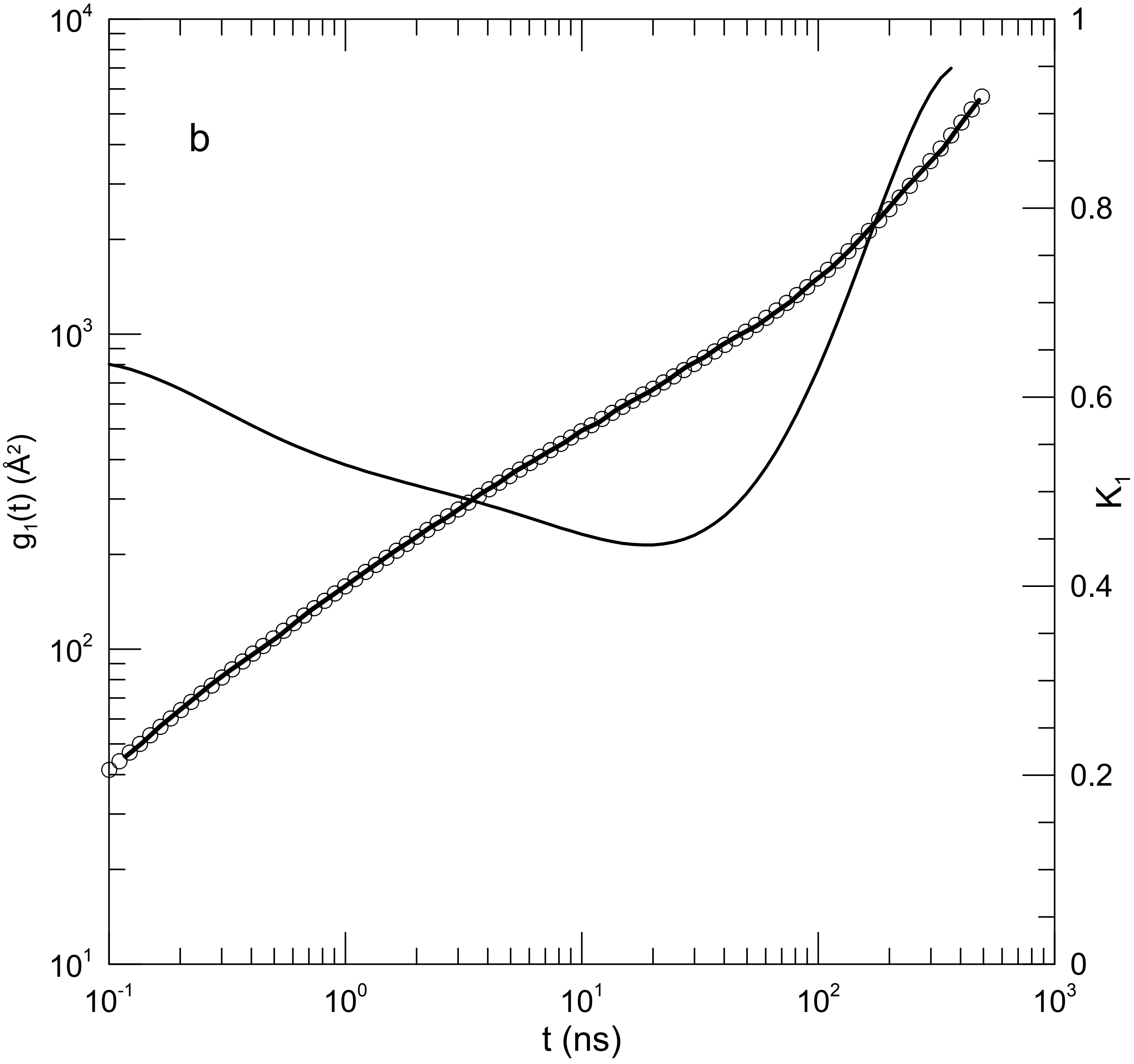

Figure S-7c. Mean-square bead displacement (heavy line) of linear 227-bead polyethylene oxide, from the united-atom simulations of Tsalikis, et al.[3], together with fits to eighth-order polynomials (circles) and their first logarithmic derivatives K_1 (thin line).

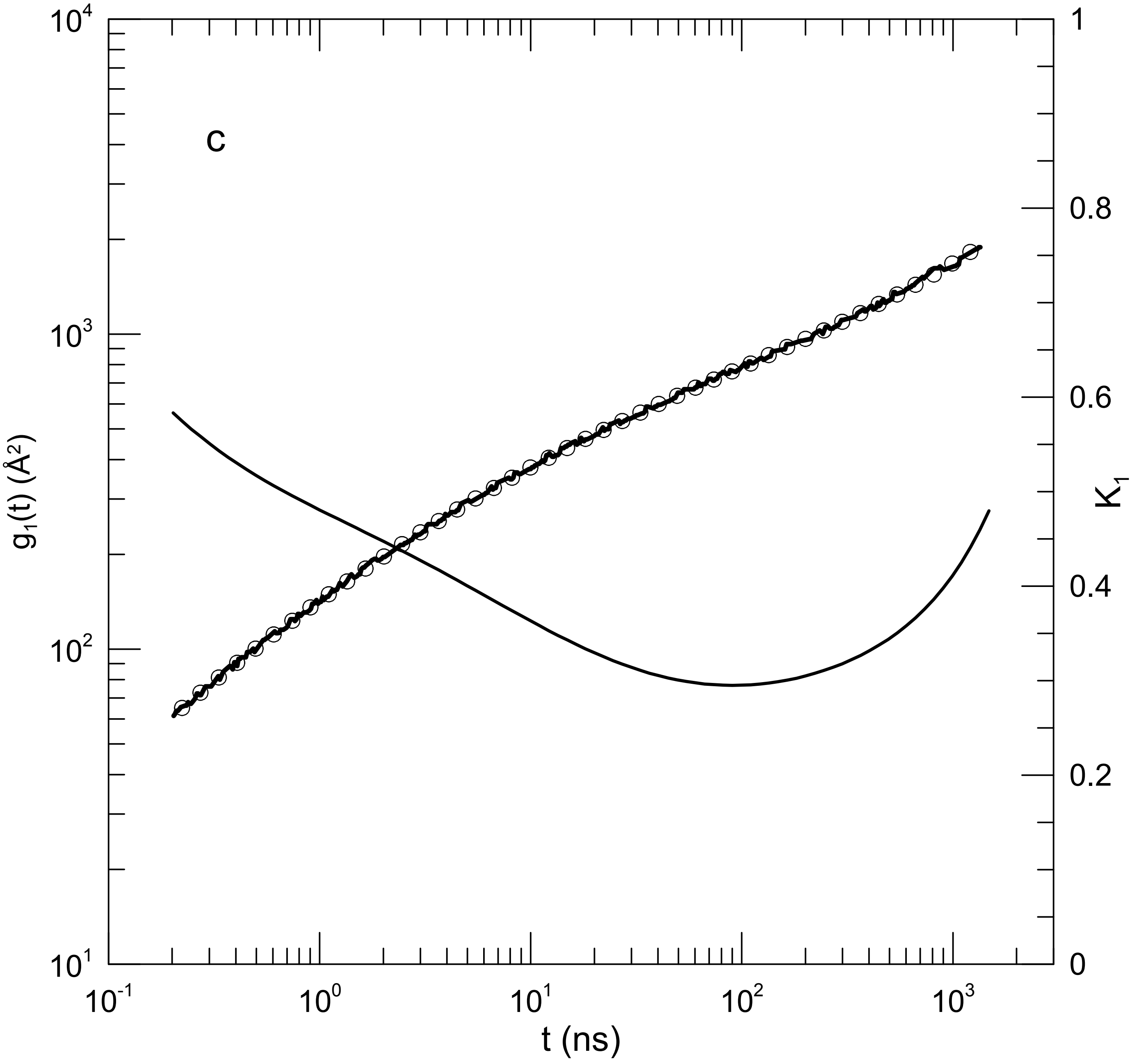

Figure S-7d. Mean-square bead displacement (heavy line) of ring 227-bead polyethylene oxide, from the united-atom simulations of Tsalikis, et al.[3], together with fits to eighth-order polynomials (circles) and their first logarithmic derivatives K_1 (thin line).

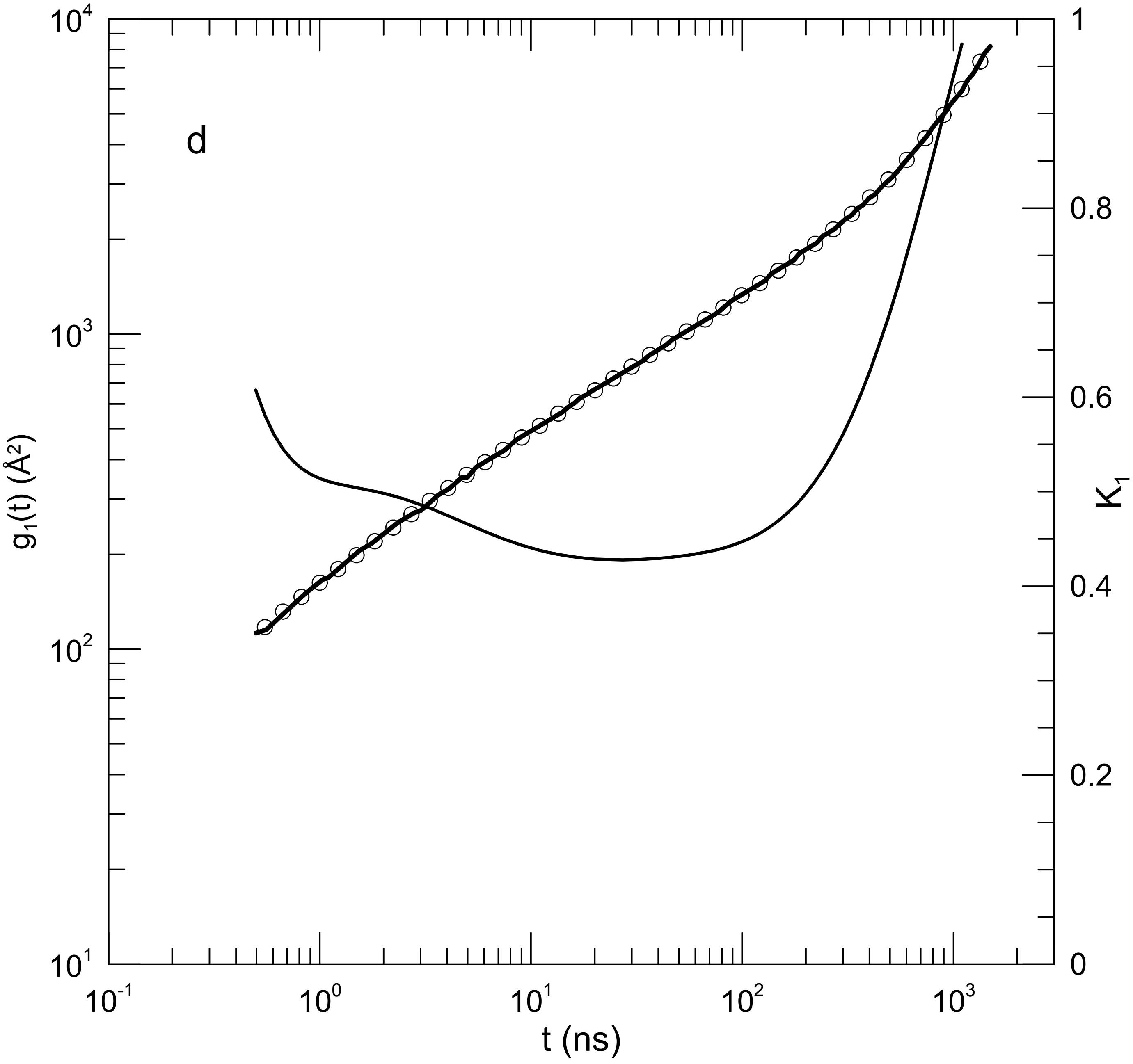

Figure S-7e. Mean-square bead displacement (heavy line) of linear 455-bead polyethylene oxide, from the united-atom simulations of Tsalikis, et al.[3], together with fits to eighth-order polynomials (circles) and their first logarithmic derivatives K_1 (thin line).

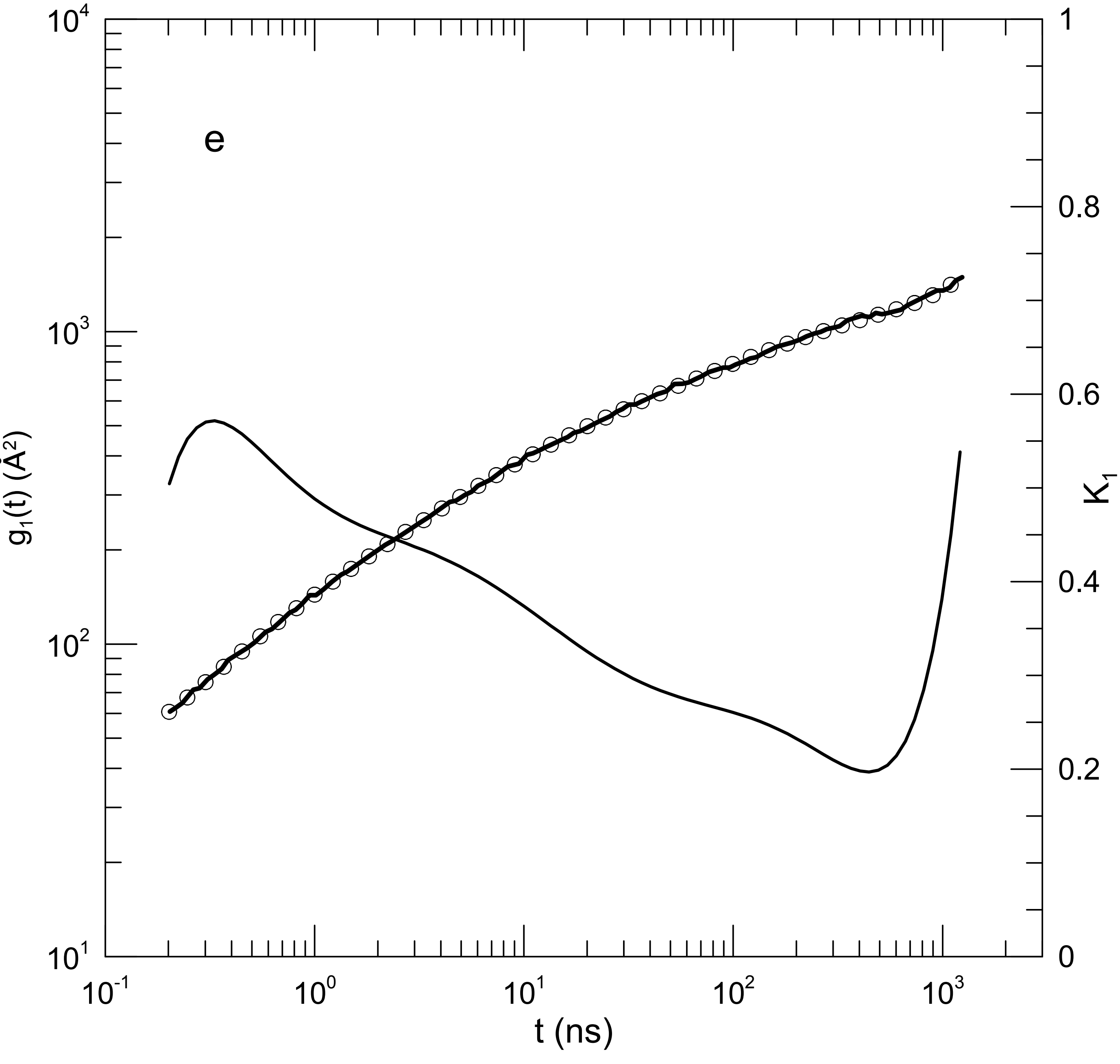

Figure S-7f. Mean-square bead displacement (heavy line) of ring 455-bead polyethylene oxide, from the united-atom simulations of Tsalikis, et al.[3], together with fits to eighth-order polynomials (circles) and their first logarithmic derivatives K_1 (thin line).

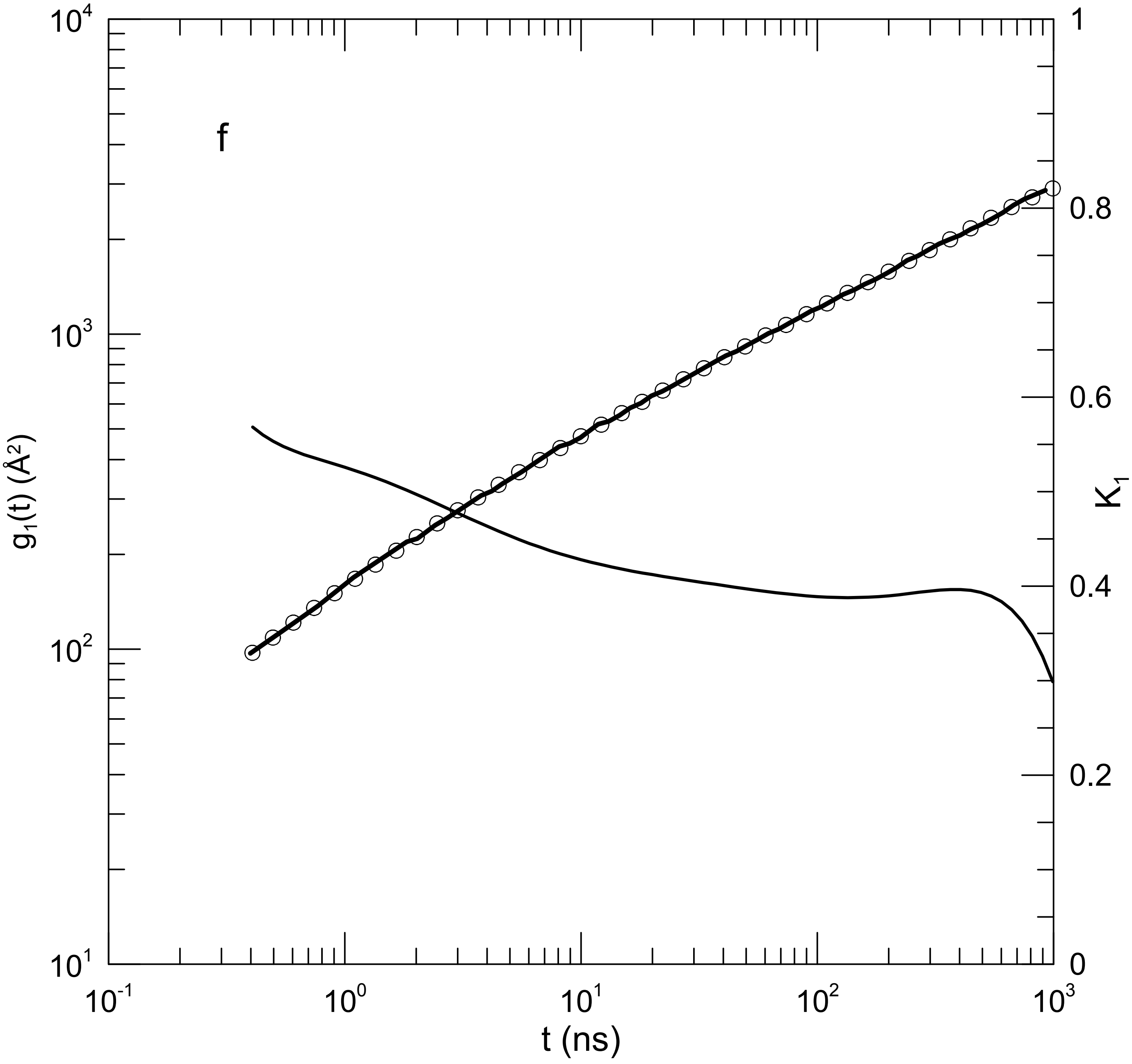

Figure S-8a. Mean-square bead displacement (thick line) of 422 Da linear polyethylenes from Takahashi, et al.[4], together with an eighth-order polynomial fit(circles) and its first logarithmic derivative K_1 (thin line).

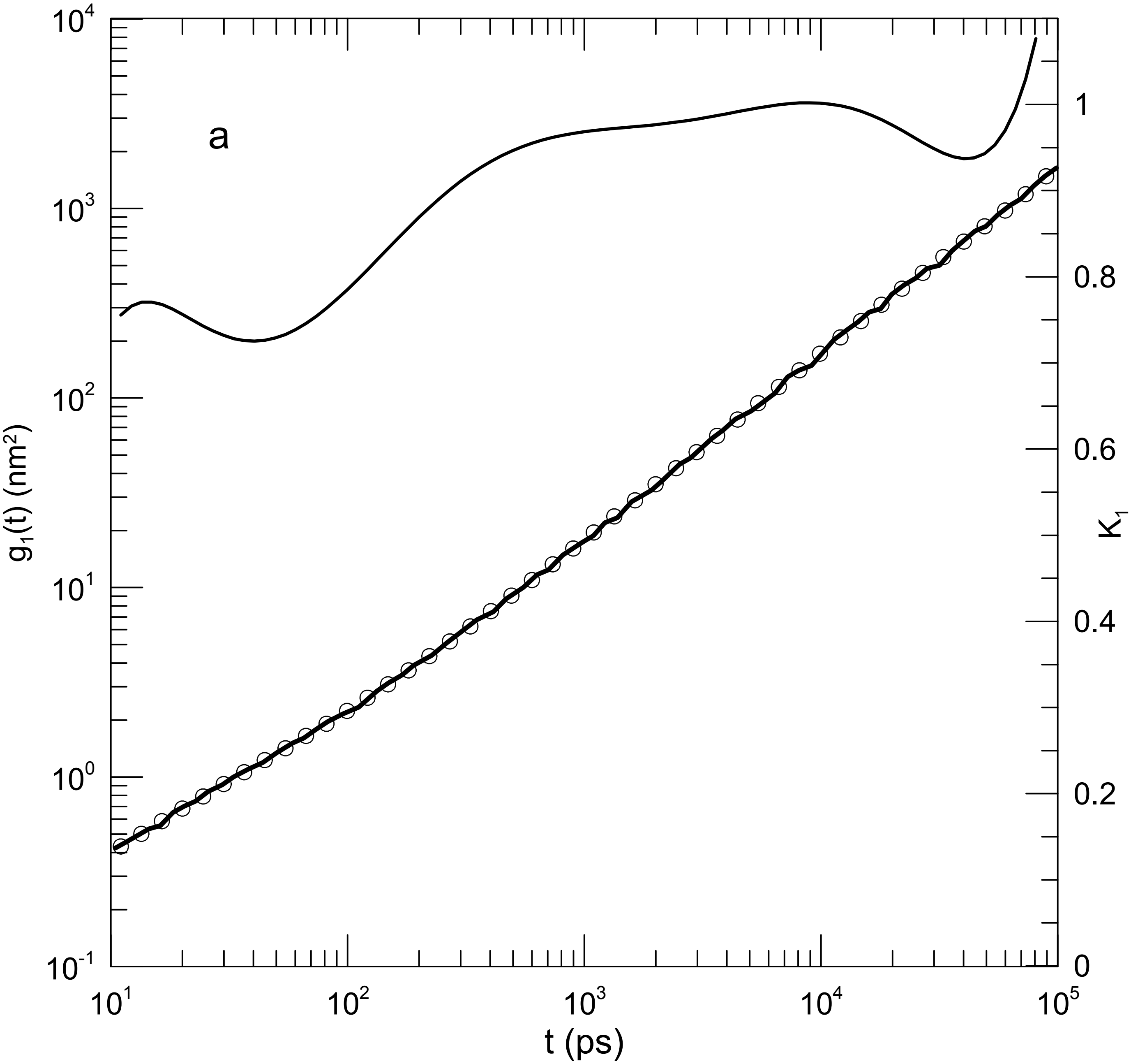

Figure S-8b. Mean-square bead displacement (thick line) of 703 Da linear polyethylenes from Takahashi, et al.[4], together with an eighth-order polynomial fit(circles) and its first logarithmic derivative K_1 (thin line).

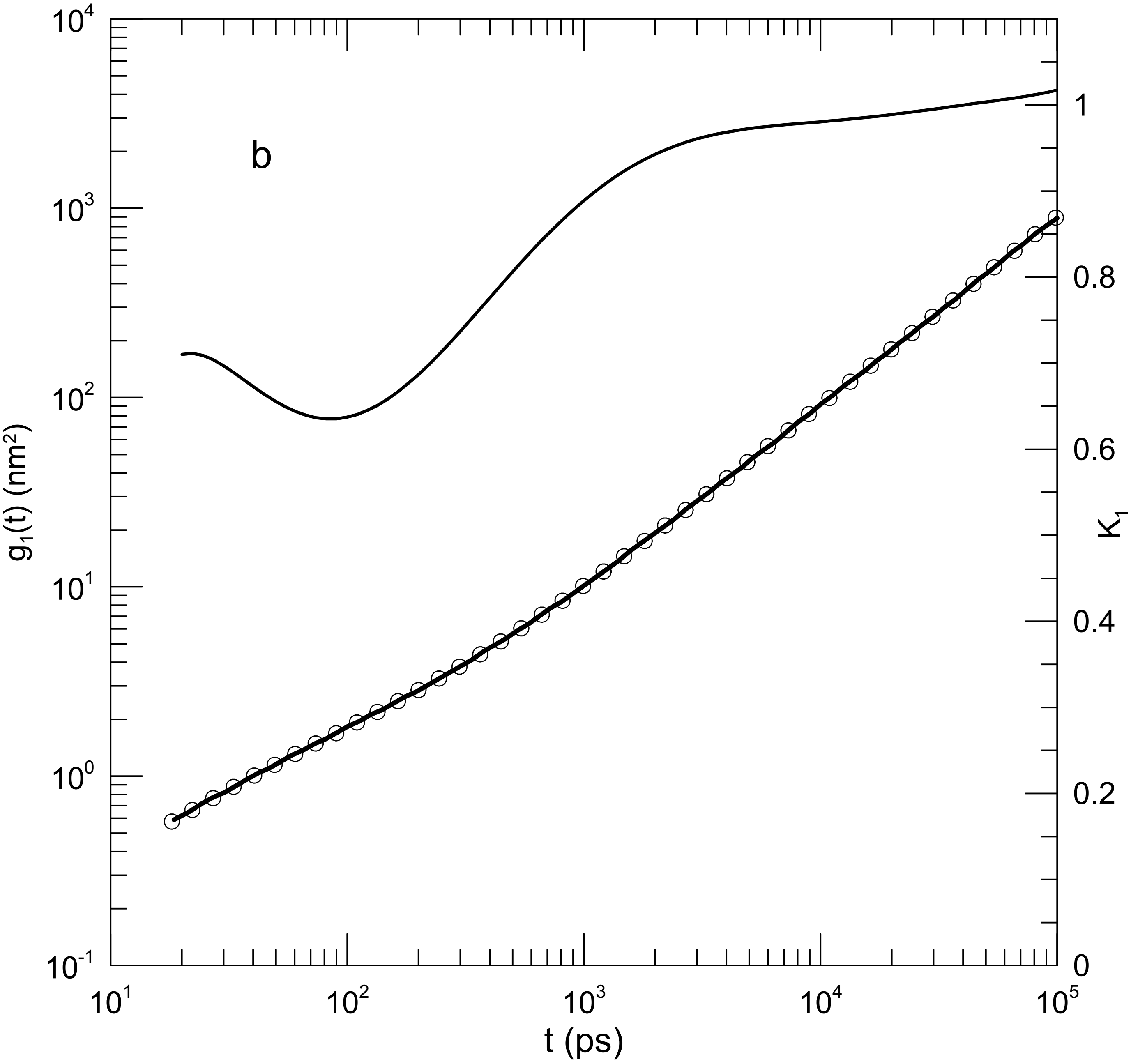

Figure S-8c. Mean-square bead displacement (thick line) of 983 Da linear polyethylenes from Takahashi, et al.[4], together with an eighth-order polynomial fit(circles) and its first logarithmic derivative K_1 (thin line).

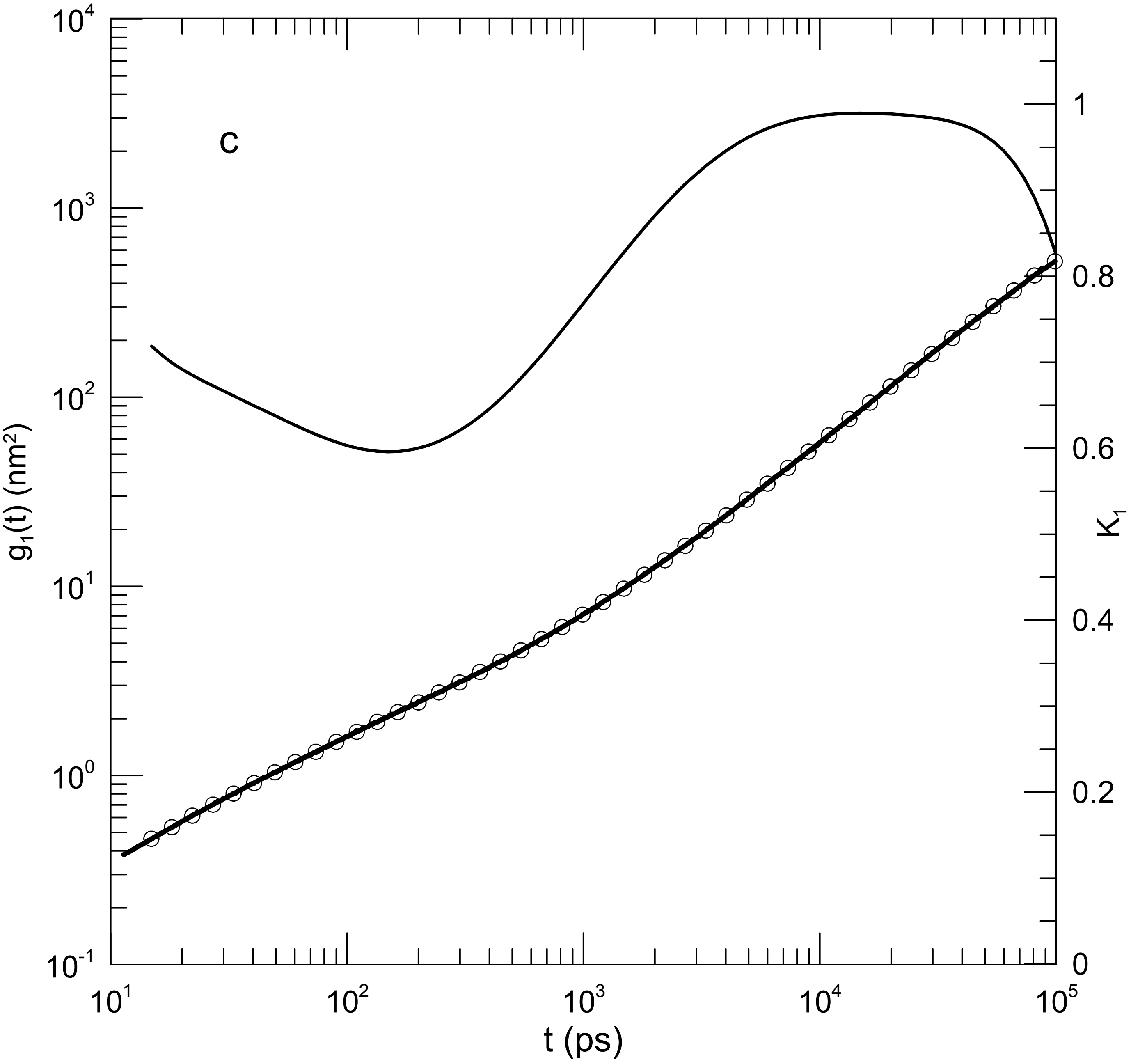

Figure S-8d. Mean-square bead displacement (thick line) of 1405 Da linear polyethylenes from Takahashi, et al.[4], together with an eighth-order polynomial fit(circles) and its first logarithmic derivative K_1 (thin line).

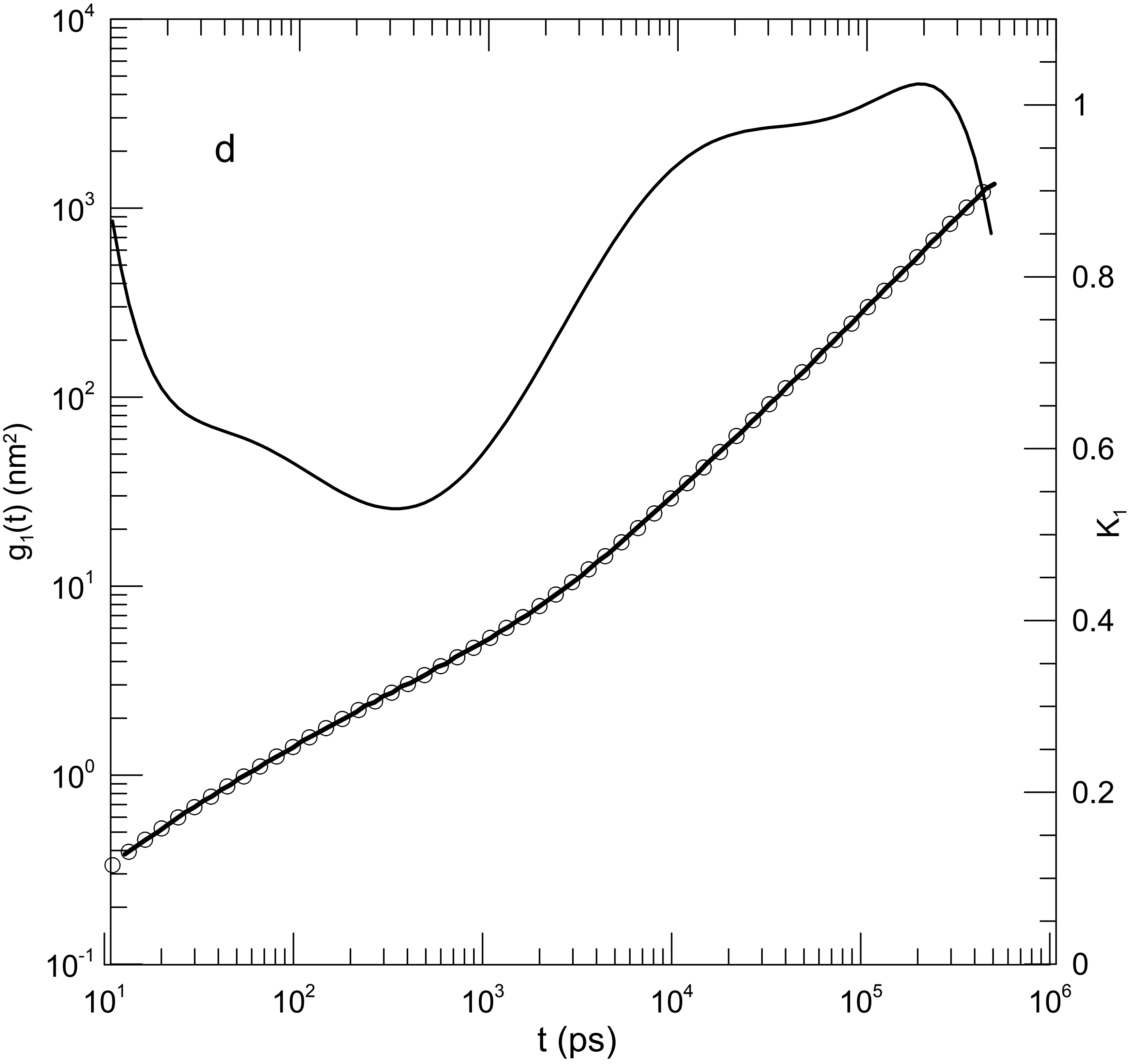

Figure S-8e. Mean-square bead displacement (thick line) of 2106 Da linear polyethylenes from Takahashi, et al.[4], together with an eighth-order polynomial fit(circles) and its first logarithmic derivative K_1 (thin line).

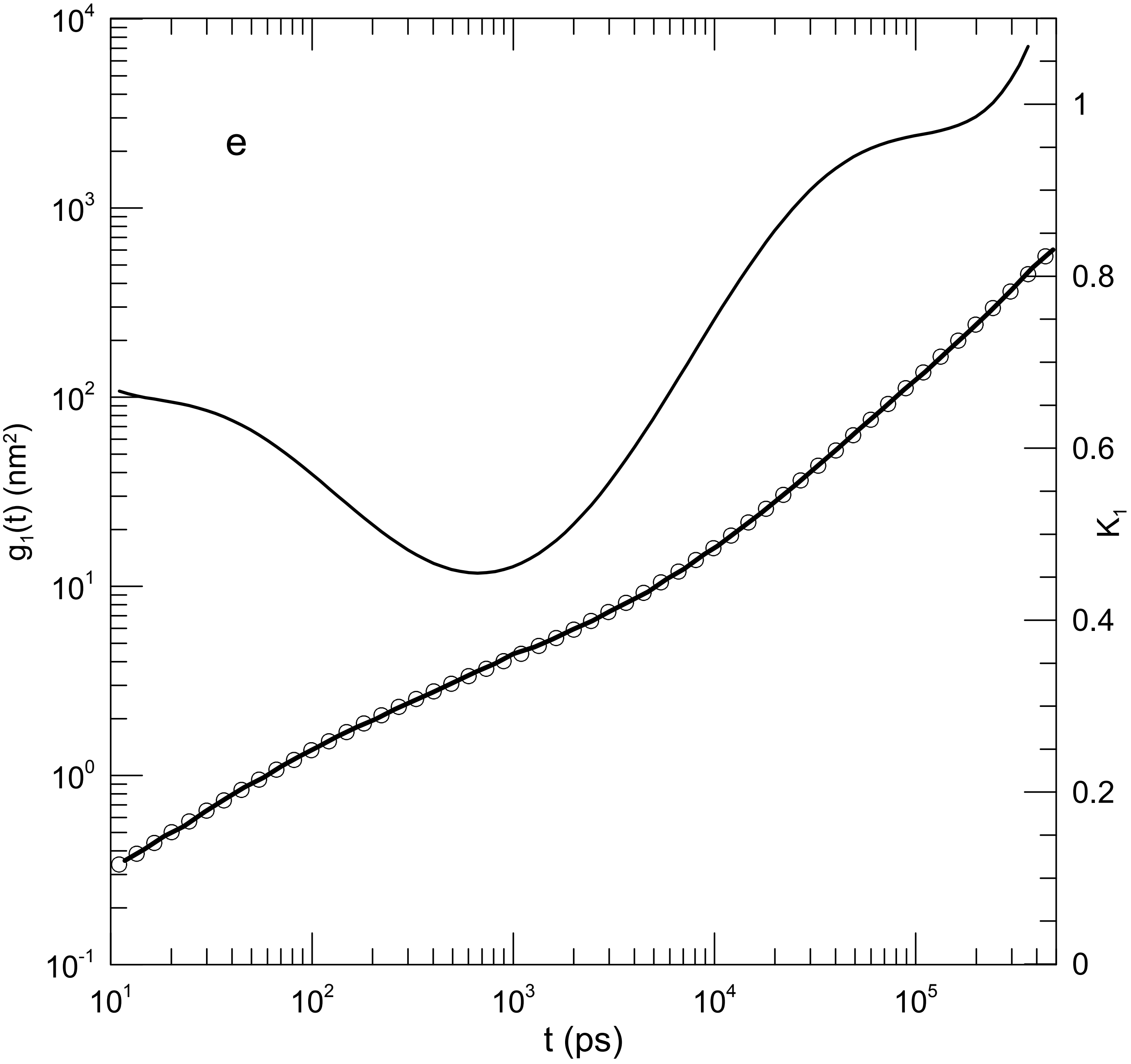

Figure S-8f. Mean-square bead displacement (thick line) of 2807 Da linear polyethylenes from Takahashi, et al.[4], together with an eighth-order polynomial fit(circles) and its first logarithmic derivative K_1 (thin line).

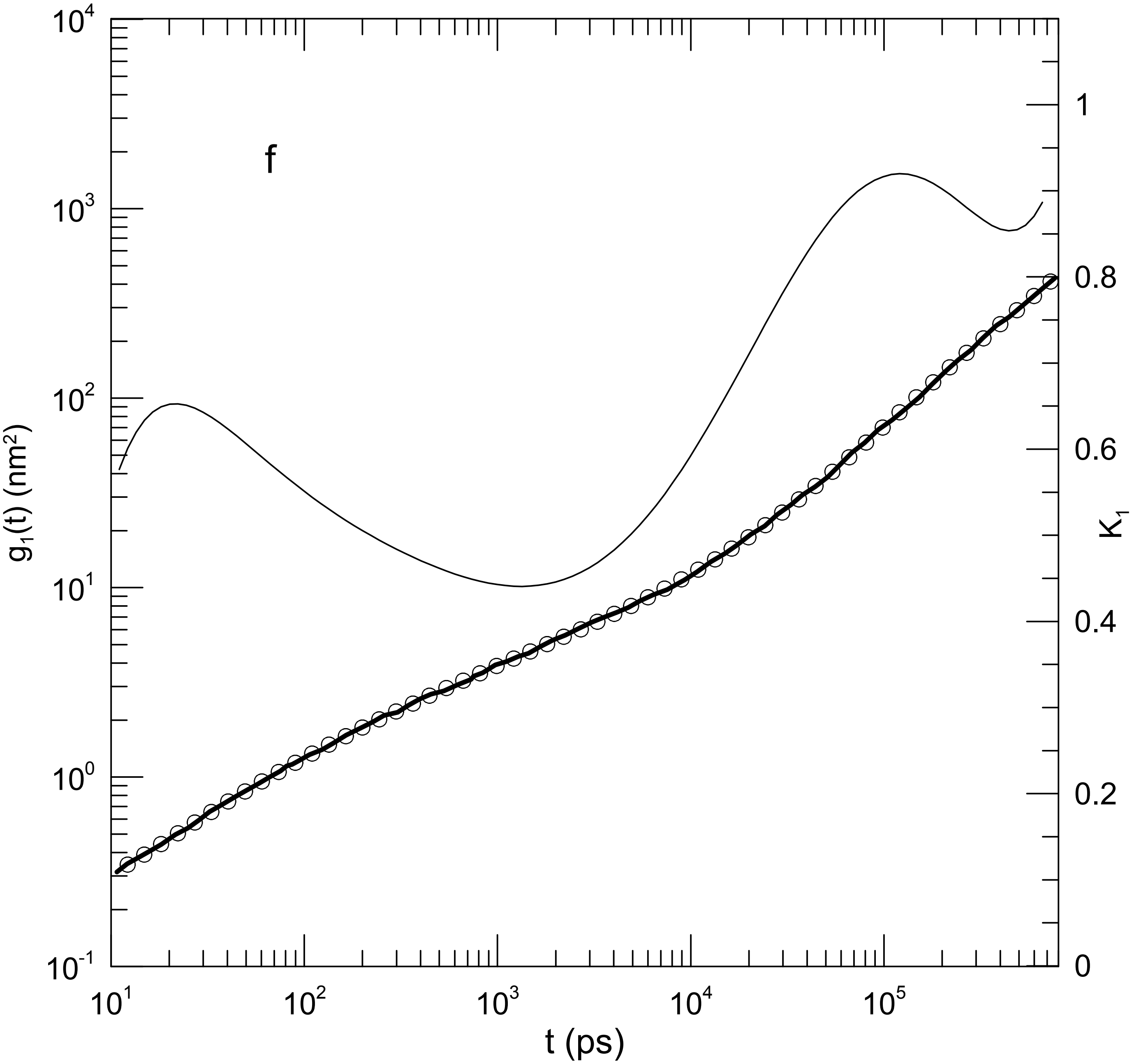

Figure S-9a. Mean-square bead displacements (thick line) of B beads in a non-phase-separated A-B mixture, from Peng, et al.[5], together with polynomial fits (circles) and first logarithmic derivatives K_1 (thin line). The A chains contained 5 beads.

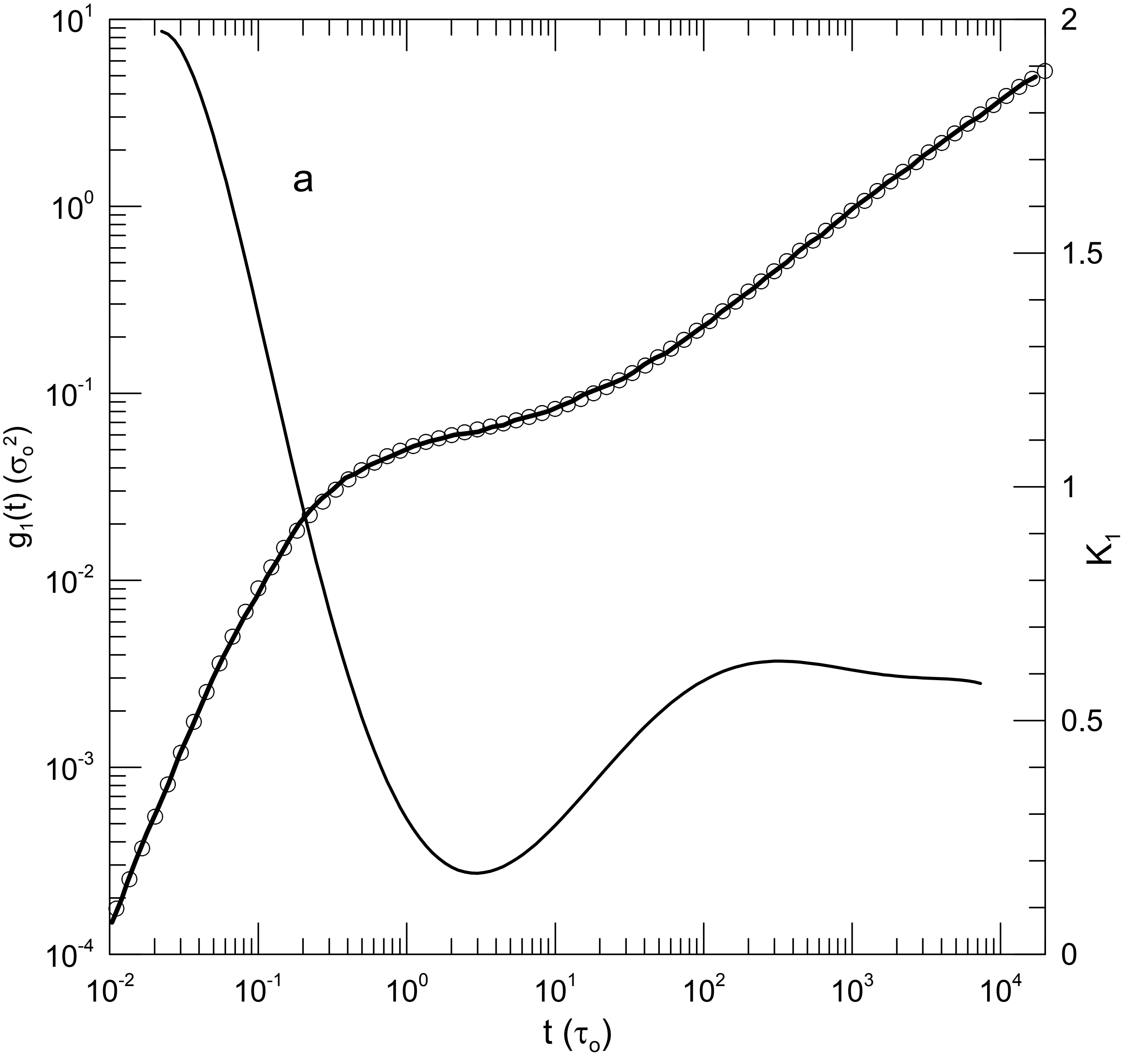

Figure S-9b. Mean-square bead displacements (thick line) of B beads in a non-phase-separated A-B mixture, from Peng, et al.[5], together with polynomial fits (circles) and first logarithmic derivatives K_1 (thin line). The A chains The A chains contained 20 beads.

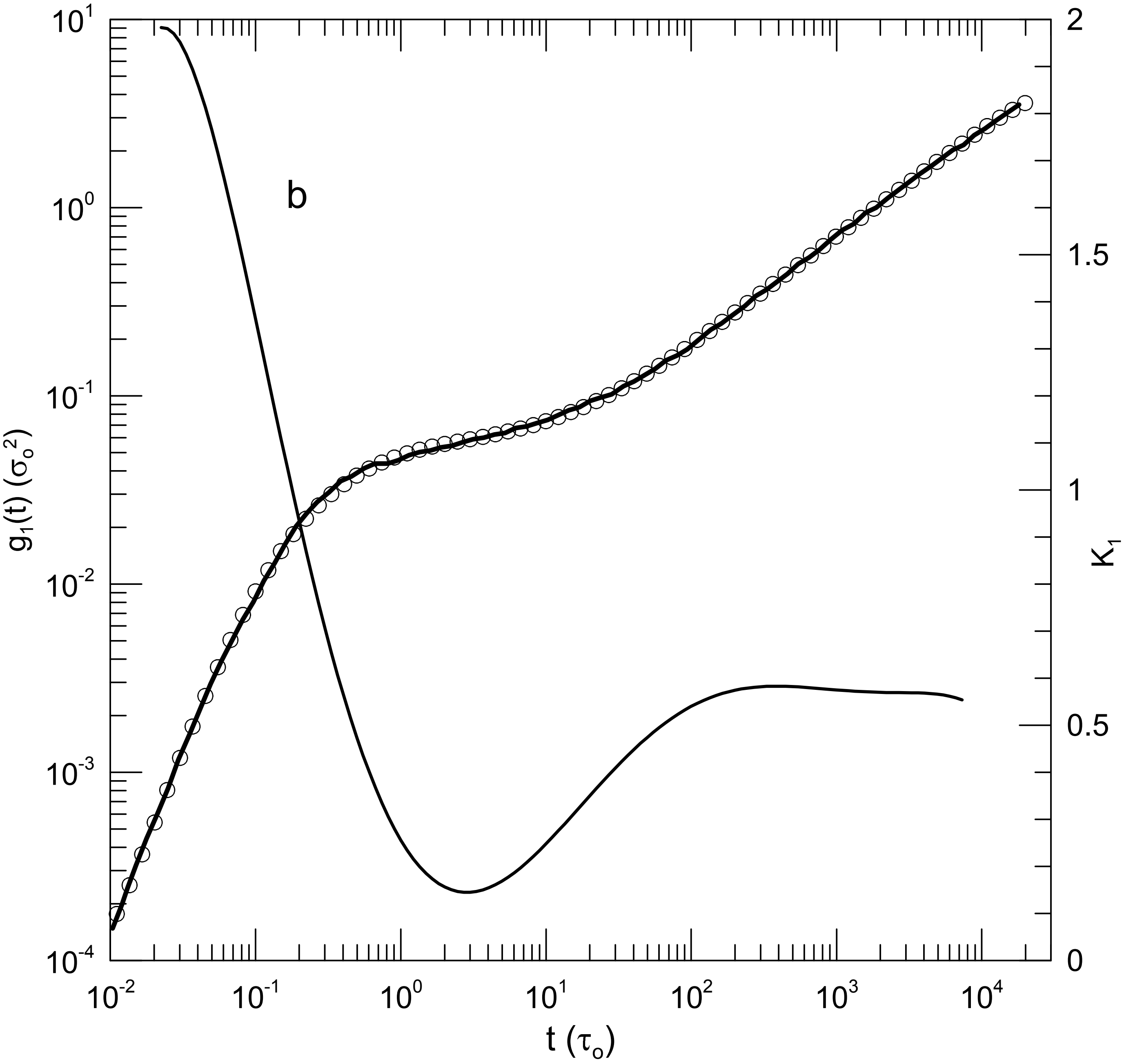

Figure S-9c. Mean-square bead displacements (thick line) of B beads in the pure-B melt from Peng, et al.[5], together with polynomial fits (circles) and first logarithmic derivatives K_1 (thin line).

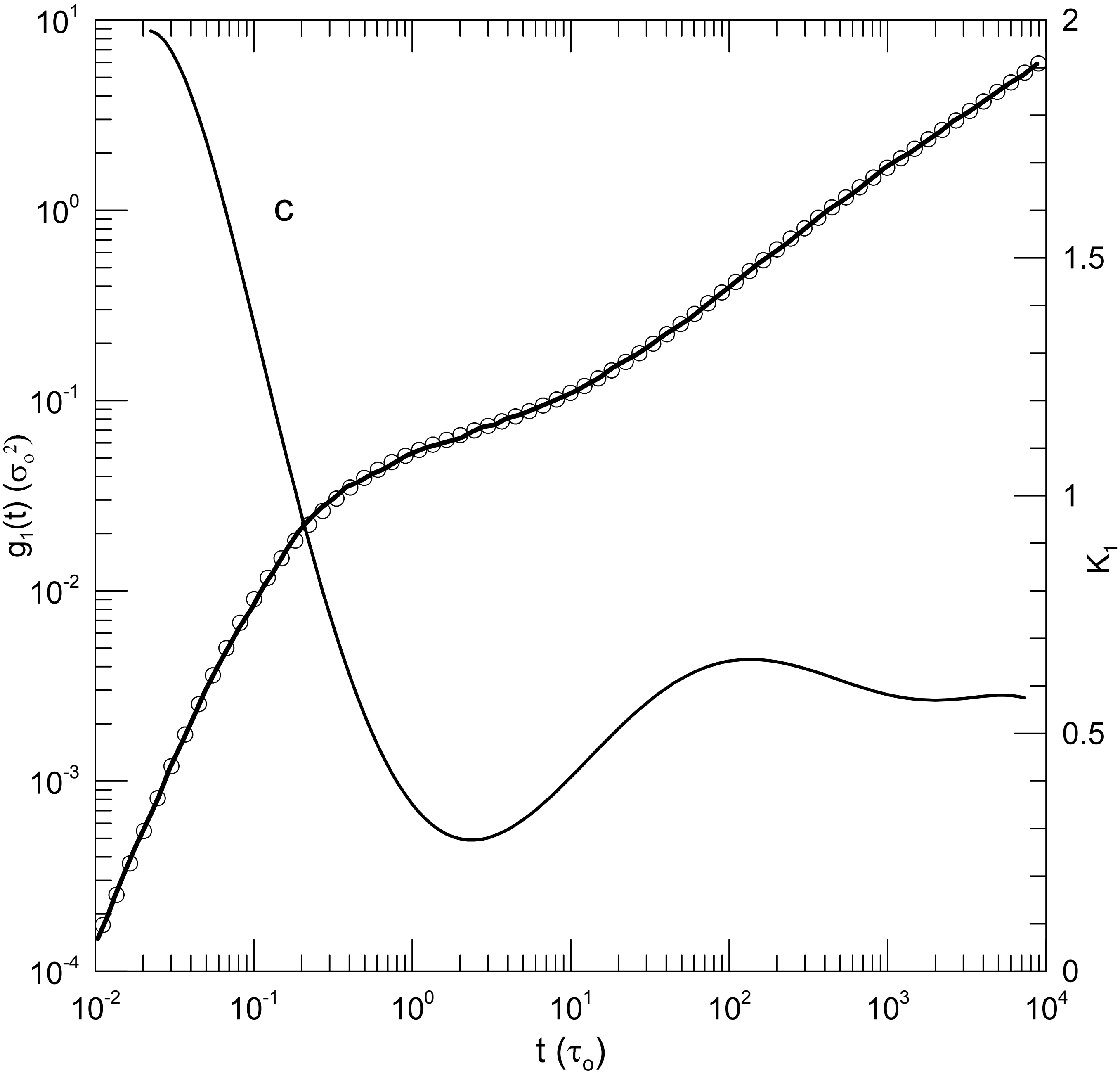

Figure S-9d. Mean-square bead displacements (thick line) of B beads in a phase-separated mixture, from Peng, et al.[5], together with polynomial fits (circles) and first logarithmic derivatives K_1 (thin line). The A chains contained 50 beads.

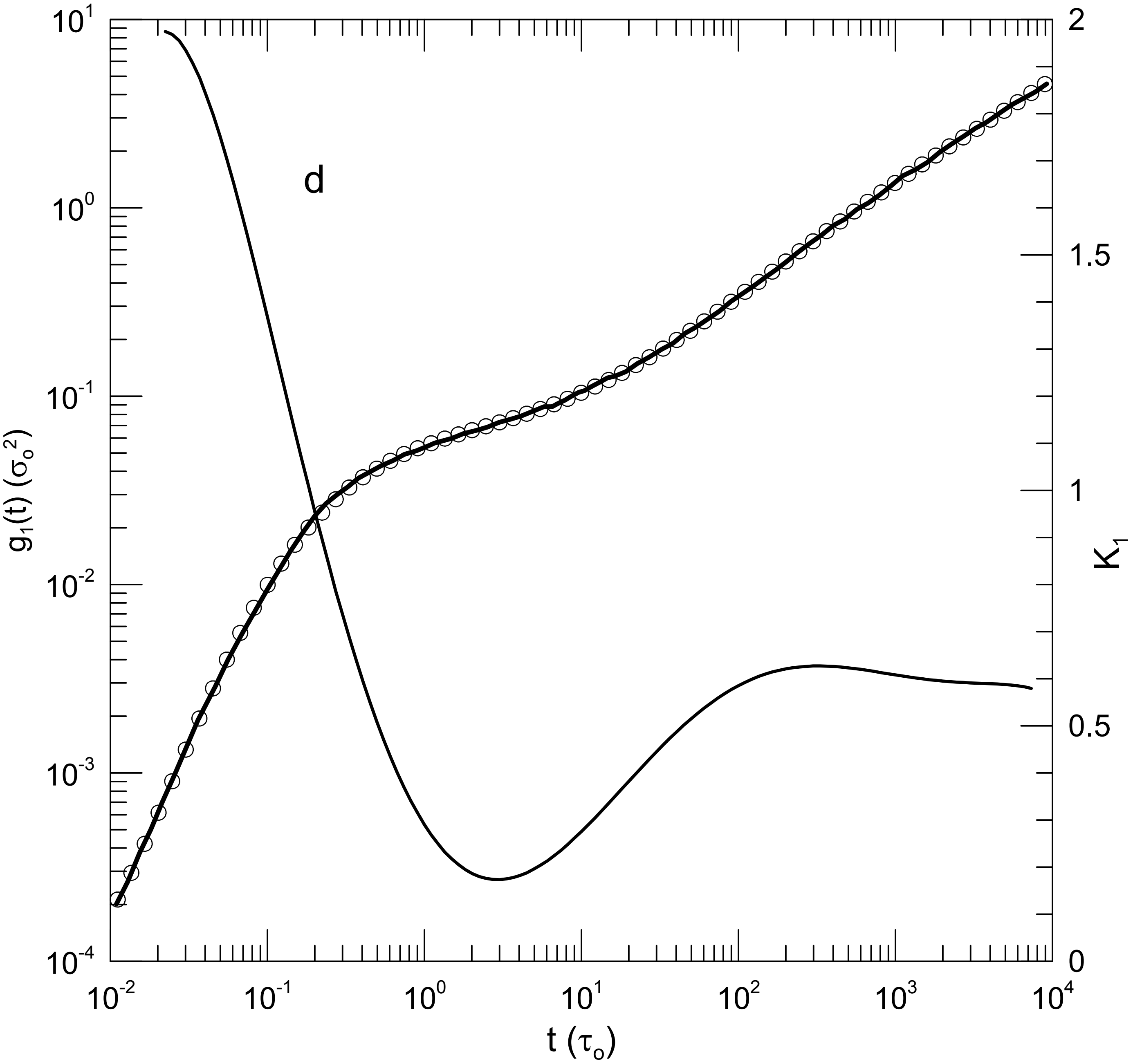

Figure S-10a. Hsu and Kremer's results for 500-bead chains[6]. Graphs show mean-square motions of individual beads. The heavy line is the simulation, circles are the polynomial fit, and the thin solid line represents the first derivative K_1.

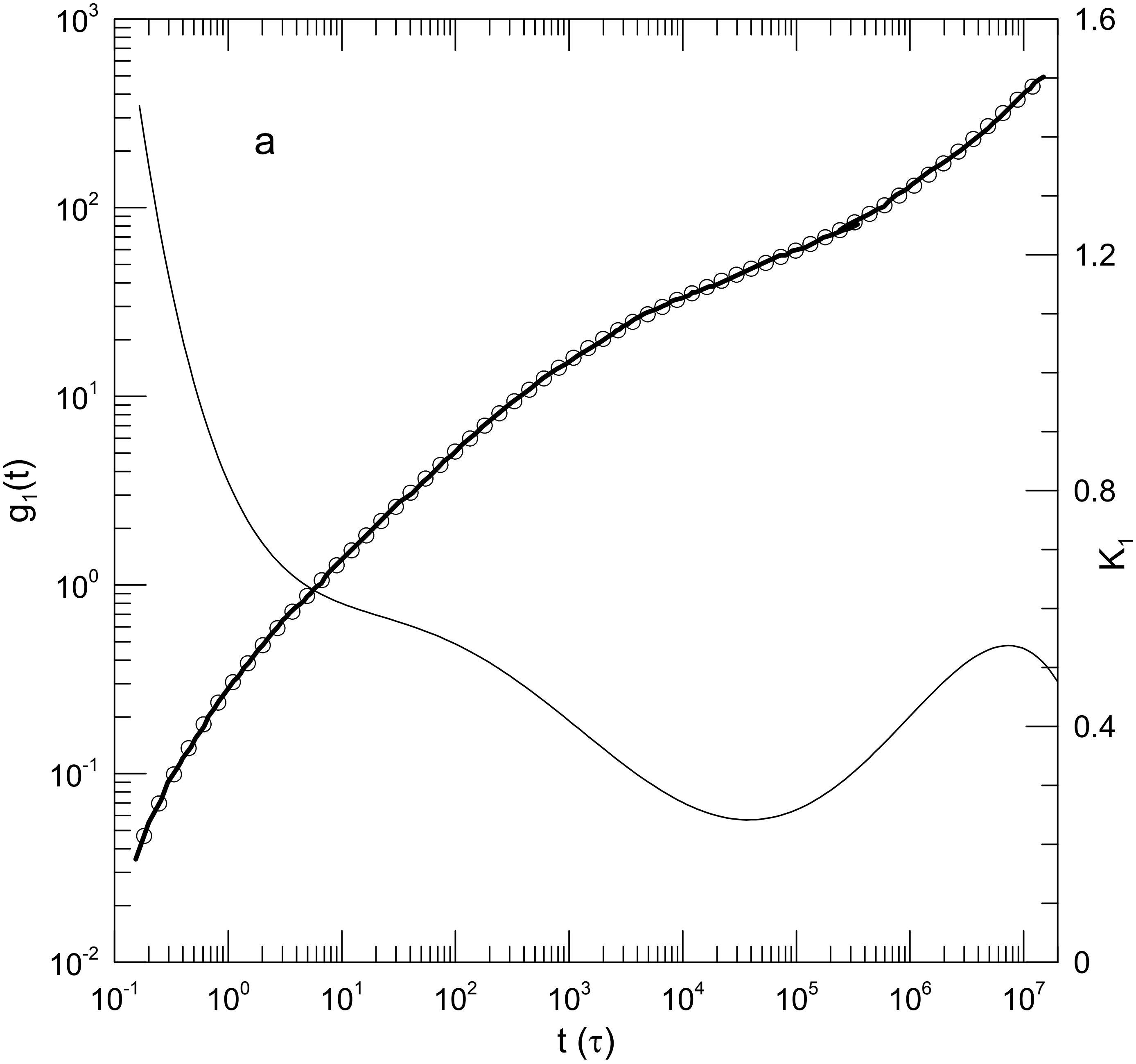

Figure S-10b. Hsu and Kremer's results for 500-bead[6]. Graphs show mean-square bead motions relative to chain centers of mass. The heavy line is the simulation, circles are the polynomial fit, and the thin solid line represents the first derivatives K_1.

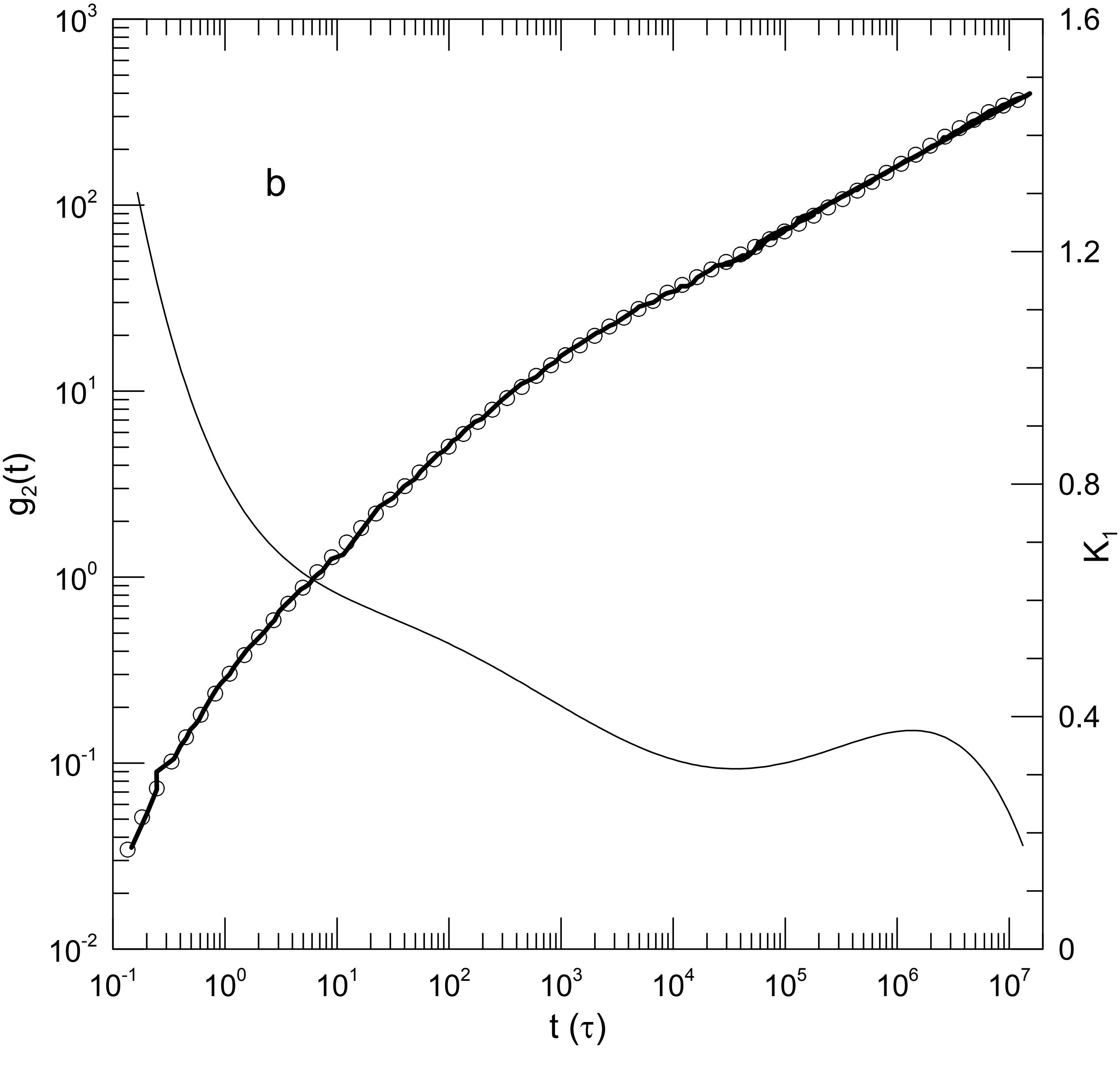

Figure S-10c. Hsu and Kremer's results for (a-c) 500-bead and (d-f) 2000-bead chains[6]. Graphs show mean-square motions of chain centers of mass. The heavy line is the simulation, circles are the polynomial fit, and the thin solid line represents the first derivative K_1.

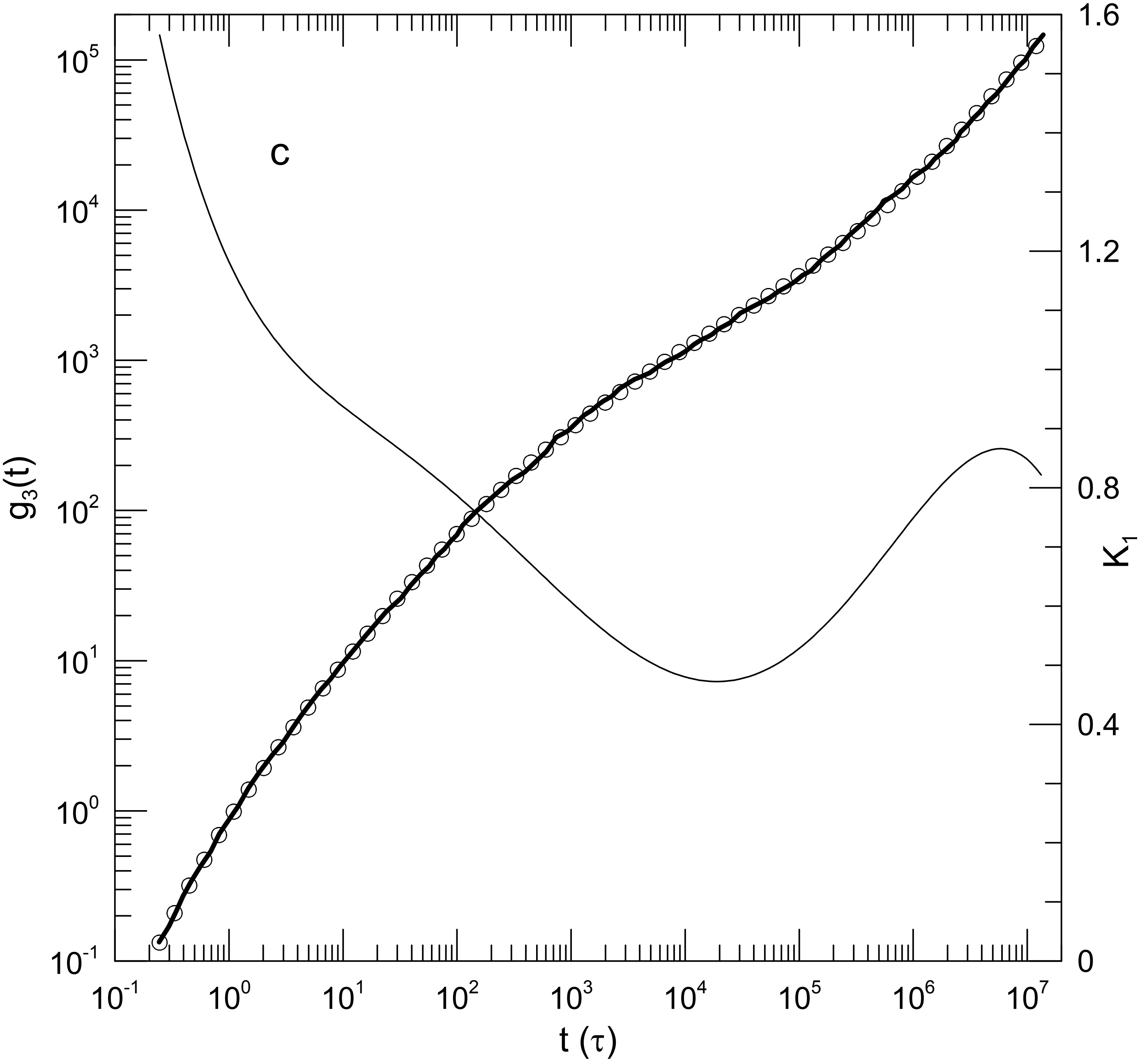

Figure S-10d. Hsu and Kremer's results for 2000-bead chains[6]. Graphs show mean-square motions of individual beads. The heavy line is the simulation, circles are the polynomial fit, and the thin solid line represents the first derivative K_1.

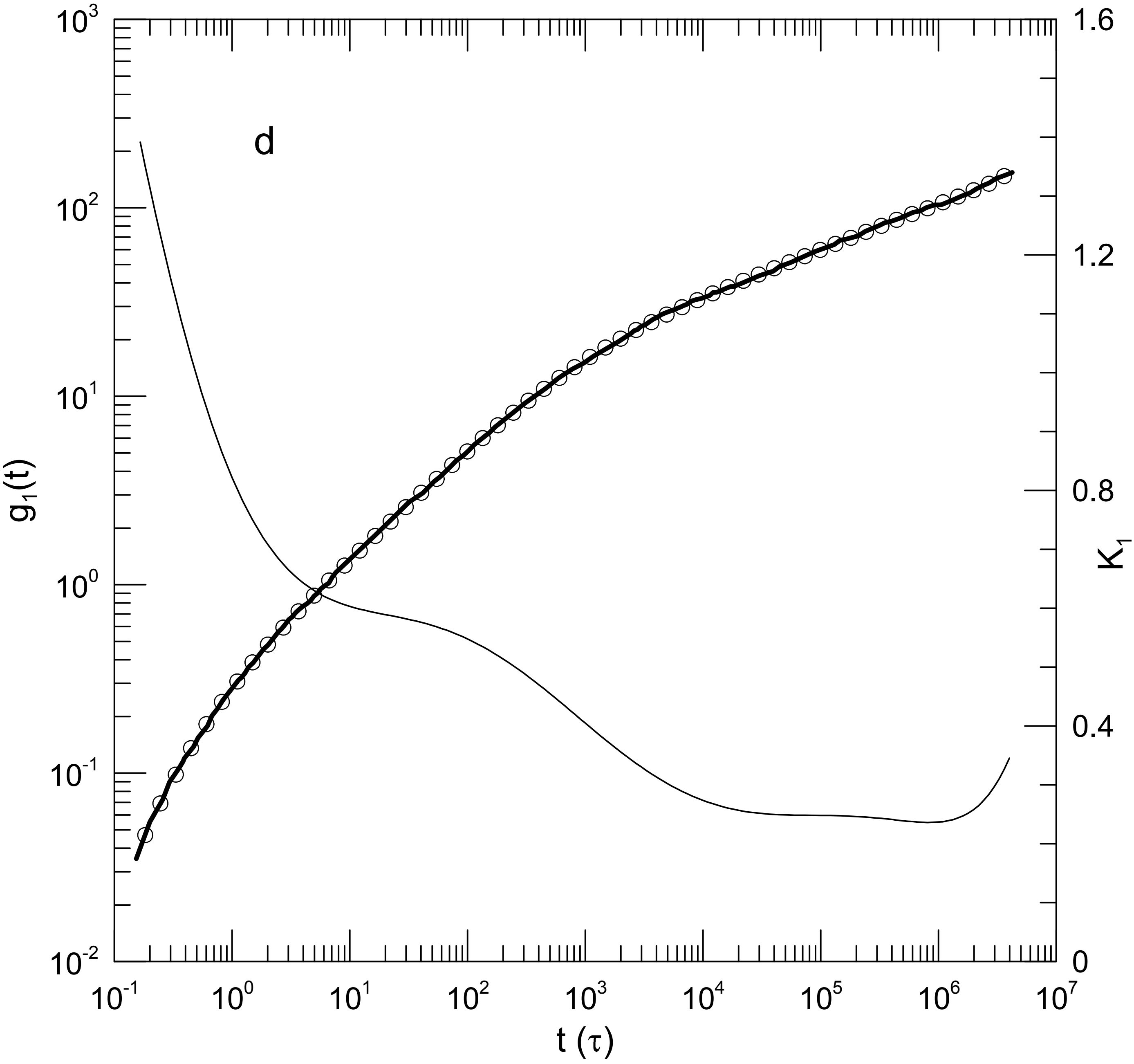

Figure S-10e. Hsu and Kremer's results for 2000-bead chains[6]. Graphs show mean-square motions of beads relative to chain centers of mass. The heavy line is the simulation, circles are the polynomial fit, and the thin solid line represents the first derivative K_1.

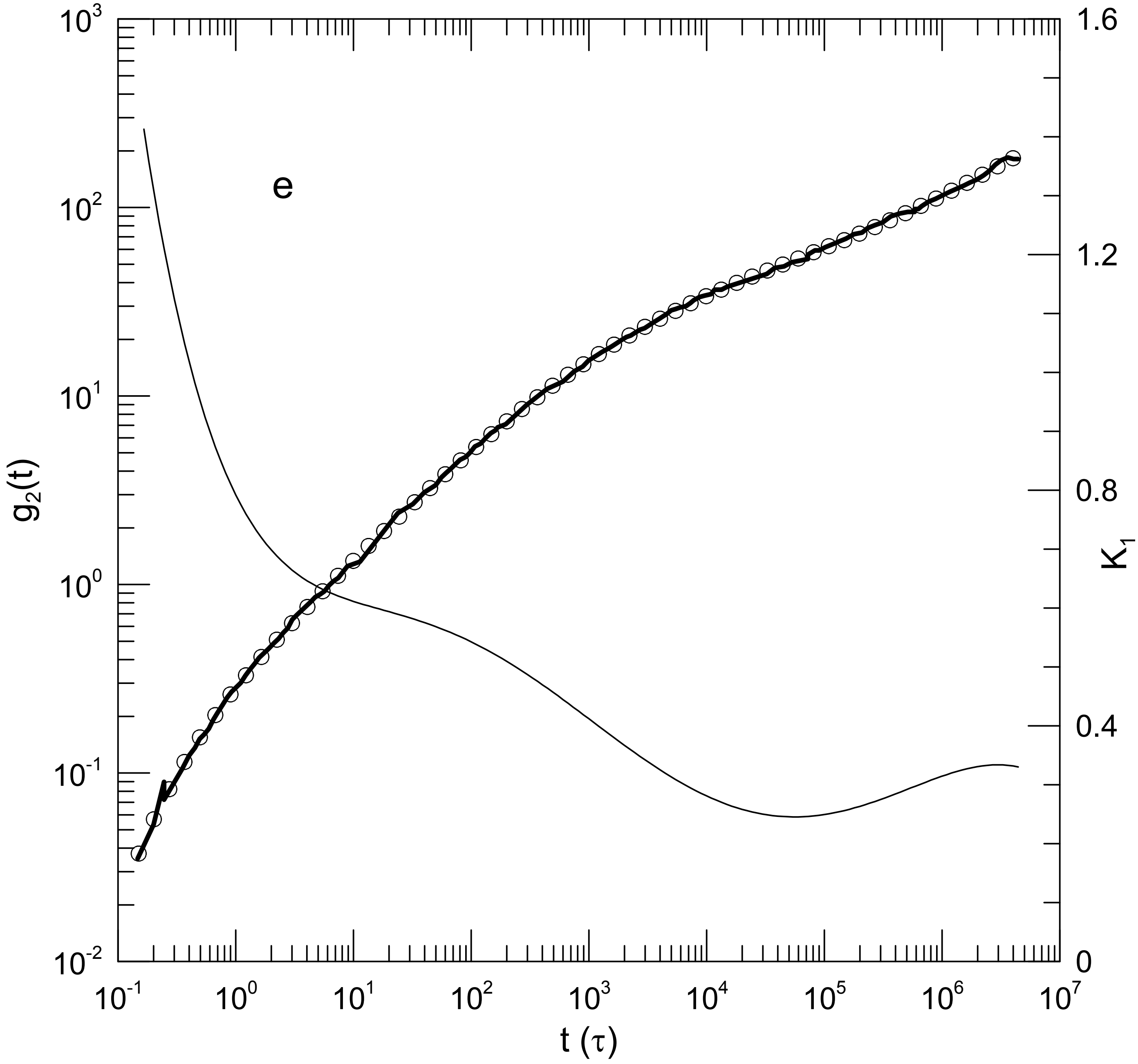

Figure S-10f. Hsu and Kremer's results for 2000-bead chains[6]. Graphs show mean-square motions of the chain centers of mass. The heavy line is the simulation, circles are the polynomial fit, and the thin solid line represents the first derivative K_1.

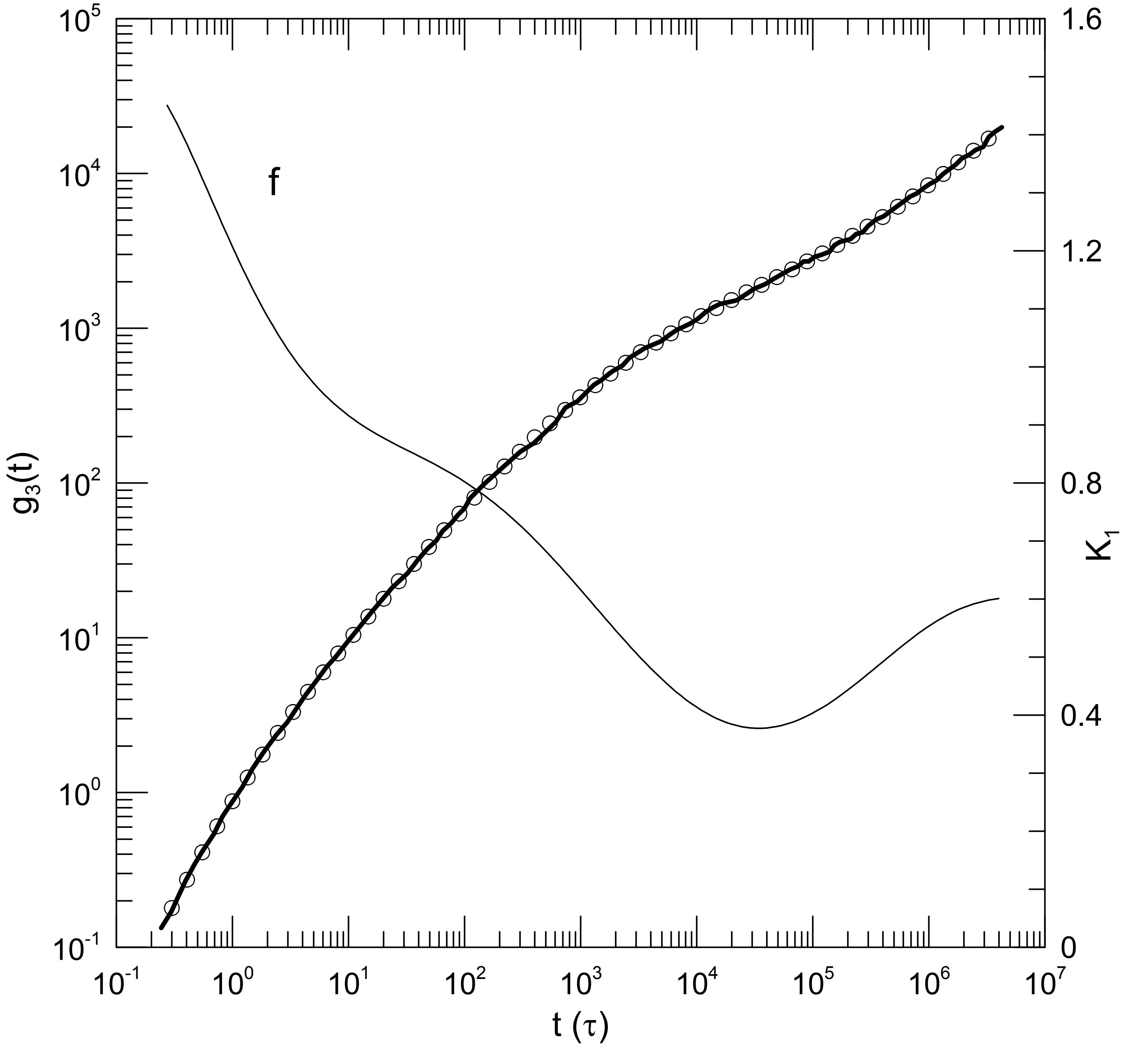

Figure S-11a. Mean-square displacement $g_1(t)$ of individual PEO hydrogen atoms in PEO-PMMA blend melts at temperature 500K from Brodeck, et al.[7]. Heavy line indicates Brodeck, et al.,'s results, circles are the eighth-order polynomial fit, and the thin solid line represents the first logarithmic derivative K_1.

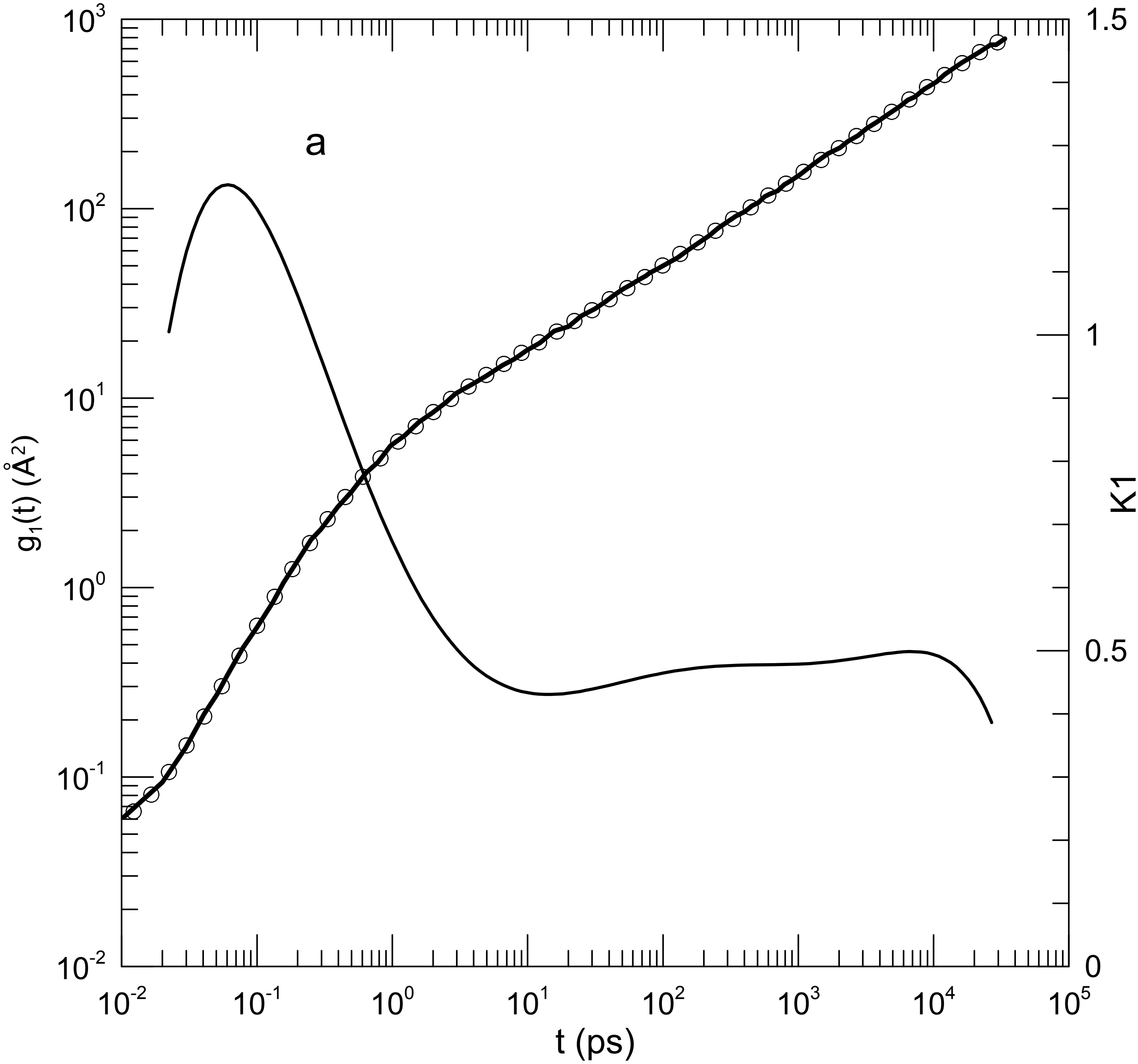

Figure S-11b. Mean-square displacement $g_1(t)$ of individual PEO hydrogen atoms in PEO-PMMA blend melts at temperature 400K from Brodeck, et al.[7]. Heavy line indicates Brodeck, et al.,'s results, circles are the eighth-order polynomial fit, and the thin solid line represents the first logarithmic derivative K_1.

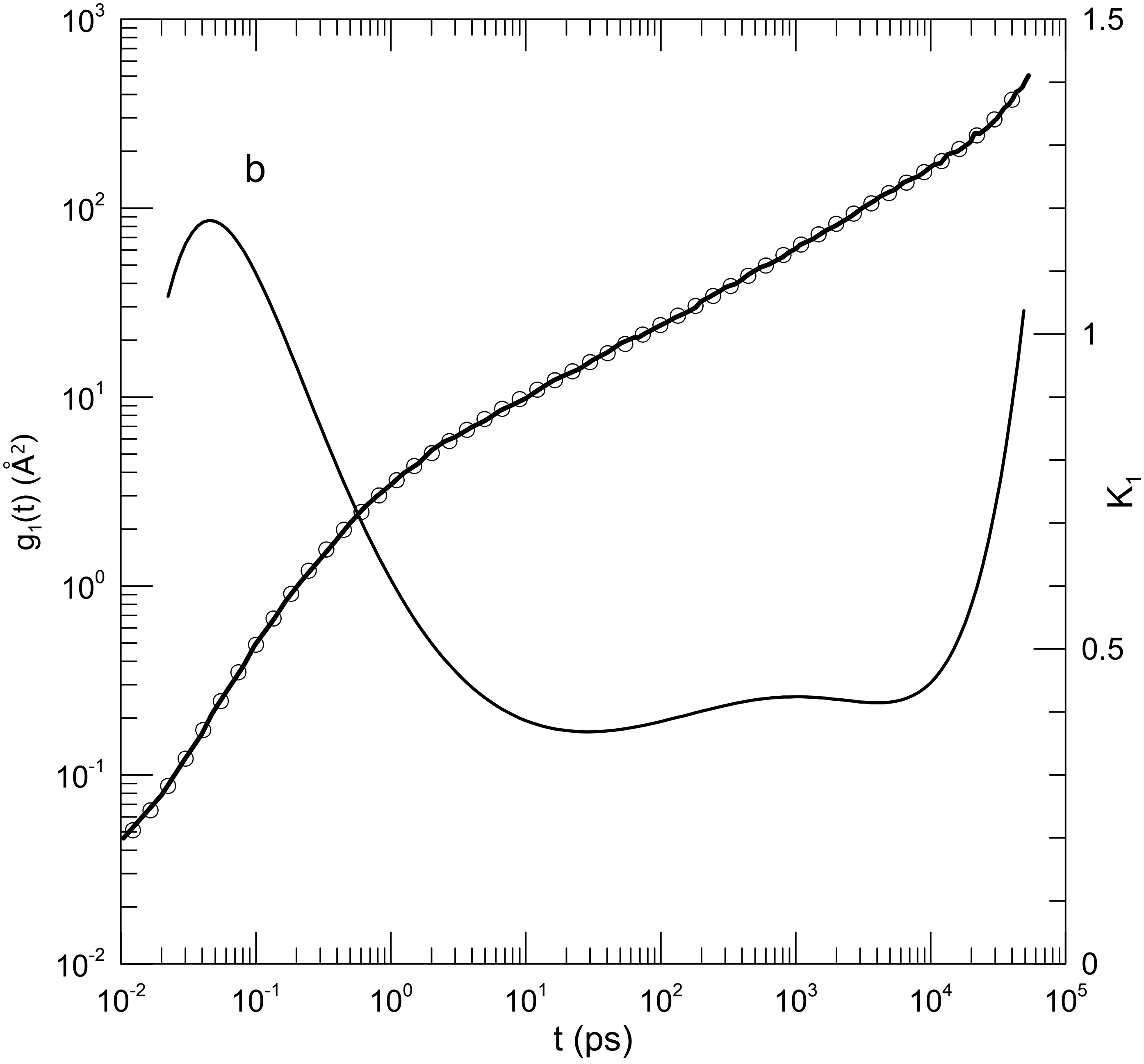

Figure S-11c. Mean-square displacement $g_1(t)$ of individual PEO hydrogen atoms in PEO-PMMA blend melts at temperature 350K from Brodeck, et al.[7]. Heavy line indicates Brodeck, et al.,'s results, circles are the eighth-order polynomial fit, and the thin solid line represents the first logarithmic derivative K_1.

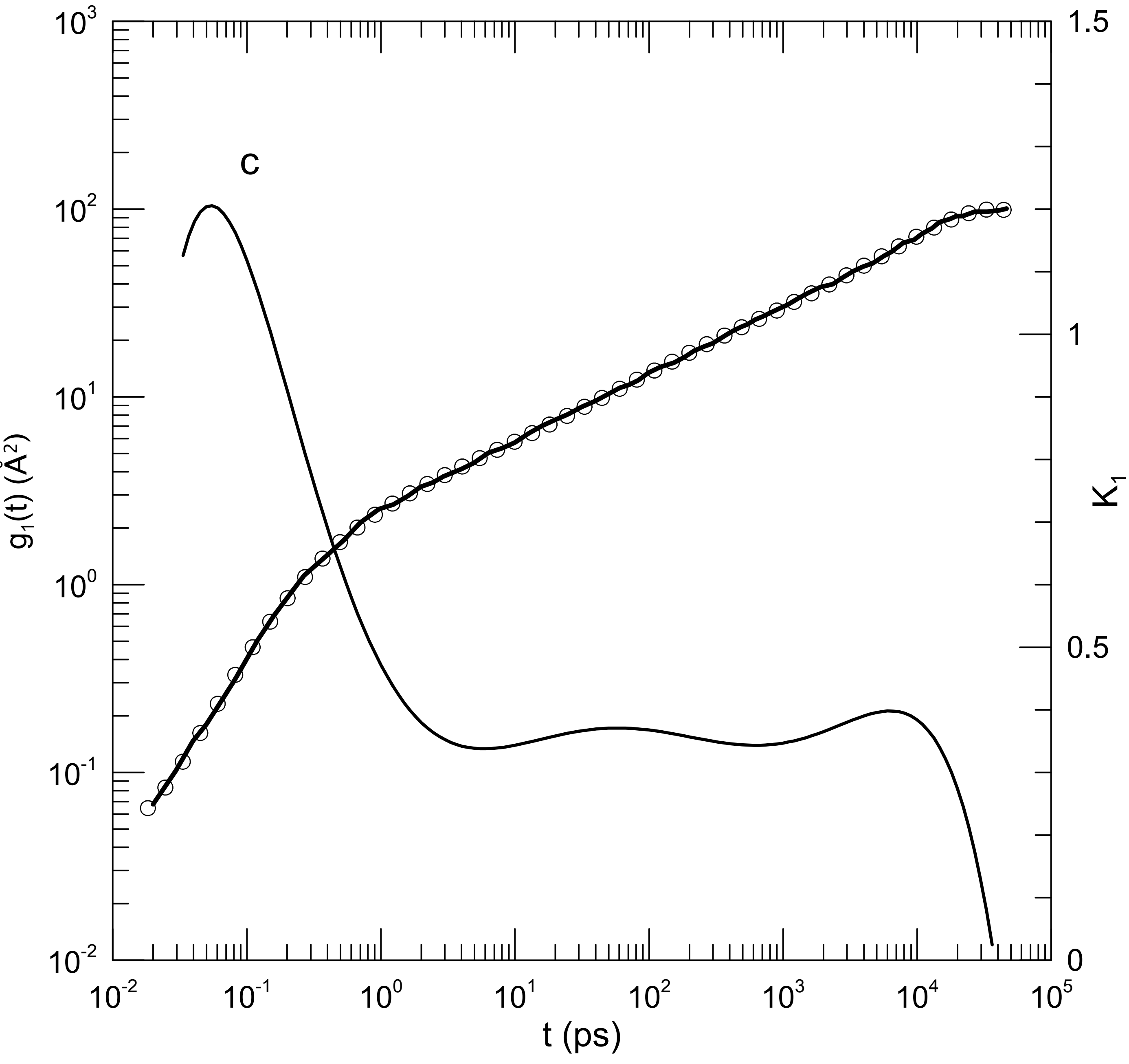

Figure S-11d. Mean-square displacement $g_1(t)$ of individual PEO hydrogen atoms in PEO-PMMA blend melts at temperature 300K from Brodeck, et al.[7]. Heavy line indicates Brodeck, et al.,'s results, circles are the eighth-order polynomial fit, and the thin solid line represents the first logarithmic derivative K_1.

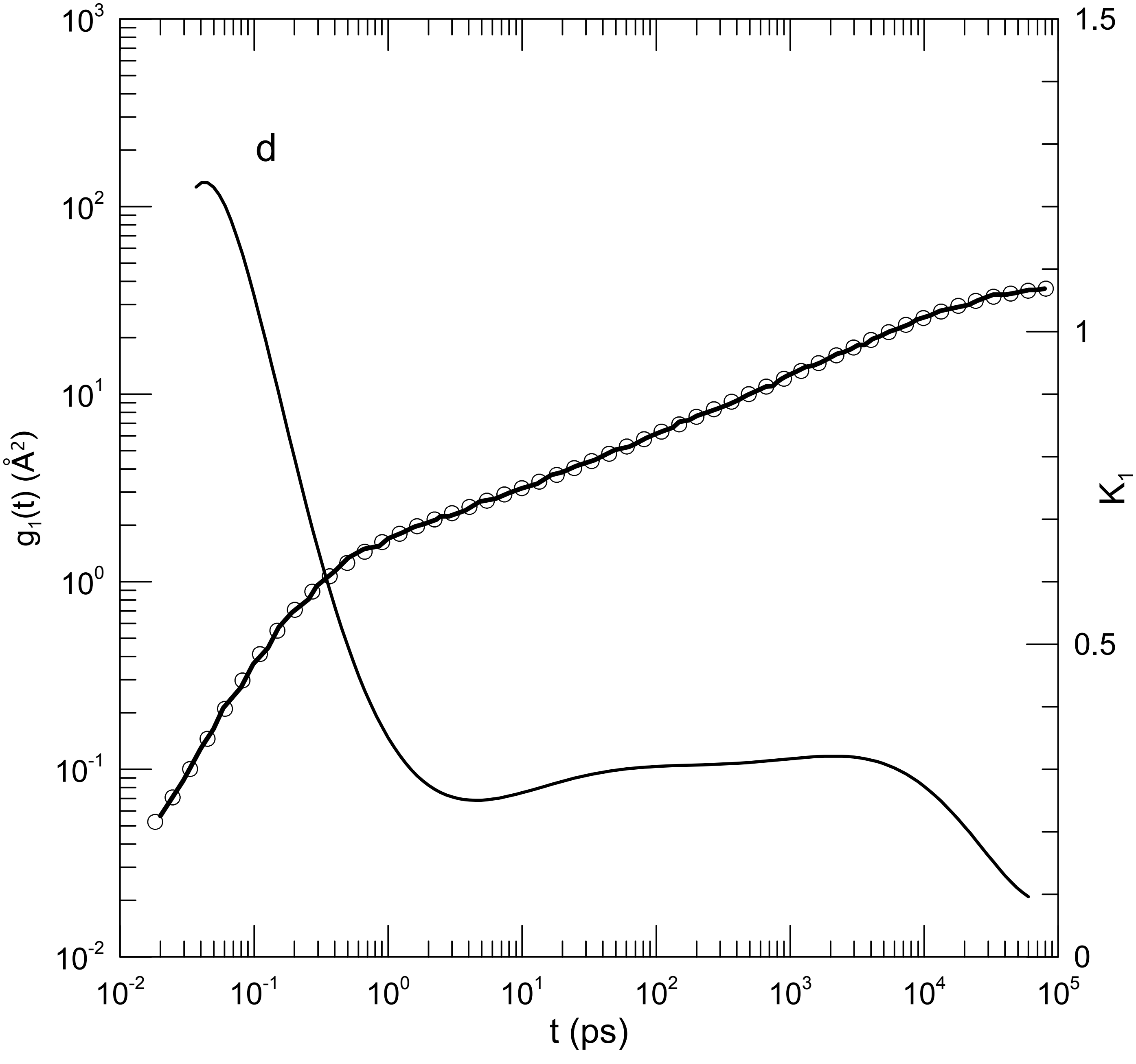

Figure S-12a. Mean-square displacement $g_3(t)$ of chain centers-of-mass of PEO molecules in PEO-PMMA blend melts at temperature 500K from Brodeck, et al.[7]. Heavy line indicates Brodeck, et al.,'s results, circles are the eighth-order polynomial fit, and the thin solid line represents the first logarithmic derivative K_1.

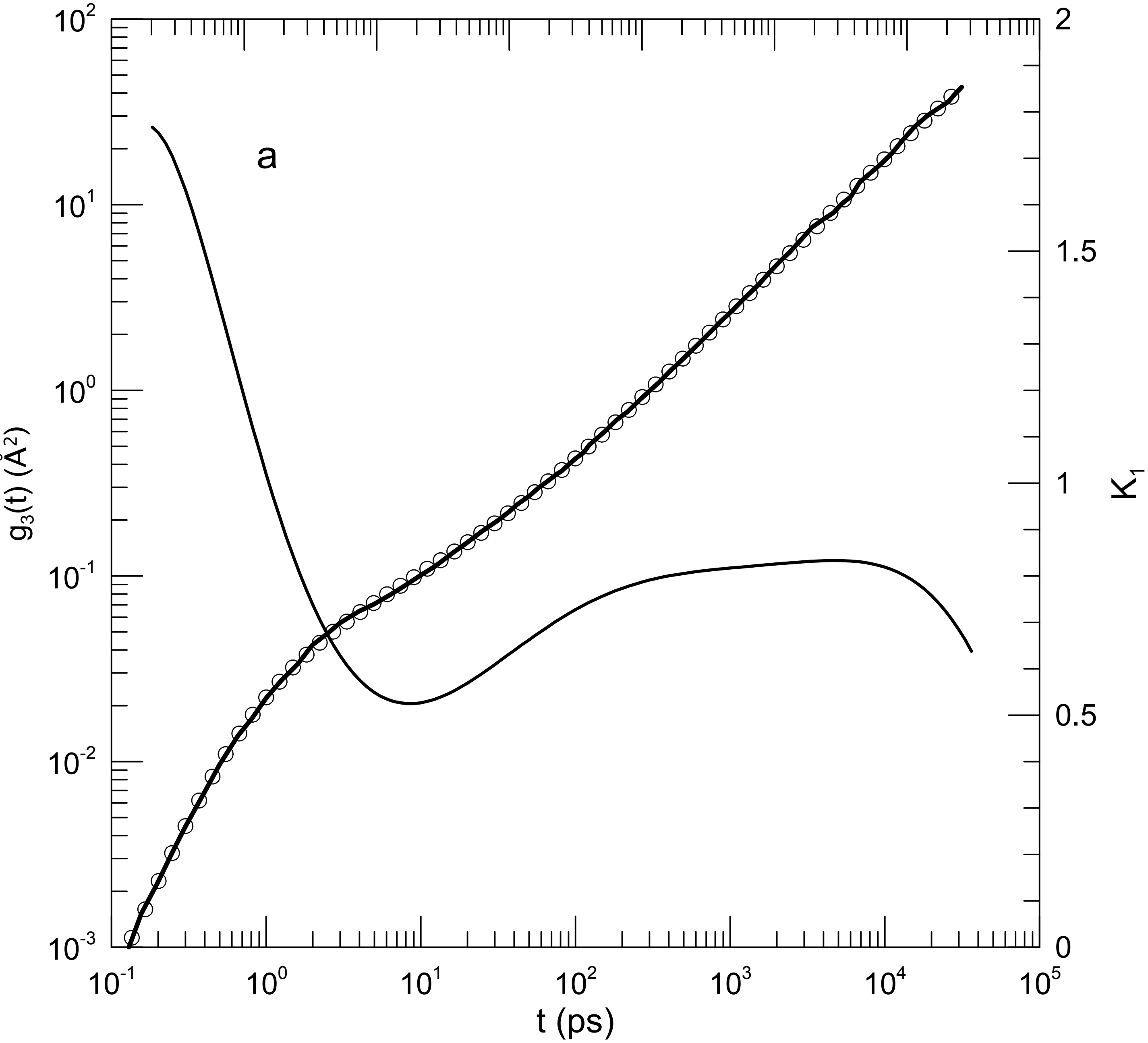

Figure S-12b. Mean-square displacement $g_3(t)$ of chain centers-of-mass of PEO molecules in PEO-PMMA blend melts at temperature 400K from Brodeck, et al.[7]. Heavy line indicates Brodeck, et al.,'s results, circles are the eighth-order polynomial fit, and the thin solid line represents the first logarithmic derivative K_1.

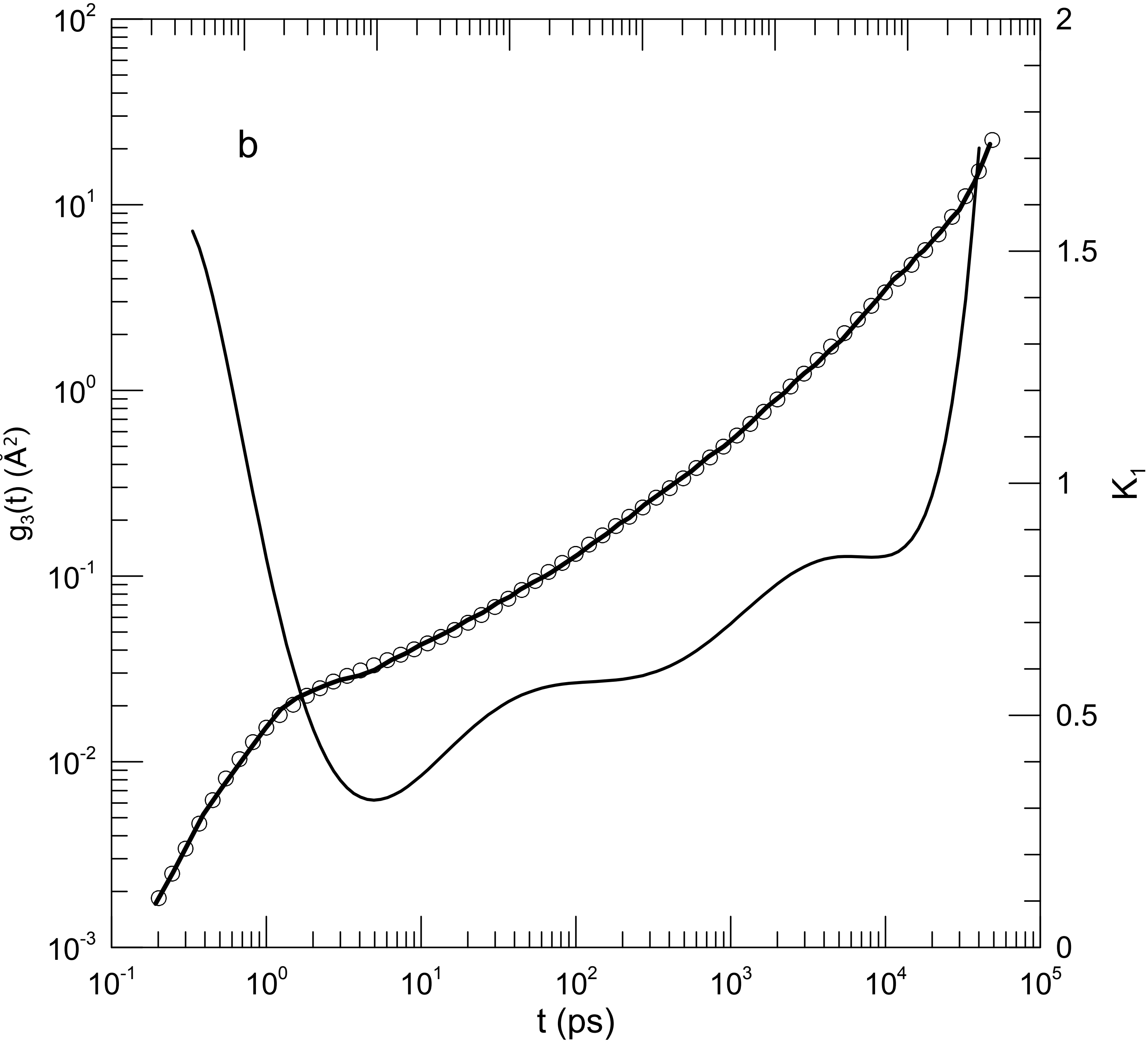

Figure S-12c. Mean-square displacement $g_3(t)$ of chain centers-of-mass of PEO molecules in PEO-PMMA blend melts at temperatures 350K from Brodeck, et al.[7]. Heavy line indicates Brodeck, et al.,'s results, circles are the eighth-order polynomial fit, and the thin solid line represents the first logarithmic derivative K_1.

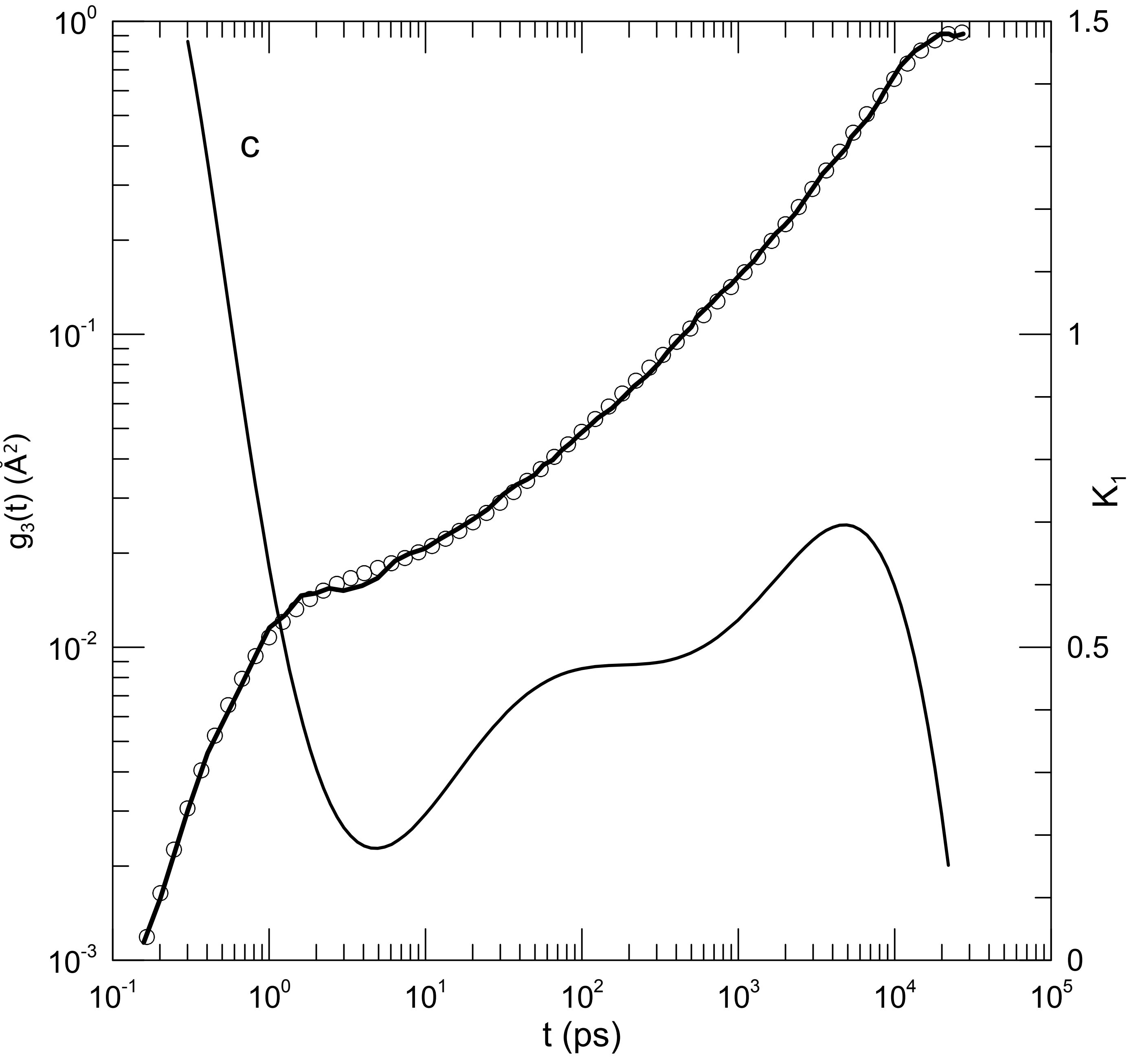

Figure S-12d. Mean-square displacement $g_3(t)$ of chain centers-of-mass of PEO molecules in PEO-PMMA blend melts at temperatures 300K from Brodeck, et al.[7]. Heavy line indicates Brodeck, et al.,'s results, circles are the eighth-order polynomial fit, and the thin solid line represents the first logarithmic derivative K_1.

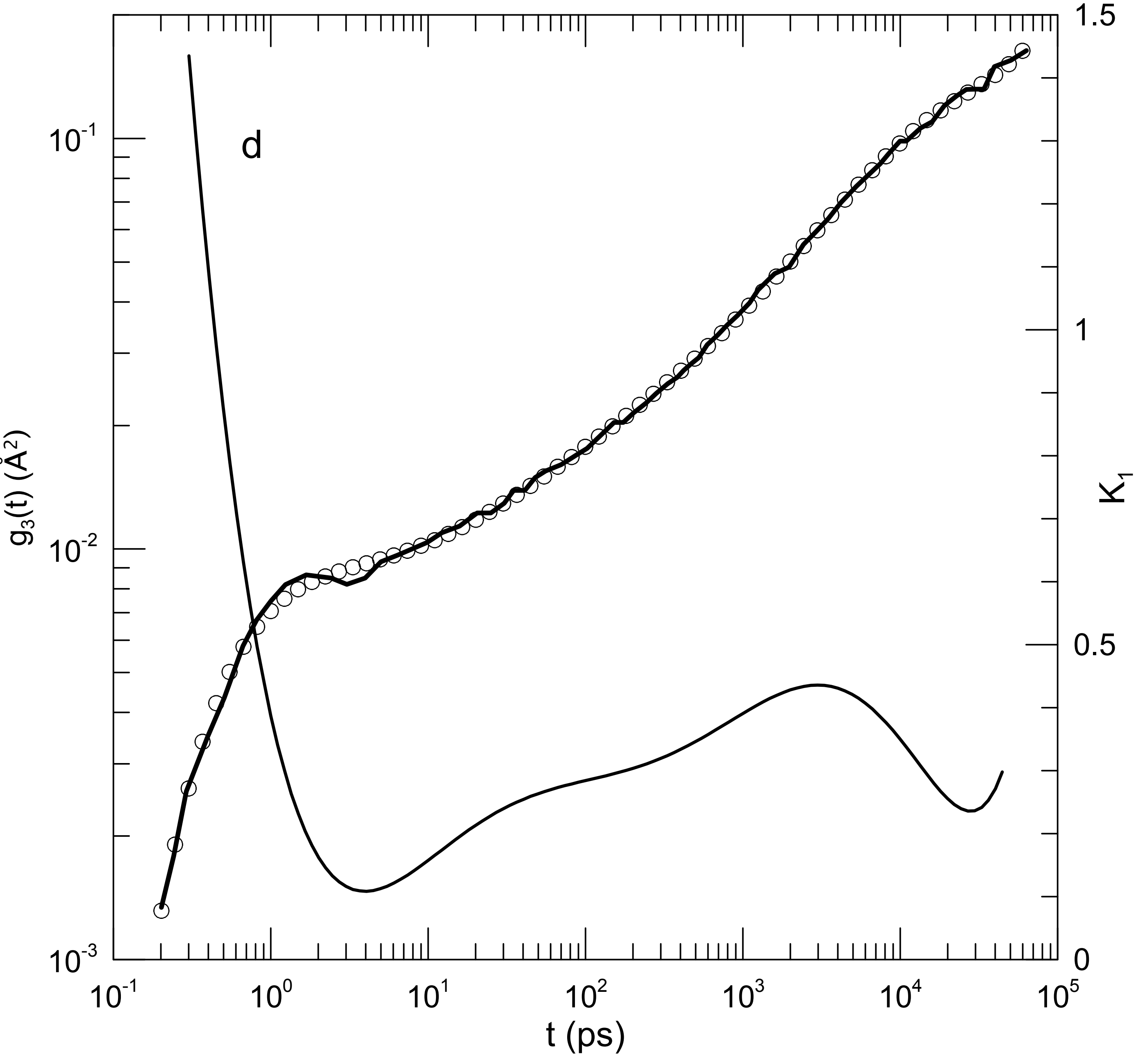

Figure S-13a. Mean-square displacement $g_1(t)$ of the central beads of a chain, and (b) mean displacement of individual primitive path segments perpendicular to the chain's primitive path, based on Stephanou, et al.[8]. Heavy line is a digitization of Stephanou, et al.'s data, circles are from an eighth-order least-mean-square polynomial fit, and the thin solid line represents the first derivative K_1 of the polynomial fit.

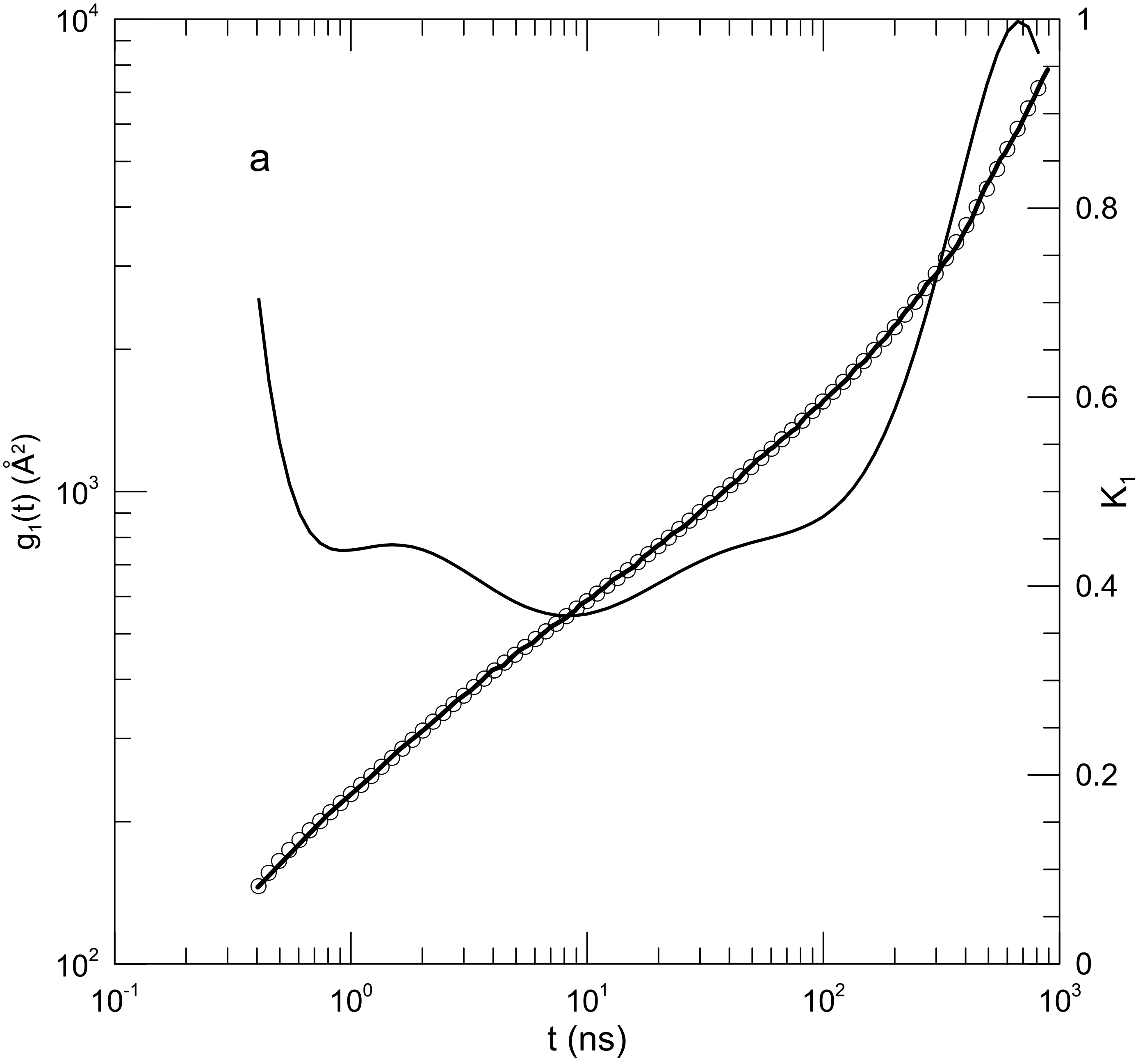

]

Figure S-13b. Mean-square displacement $g_1(t)$ of the central beads of a chain, and (b) mean displacement of individual primitive path segments perpendicular to the chain's primitive path, based on Stephanou, et al.[8]. Heavy line is a digitization of Stephanou, et al.'s data, circles are from an eighth-order least-mean-square polynomial fit, and the thin solid line represents the first derivative K_1 of the polynomial fit.

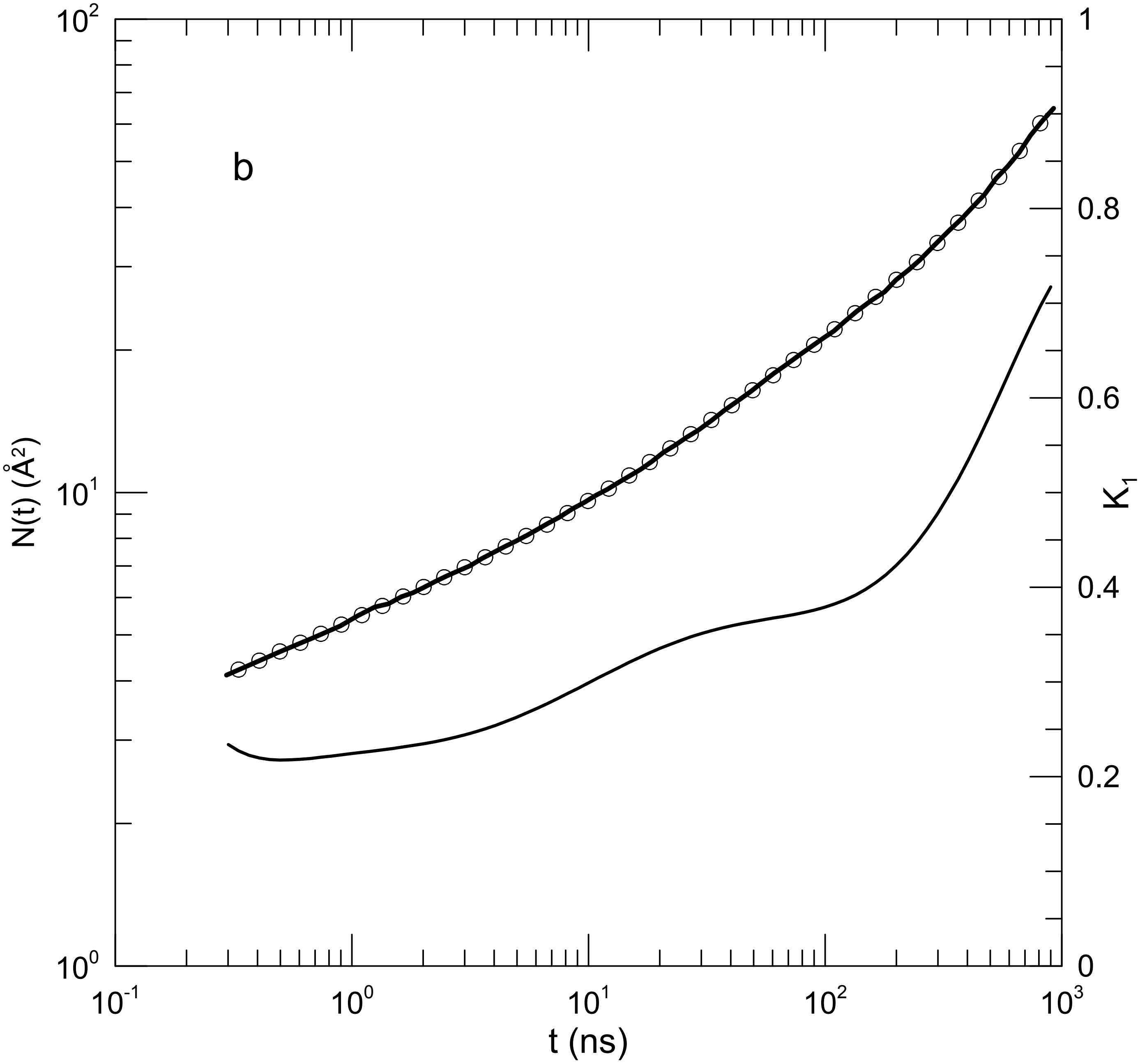

Figure S-14a Mean-square displacements of central beads of 50-bead simulated Grest-Kremer polymer melts, based on Likhtman, et al.[9]. The heavy line represents Likhtman, et al.,'s data, circles show the eighth-order polynomial fit, and the thin solid line is the first derivative K_1 of the polynomial fit.

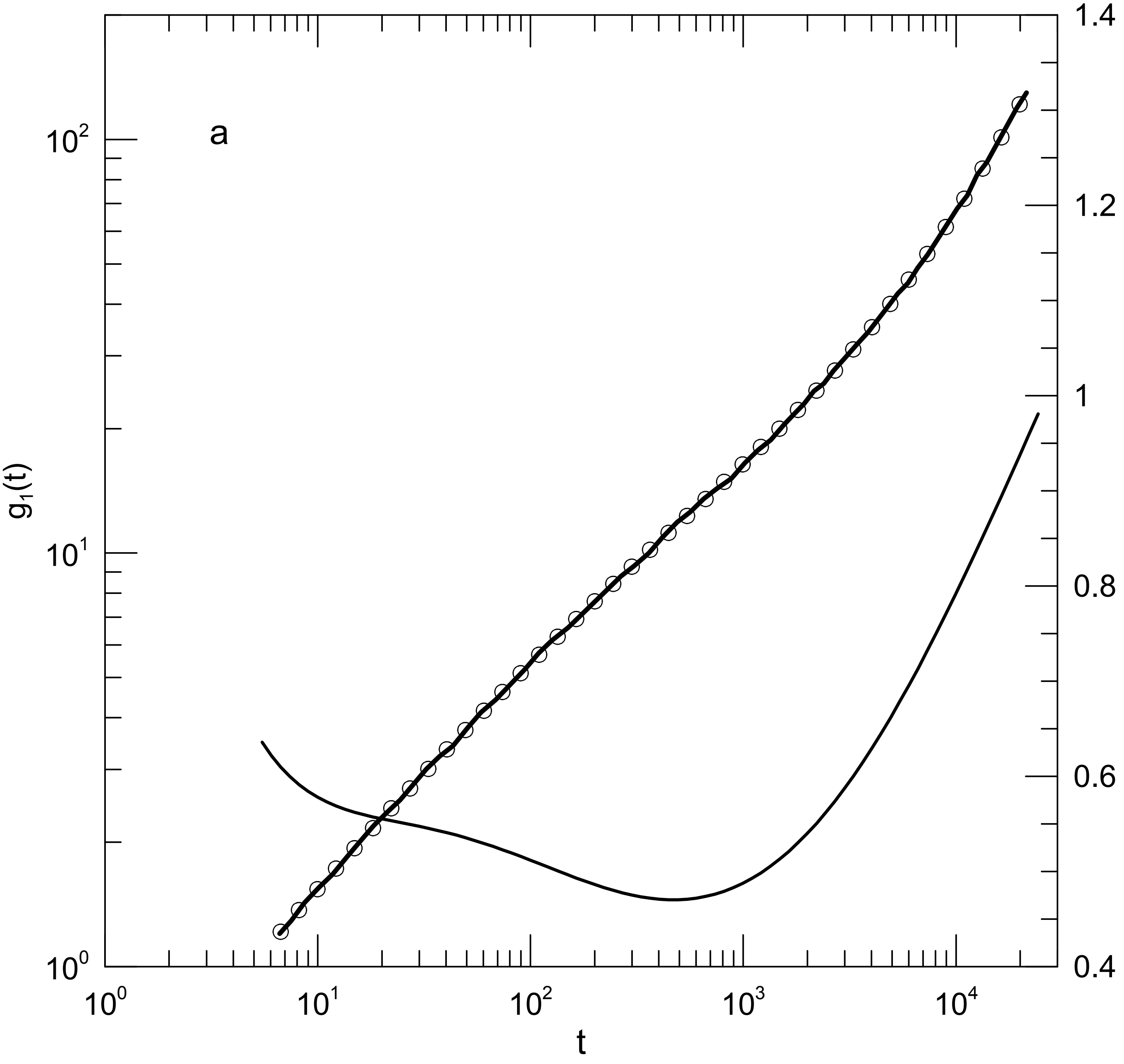

Figure S-14b Mean-square displacements of central beads of 100-bead simulated Grest-Kremer polymer melts, based on Likhtman, et al.[9]. The heavy line represents Likhtman, et al.,'s data, circles show the eighth-order polynomial fit, and the thin solid line is the first derivative K_1 of the polynomial fit.

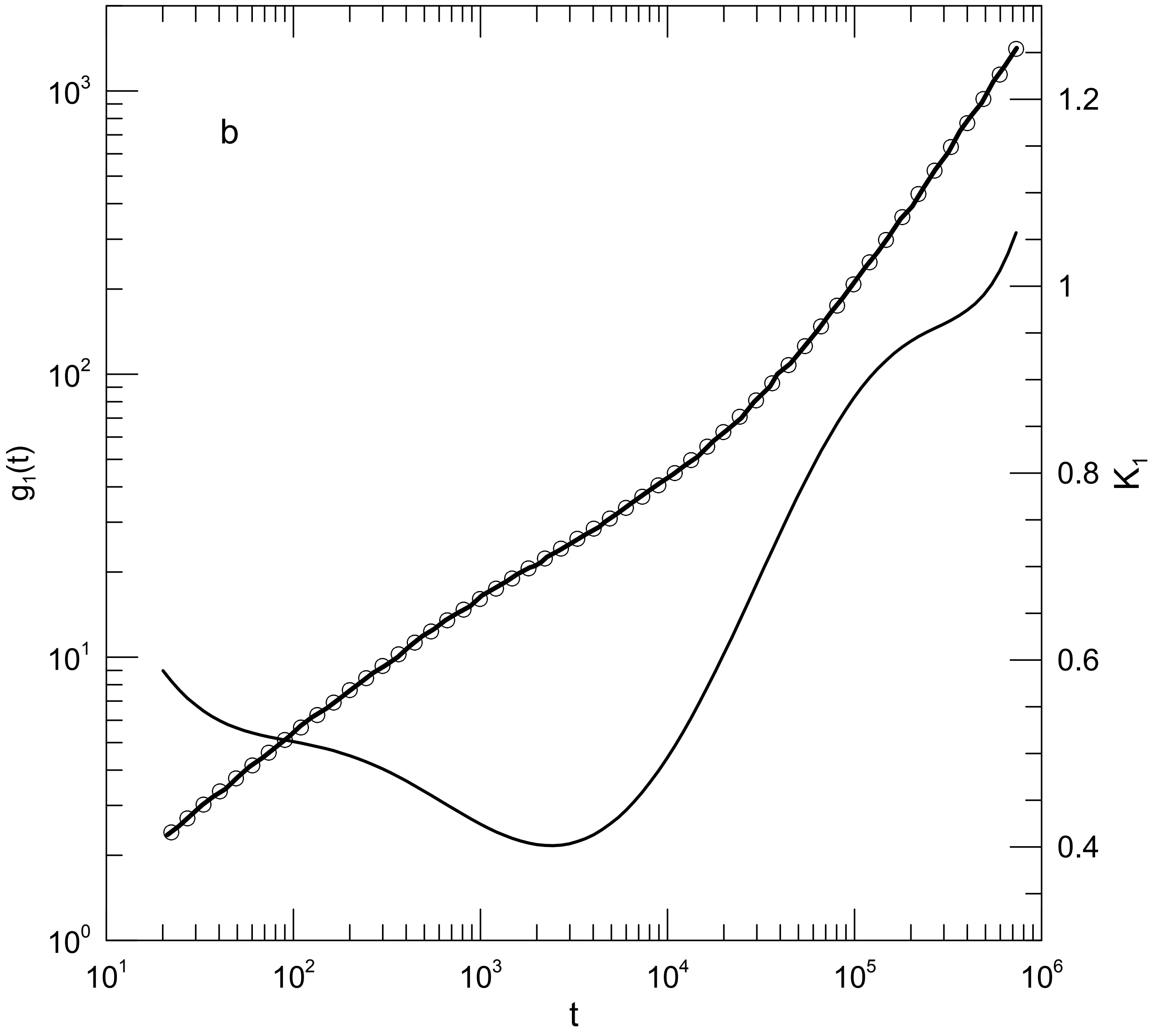

Figure S-14c Mean-square displacements of central beads of 200-bead simulated Grest-Kremer polymer melts, based on Likhtman, et al.[9]. The heavy line represents Likhtman, et al.,'s data, circles show the eighth-order polynomial fit, and the thin solid line is the first derivative K_1 of the polynomial fit.

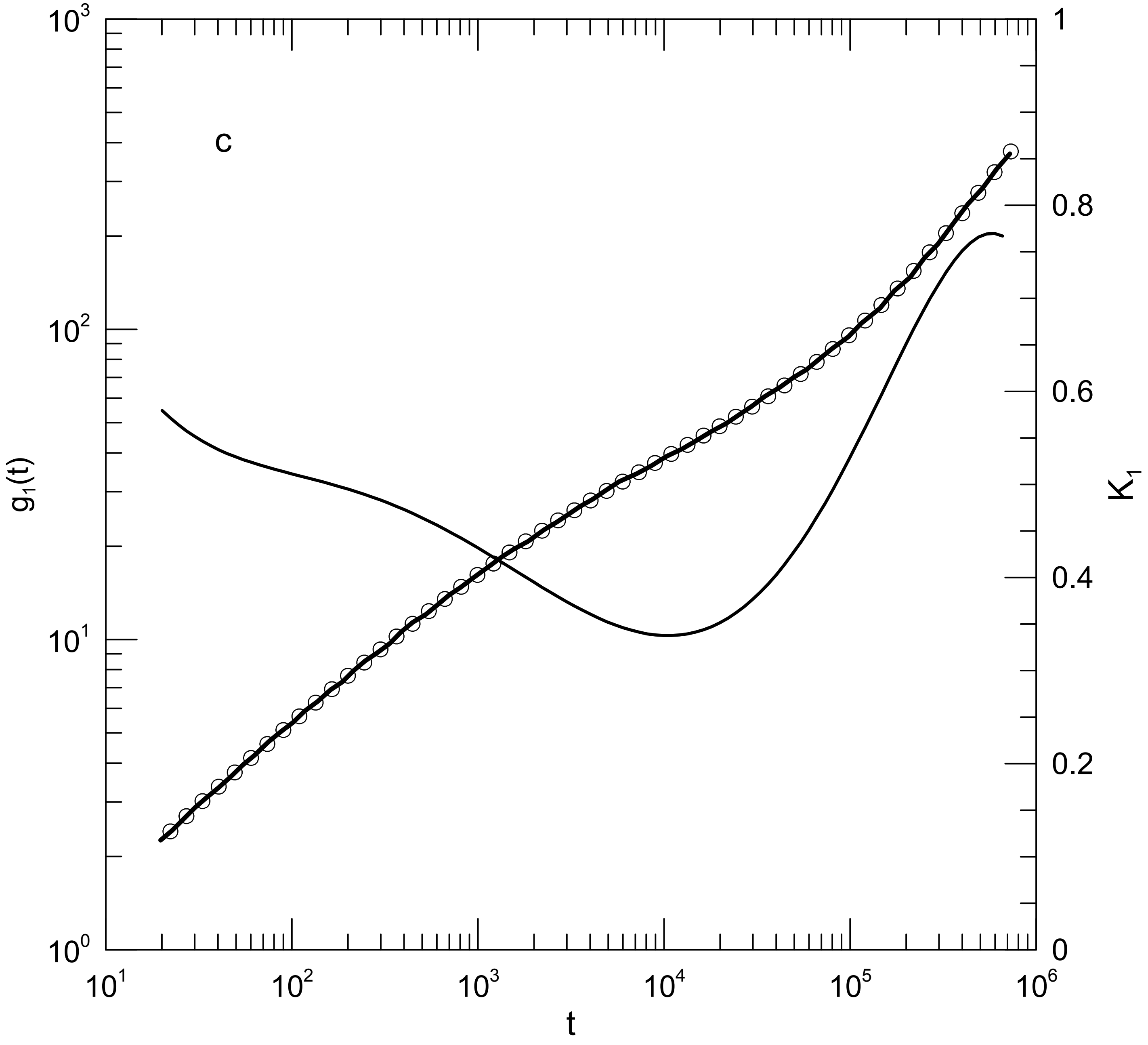

Figure S-14d Mean-square displacements of central beads of 350-bead simulated Grest-Kremer polymer melts, based on Likhtman, et al.[9]. The heavy line represents Likhtman, et al.,'s data, circles show the eighth-order polynomial fit, and the thin solid line is the first derivative K_1 of the polynomial fit.

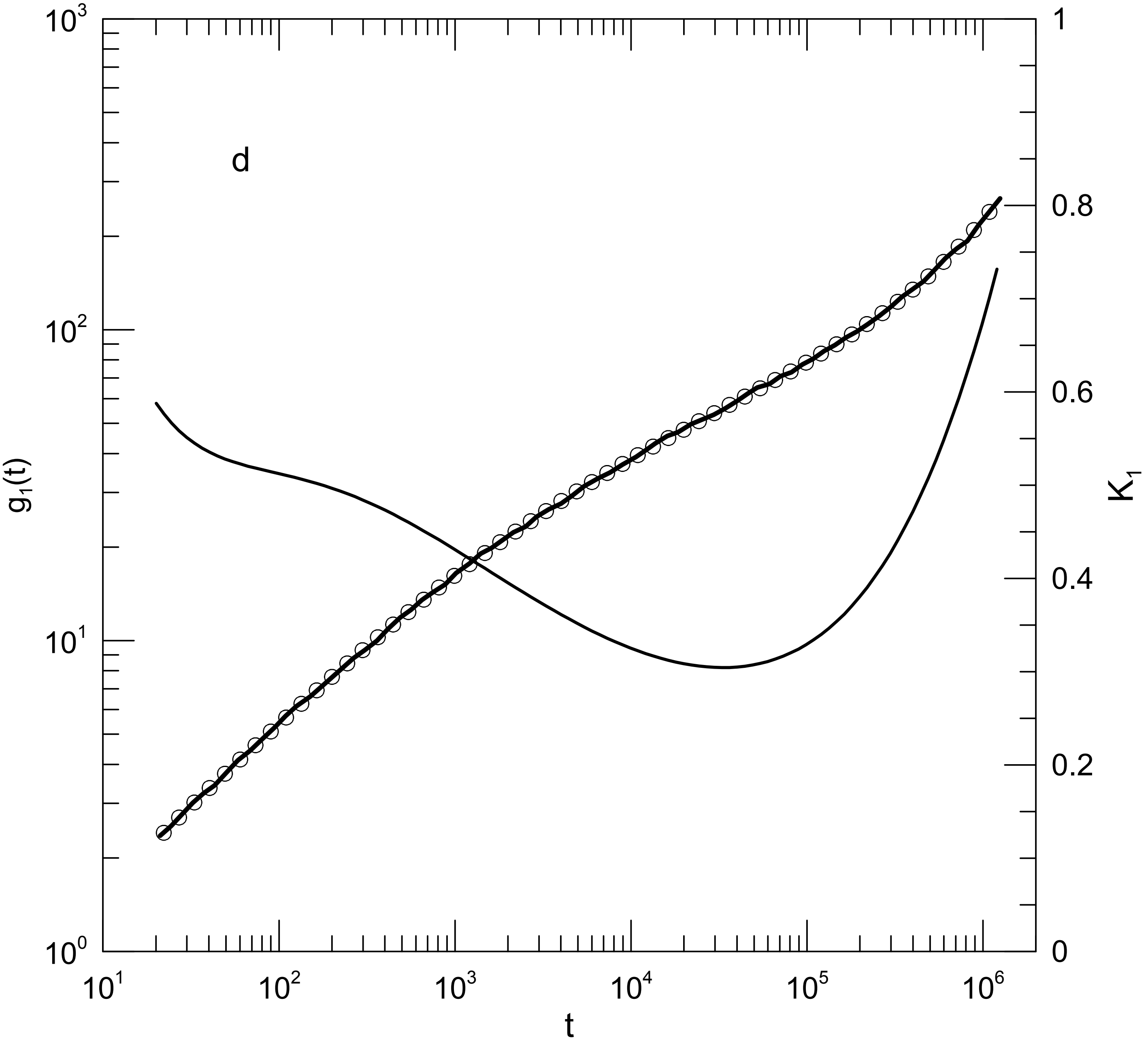

Figure S-15a. Mean-square displacement $g_2(t)$ of a polymer's beads relative to their center of mass, for bead-spring polymers having lengths of 150 beads, based on Zhou and Larson[10]. Heavy line represents Zhou and Larson's data, circles represent an eighth-order polynomial fit, and thin solid line shows the first derivative of the

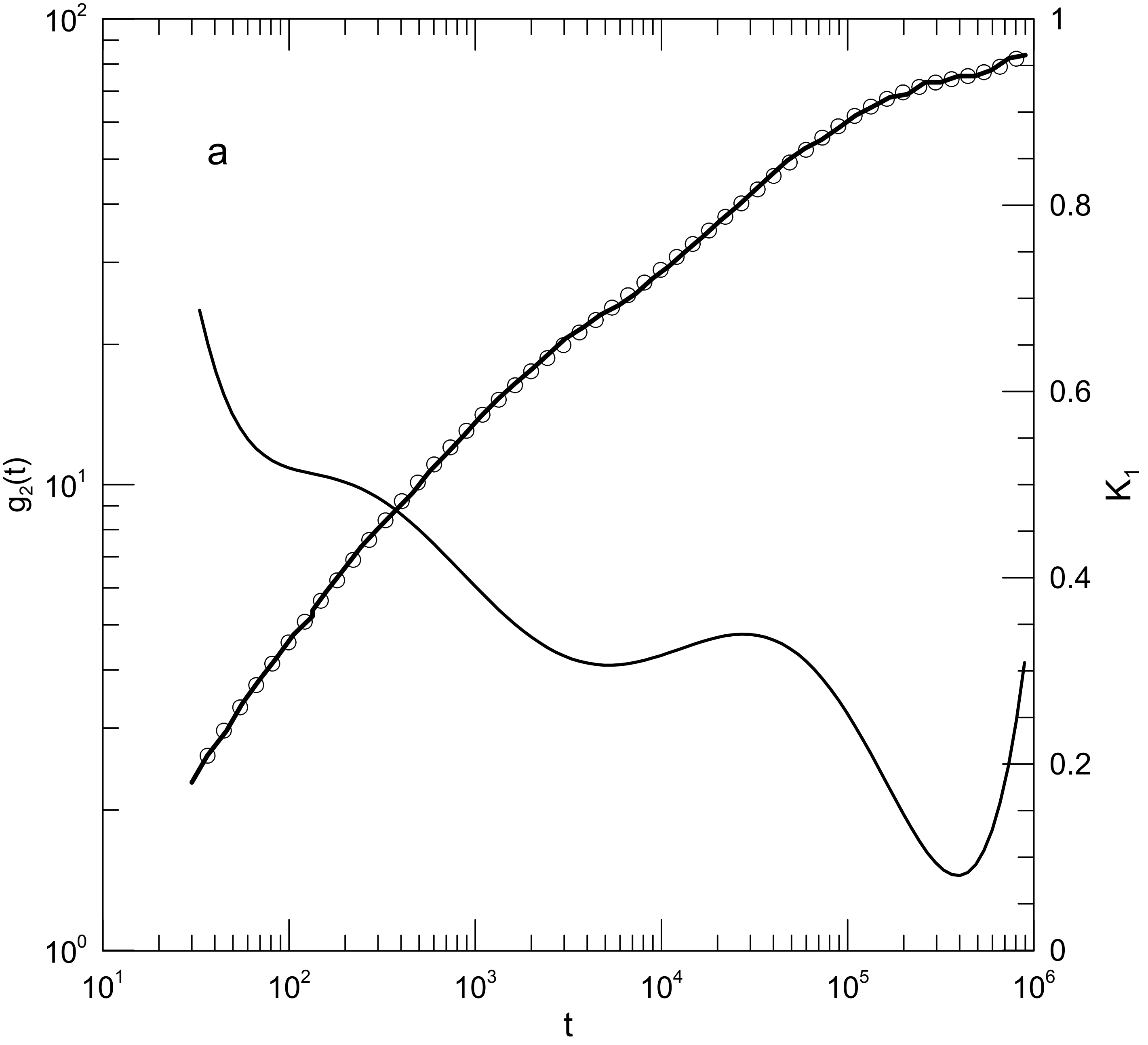

Figure S-15b. Mean-square displacement $g_2(t)$ of a polymer's beads relative to their center of mass, for bead-spring polymers having lengths of 300 beads, based on Zhou and Larson[10]. Heavy line represents Zhou and Larson's data, circles represent an eighth-order polynomial fit, and thin solid line shows the first derivative of the

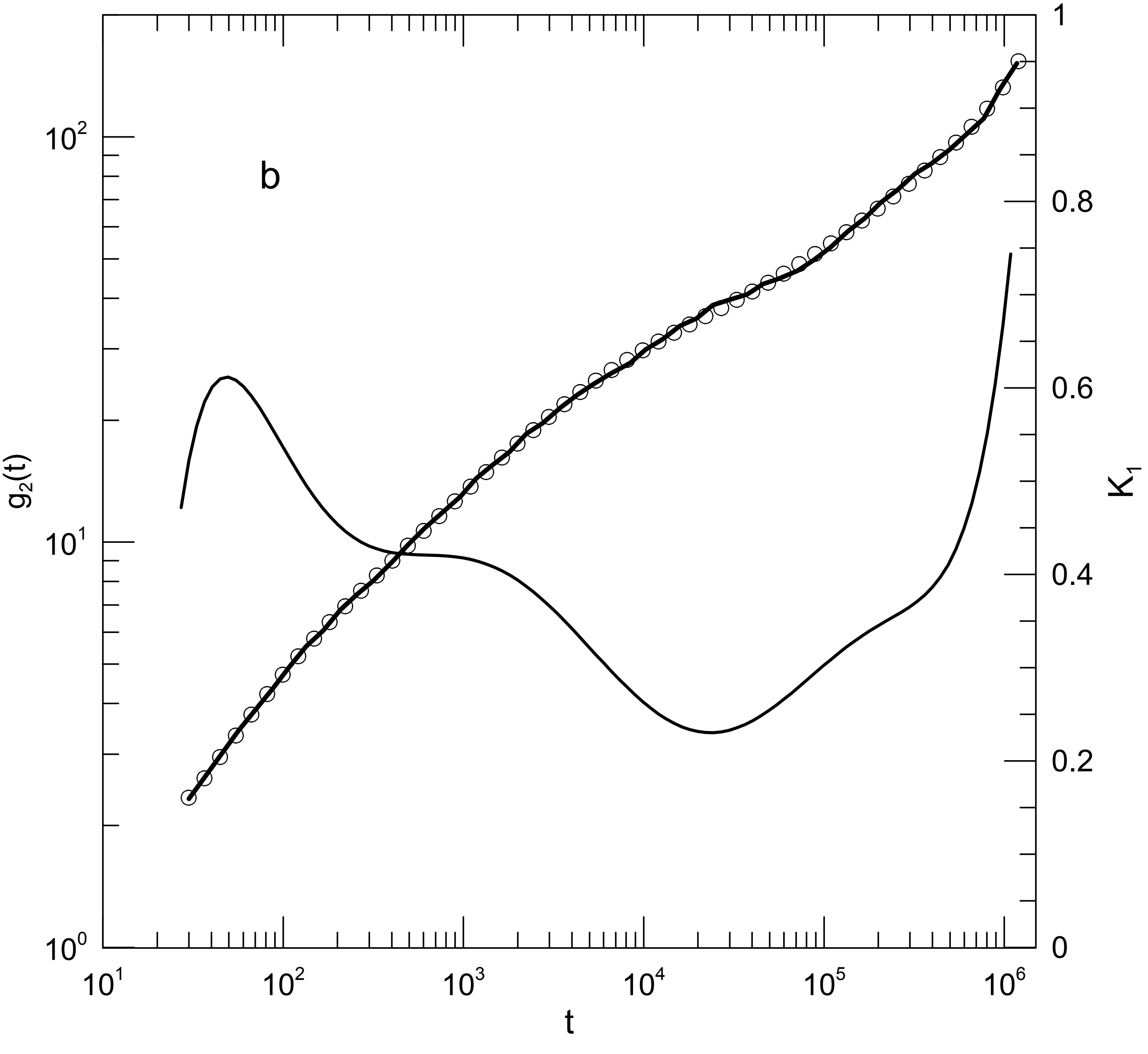

Figure S-16a. Mean-square displacement $g_1(t)$ of individual beads of a pure B melt, data from Moreno, et al.[11] at temperature 1.5. Heavy line represents Moreno, et al.'s data. Circles show an eighth-order polynomial fit. Thin solid line is the first derivative K_1 of the polynomial fit.

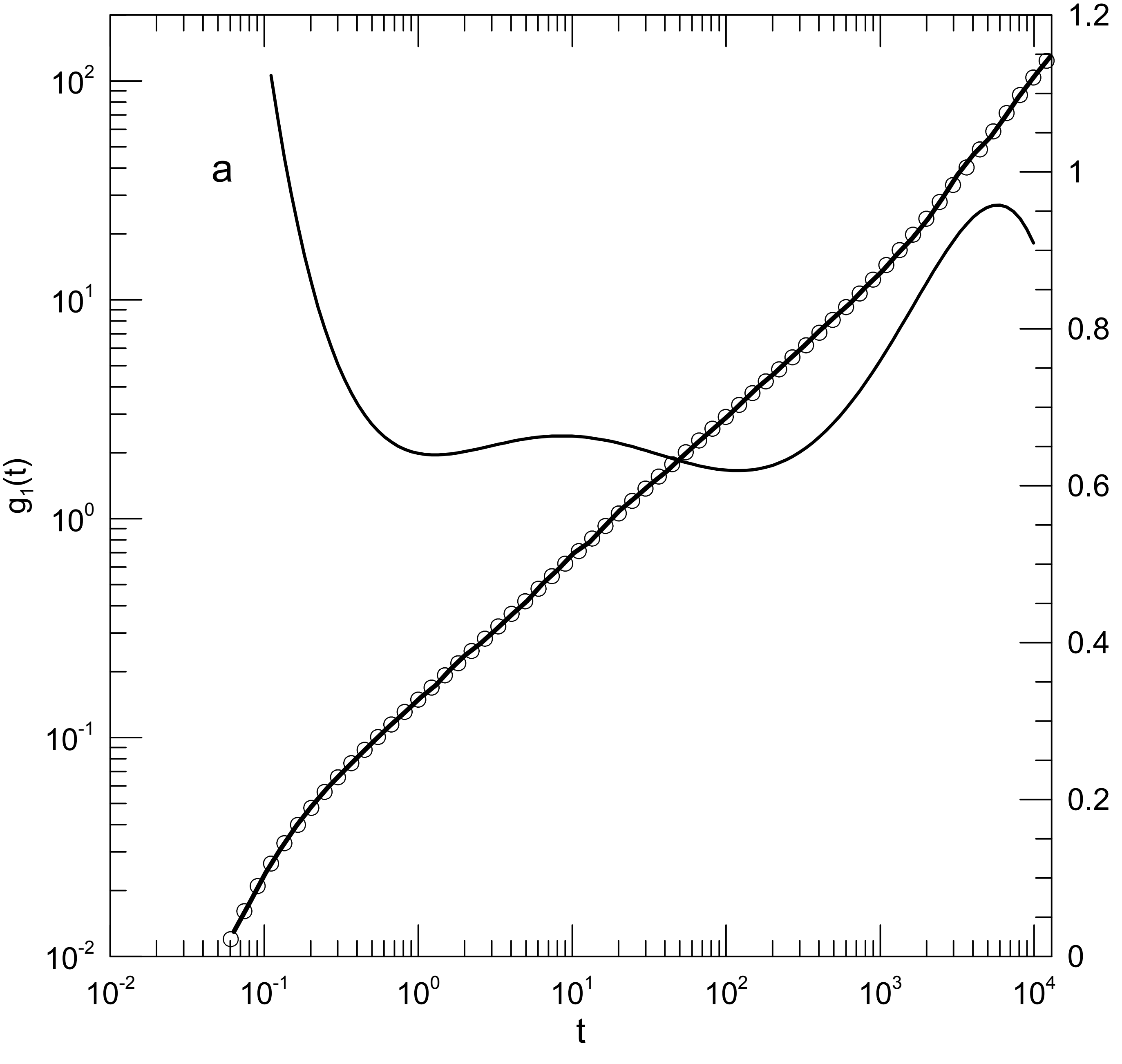

Figure S-16b. Mean-square displacement $g_1(t)$ of individual beads of a pure B melt, data from Moreno, et al.[11] at temperature 1.0. Heavy line represents Moreno, et al.'s data. Circles show an eighth-order polynomial fit. Thin solid line is the first derivative K_1 of the polynomial fit.

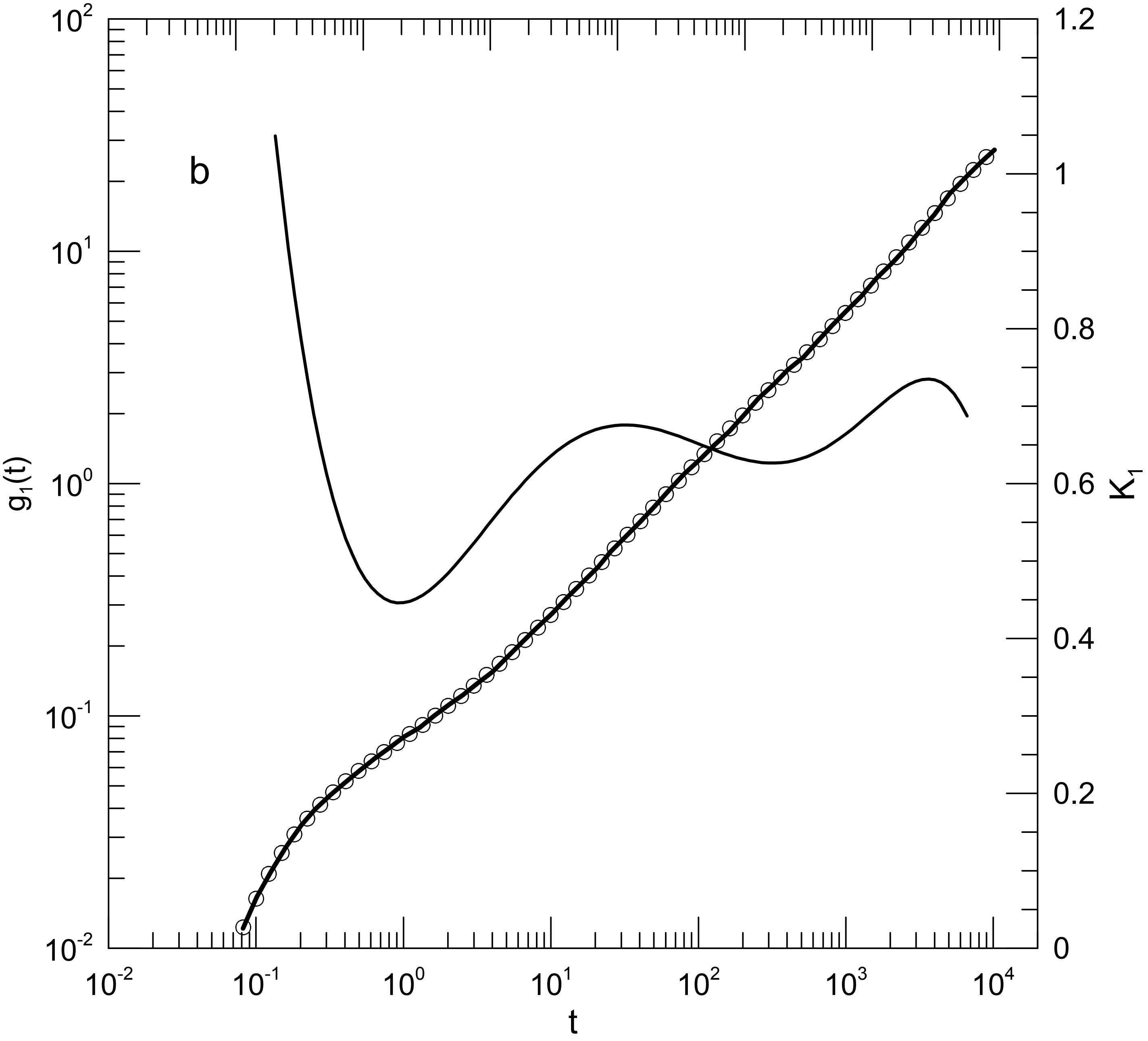

Figure S-16c. Mean-square displacement $g_1(t)$ of individual beads of a pure B melt, data from Moreno, et al.[11] at temperature 0.70. Heavy line represents Moreno, et al.'s data. Circles show an eighth-order polynomial fit. Thin solid line is the first derivative K_1 of the polynomial fit.

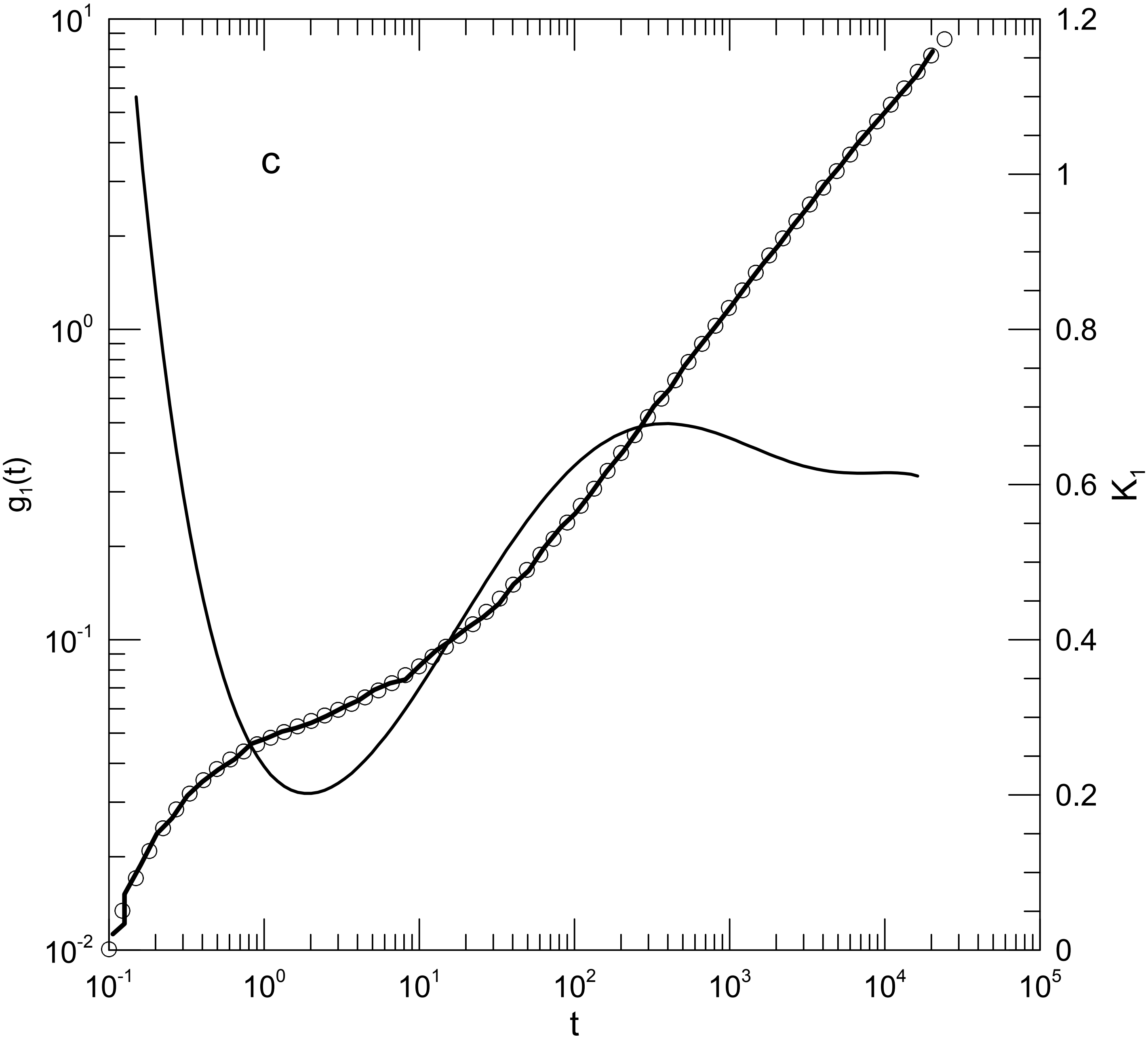

Figure S-16d. Mean-square displacement $g_1(t)$ of individual beads of a pure B melt, data from Moreno, et al.[11] at temperature (a) 0.60. Heavy line represents Moreno, et al.'s data. Circles show an eighth-order polynomial fit. Thin solid line is the first derivative K_1 of the polynomial fit.

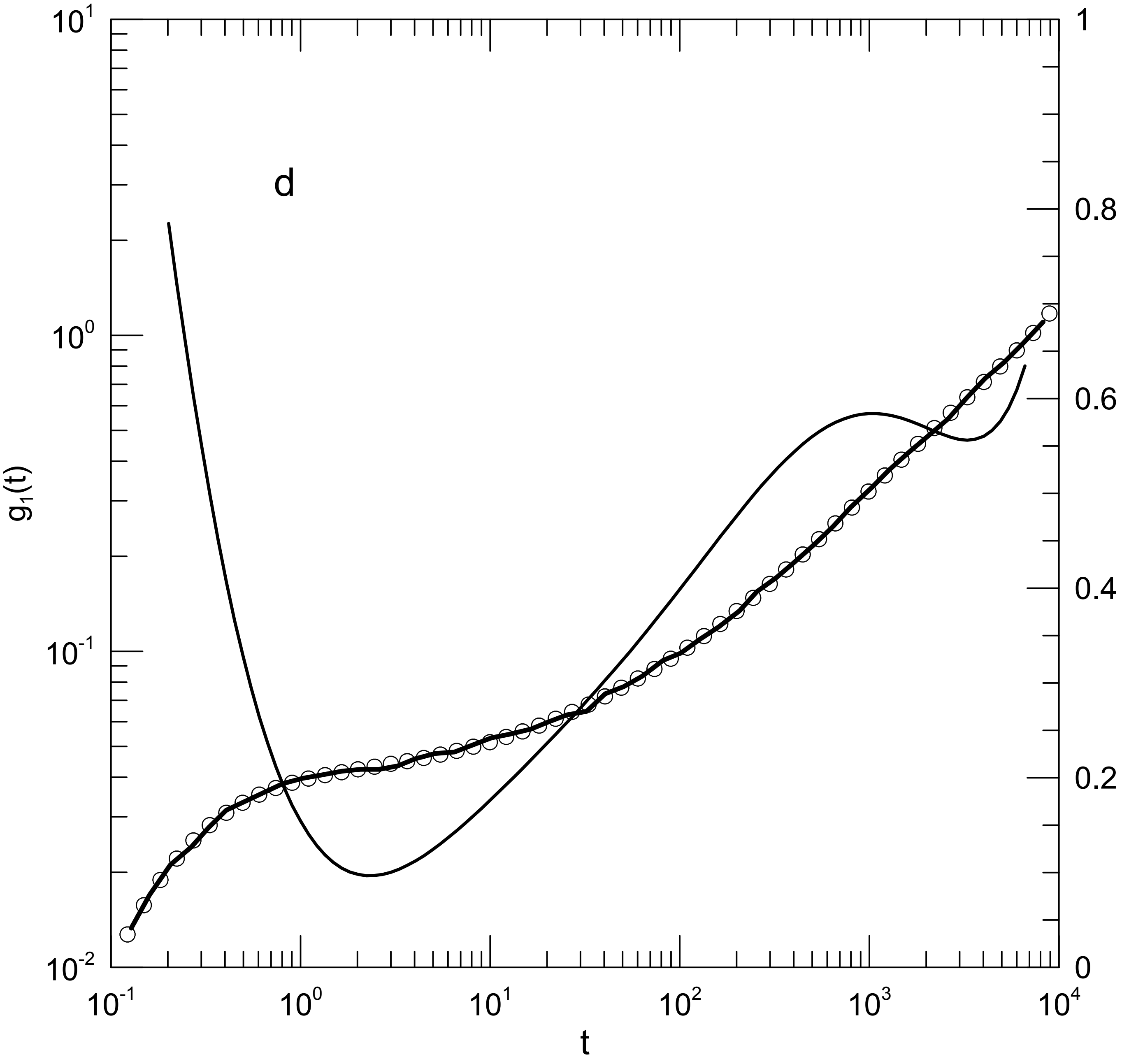

Figure S-16e. Mean-square displacement $g_1(t)$ of individual beads of a pure B melt, data from Moreno, et al.[11] at temperature 0.57. Heavy line represents Moreno, et al.'s data. Circles show an eighth-order polynomial fit. Thin solid line is the first derivative K_1 of the polynomial fit.

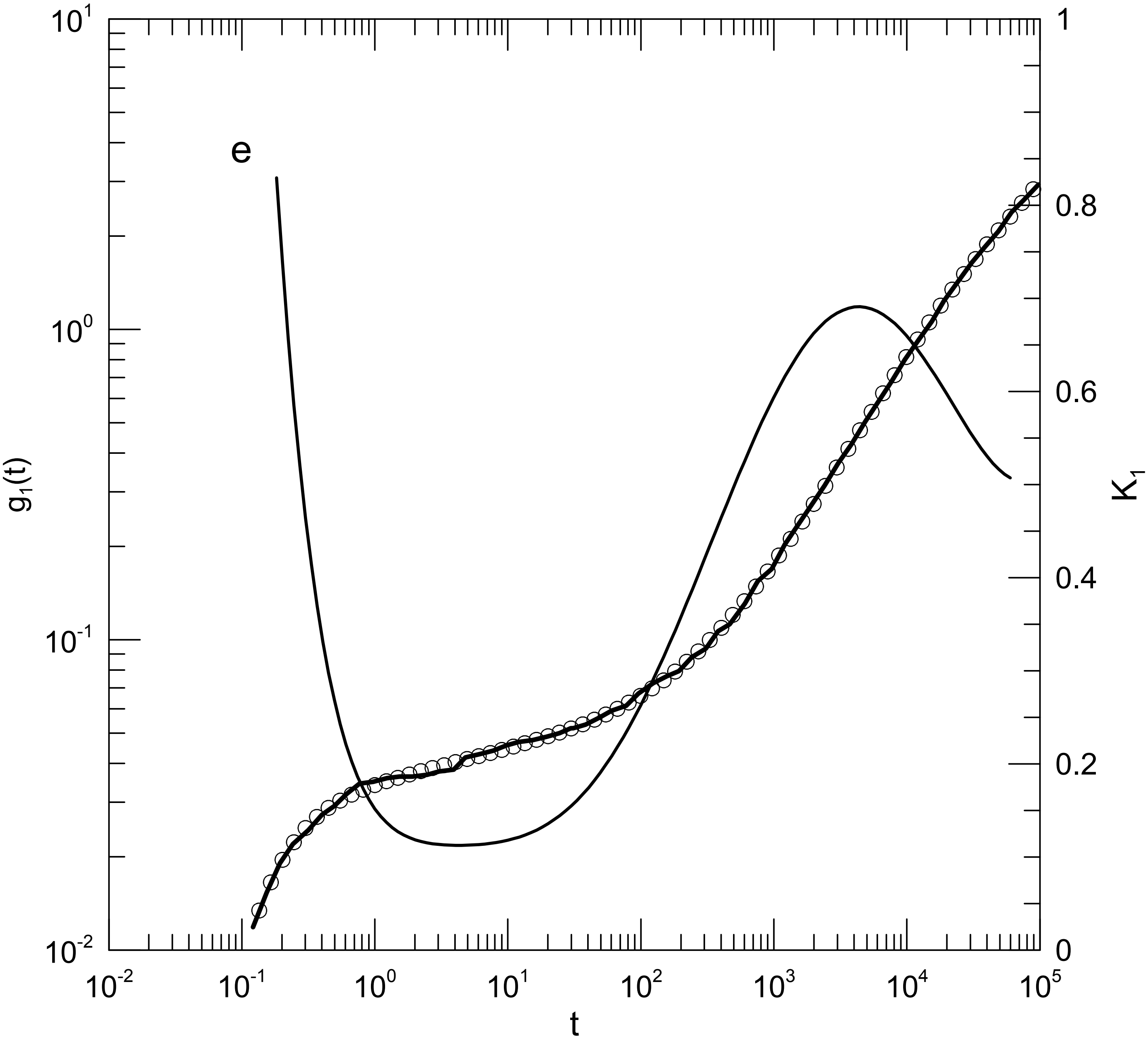

Figure S-16f. Mean-square displacement $g_1(t)$ of individual beads of a pure B melt, data from Moreno, et al.[11] at temperature 0.55. Heavy line represents Moreno, et al.'s data. Circles show an eighth-order polynomial fit. Thin solid line is the first derivative K_1 of the polynomial fit.

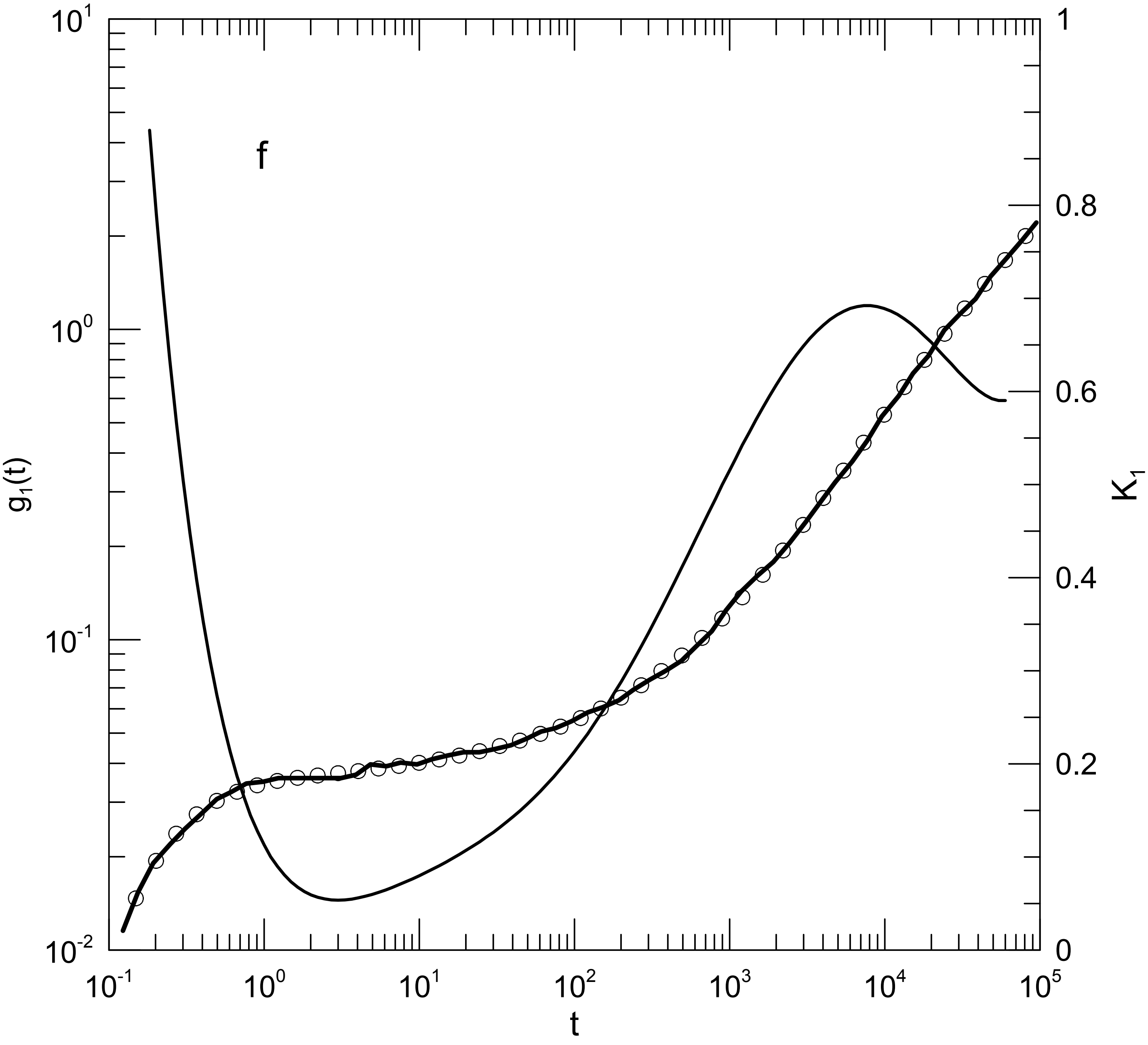

Figure S-17a. Mean-square displacement $g_1(t)$ of the B beads of a melt of an A-B polymer blend with B mole fraction $x_B = 0.3$, based on Moreno, et al.[11] at temperature 1.5. Heavy line represents Moreno, et al.'s data. Circles show the eighth-order polynomial fit. Thin solid line is the first derivative K_1 of the polynomial fit.

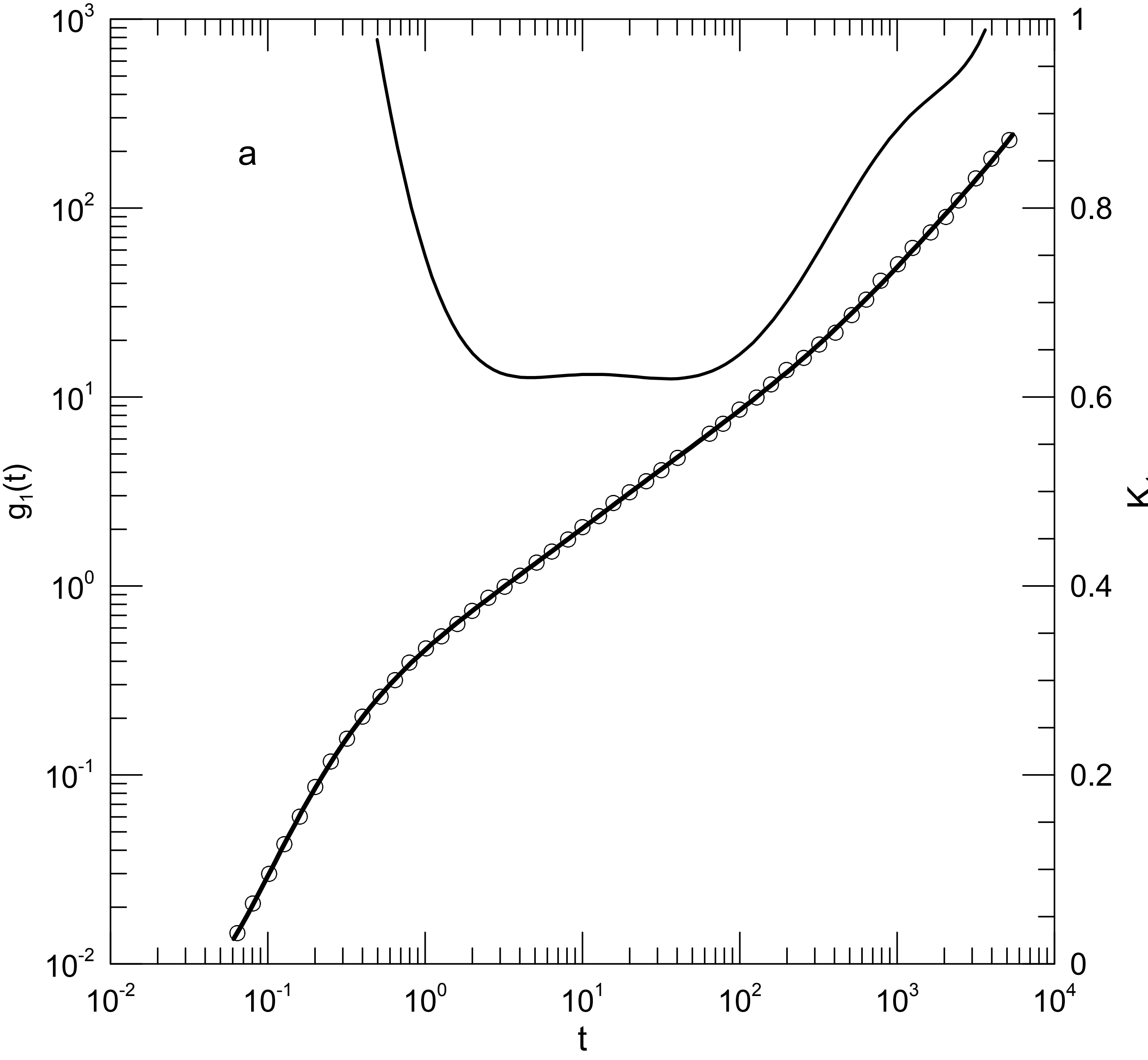

Figure S-17b. Mean-square displacement $g_1(t)$ of the B beads of a melt of an A-B polymer blend with B mole fraction $x_B = 0.3$, based on Moreno, et al.[11] at temperature 1.0. Heavy line represents Moreno, et al.'s data. Circles show the eighth-order polynomial fit. Thin solid line is the first derivative K_1 of the polynomial fit.

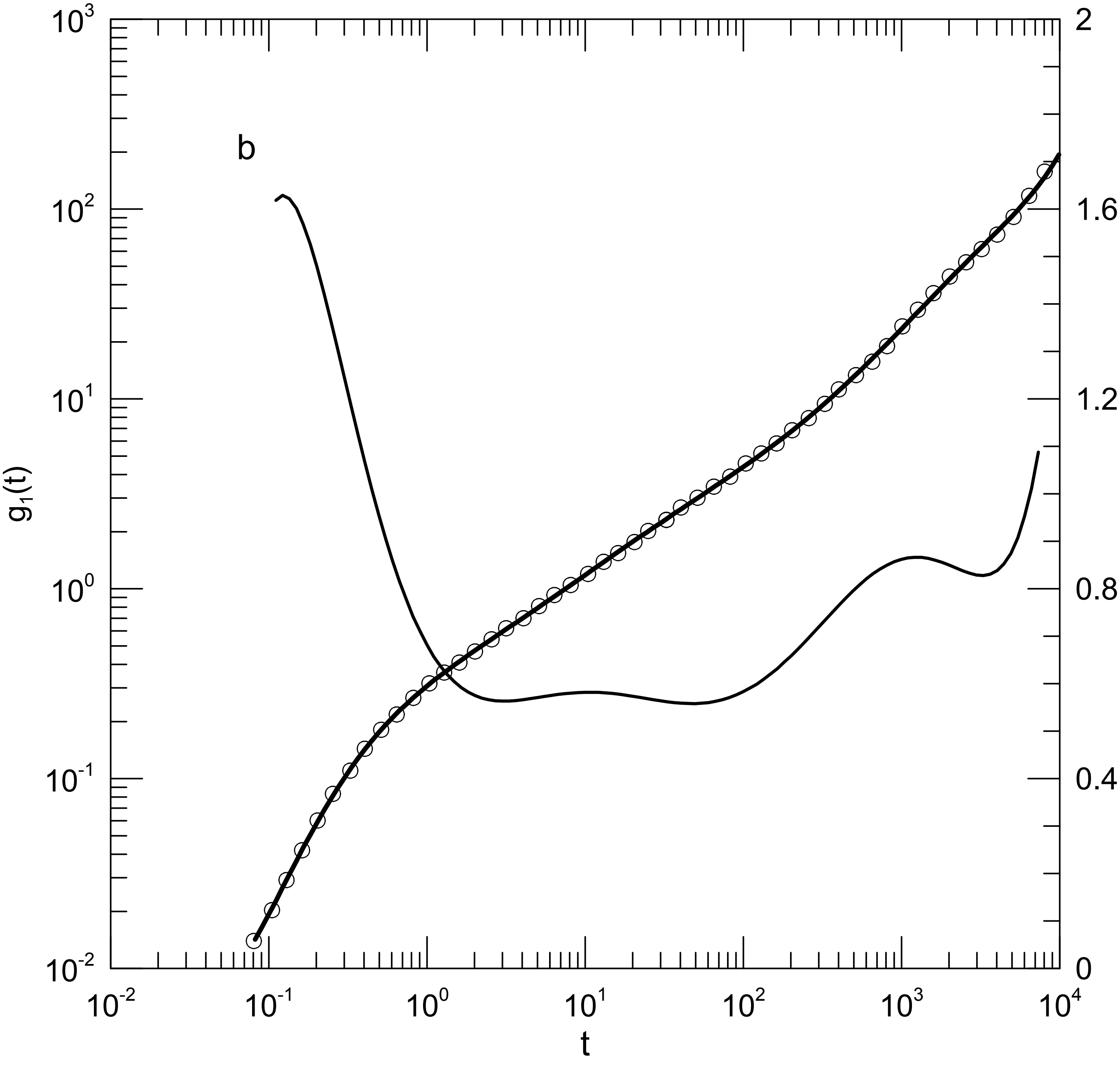

Figure S-17c. Mean-square displacement $g_1(t)$ of the B beads of a melt of an A-B polymer blend with B mole fraction $x_B = 0.3$, based on Moreno, et al.[11] at temperature 0.75. Heavy line represents Moreno, et al.'s data. Circles show the eighth-order polynomial fit. Thin solid line is the first derivative K_1 of the polynomial fit.

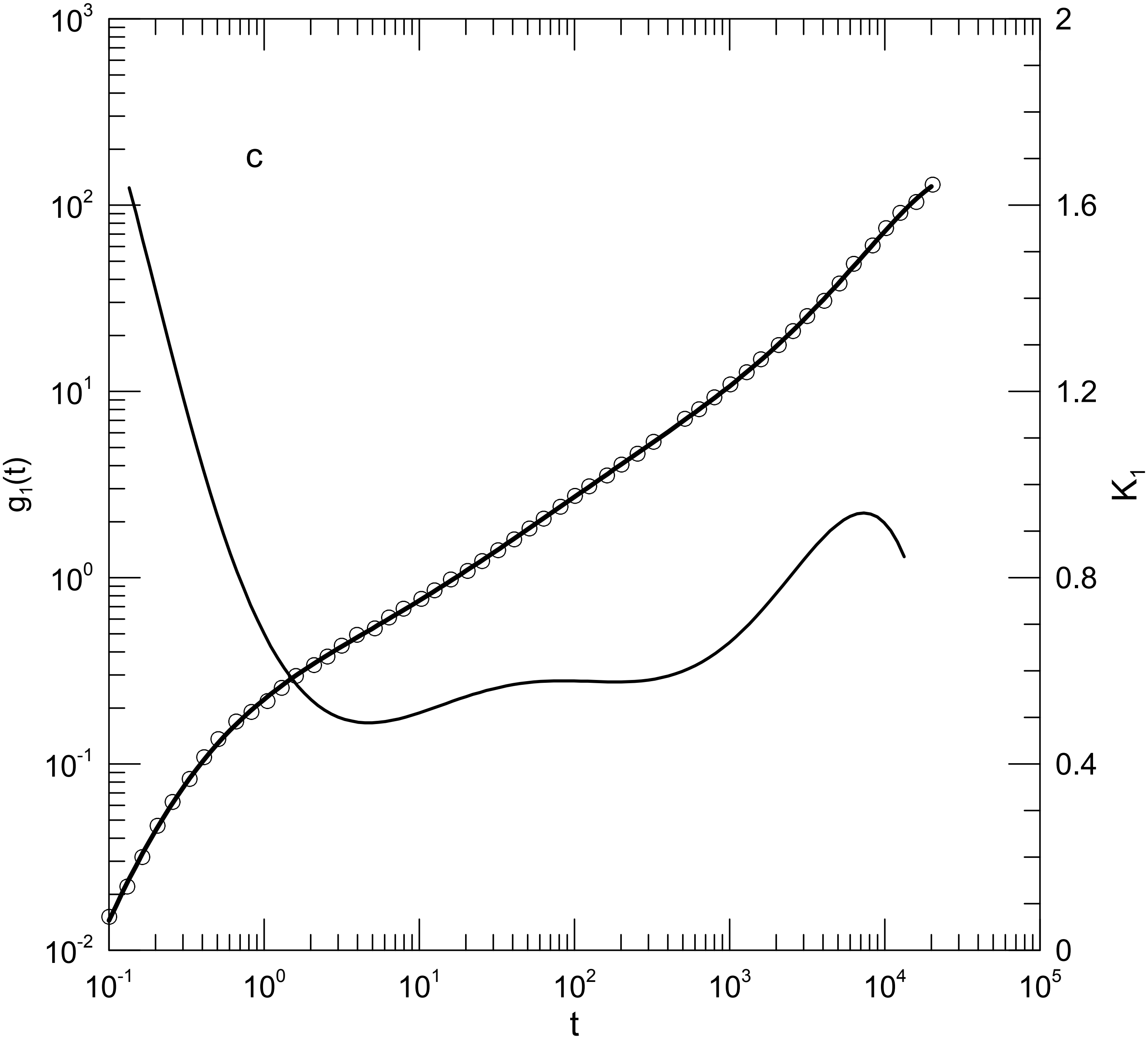

Figure S-17d. Mean-square displacement $g_1(t)$ of the B beads of a melt of an A-B polymer blend with B mole fraction $x_B = 0.3$, based on Moreno, et al.[11] at temperature 0.60. Heavy line represents Moreno, et al.'s data. Circles show the eighth-order polynomial fit. Thin solid line is the first derivative K_1 of the polynomial fit.

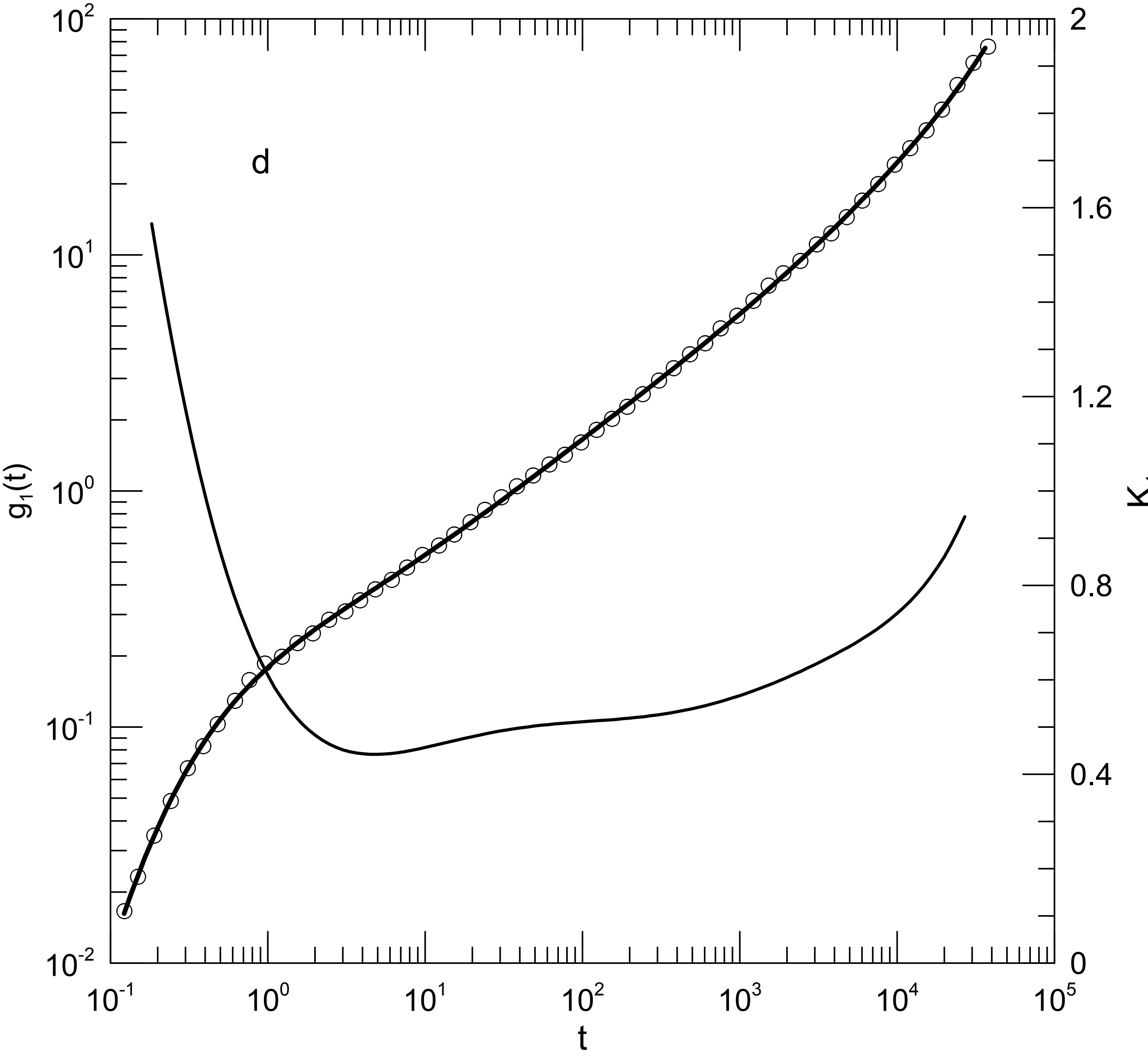

Figure S-17e. Mean-square displacement $g_1(t)$ of the B beads of a melt of an A-B polymer blend with B mole fraction $x_B = 0.3$, based on Moreno, et al.[11] at temperature 0.50. Heavy line represents Moreno, et al.'s data. Circles show the eighth-order polynomial fit. Thin solid line is the first derivative K_1 of the polynomial fit.

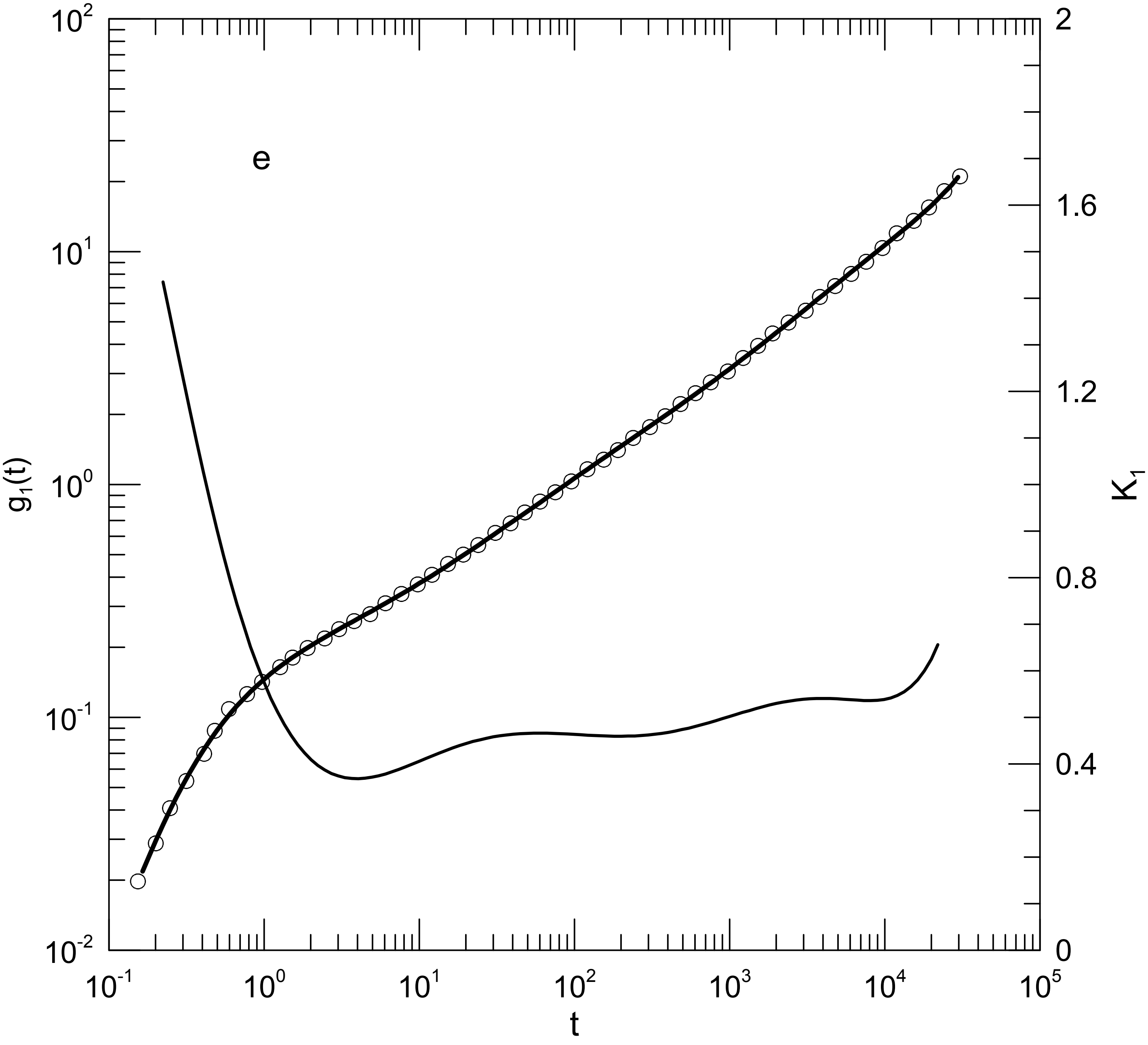

Figure S-17f. Mean-square displacement $g_1(t)$ of the B beads of a melt of an A-B polymer blend with B mole fraction $x_B = 0.3$, based on Moreno, et al.[11] at temperature 0.45. Heavy line represents Moreno, et al.'s data. Circles show the eighth-order polynomial fit. Thin solid line is the first derivative K_1 of the polynomial fit.

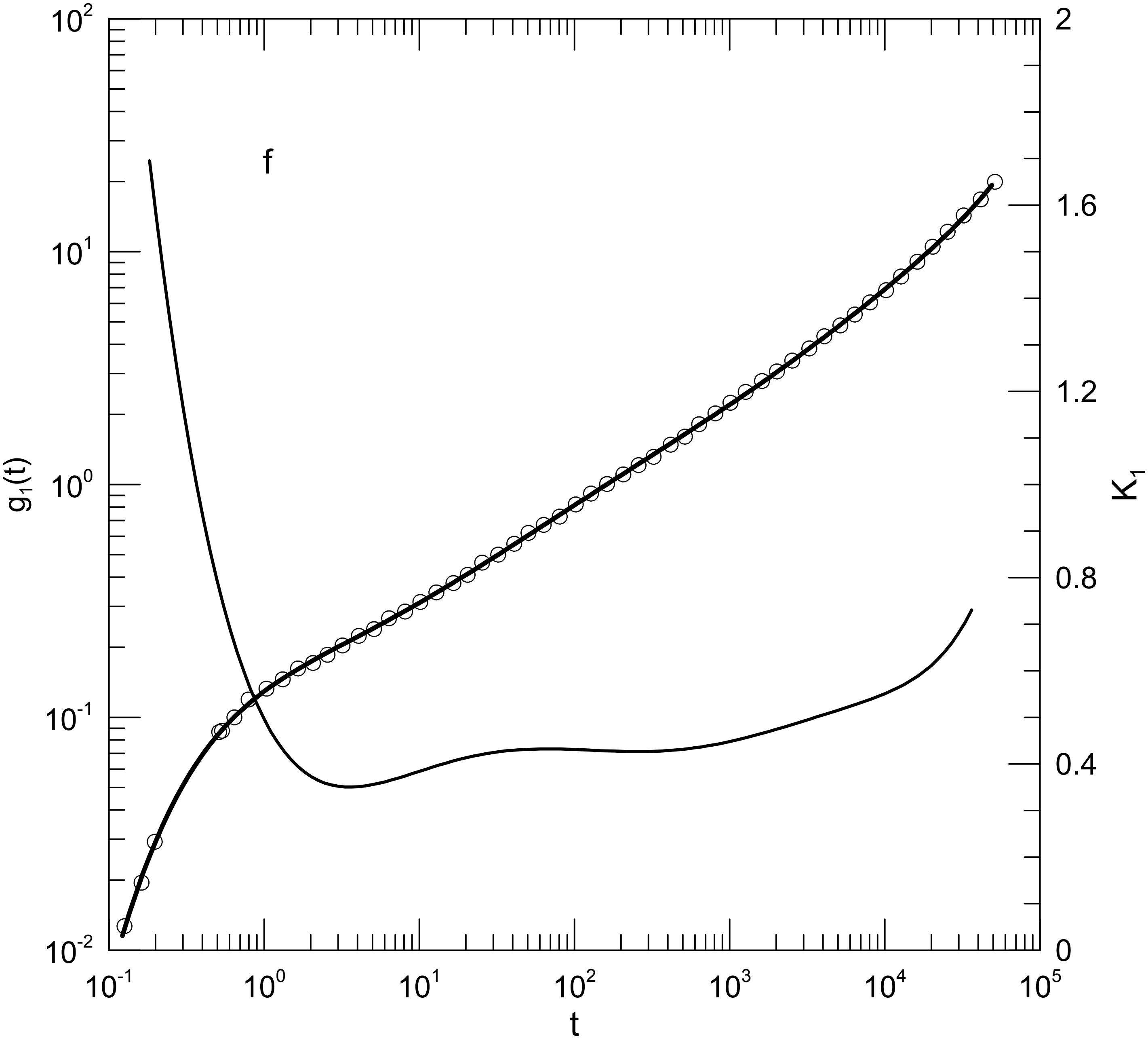

Figure S-17g. Mean-square displacement $g_1(t)$ of the B beads of a melt of an A-B polymer blend with B mole fraction $x_B = 0.3$, based on Moreno, et al.[11] at temperature 0.40. Heavy line represents Moreno, et al.'s data. Circles show the eighth-order polynomial fit. Thin solid line is the first derivative K_1 of the polynomial fit.

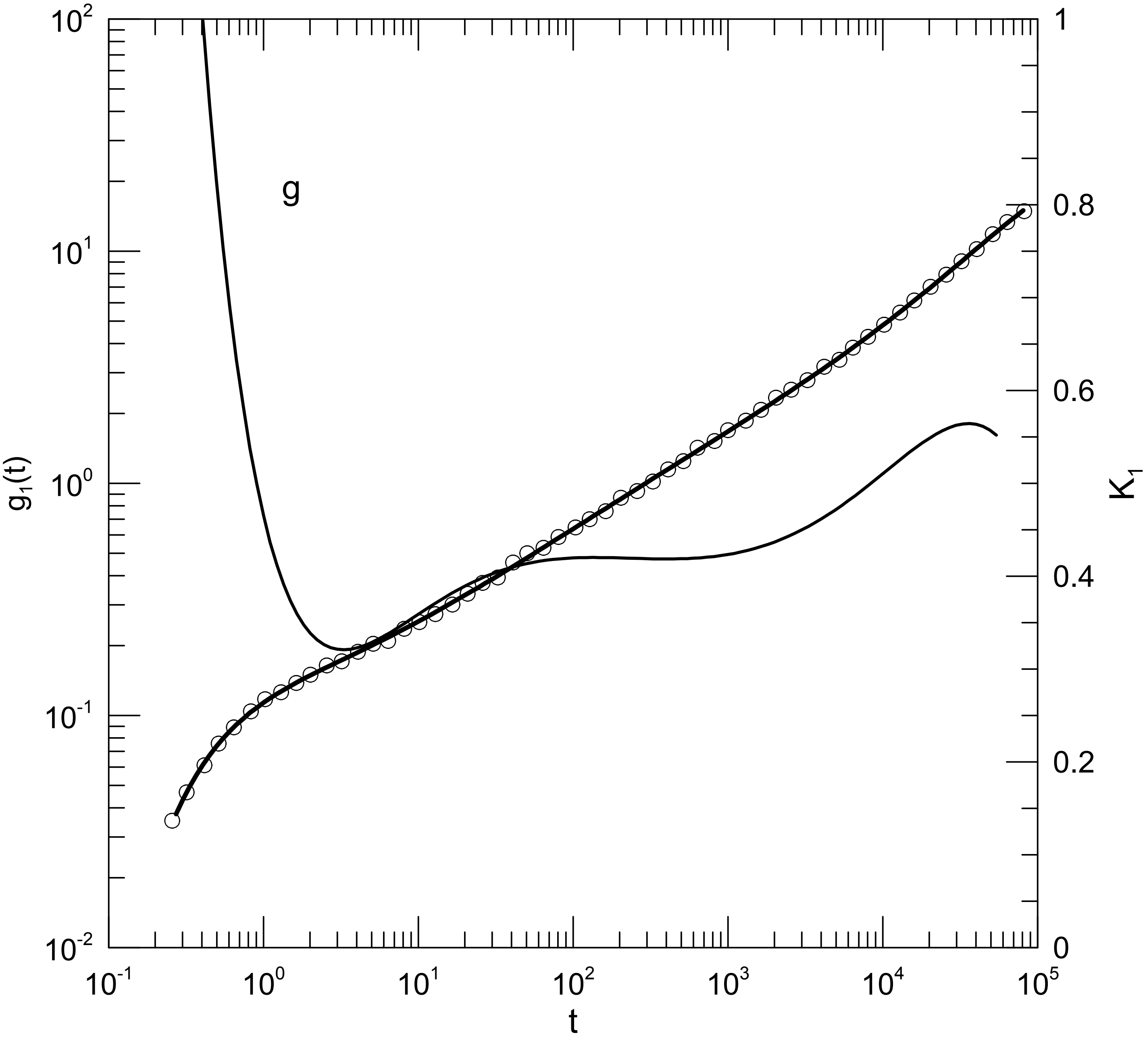

Figure S-18a. Mean-square displacements of individual atoms for a simulated T = 450K melt of n-$C_{120}H_{242}$. Heavy line represents Padding and Briel's data[12]. Circles show the eighth-order polynomial fit. Thin solid line is the first derivative K_1 of the polynomial fit.

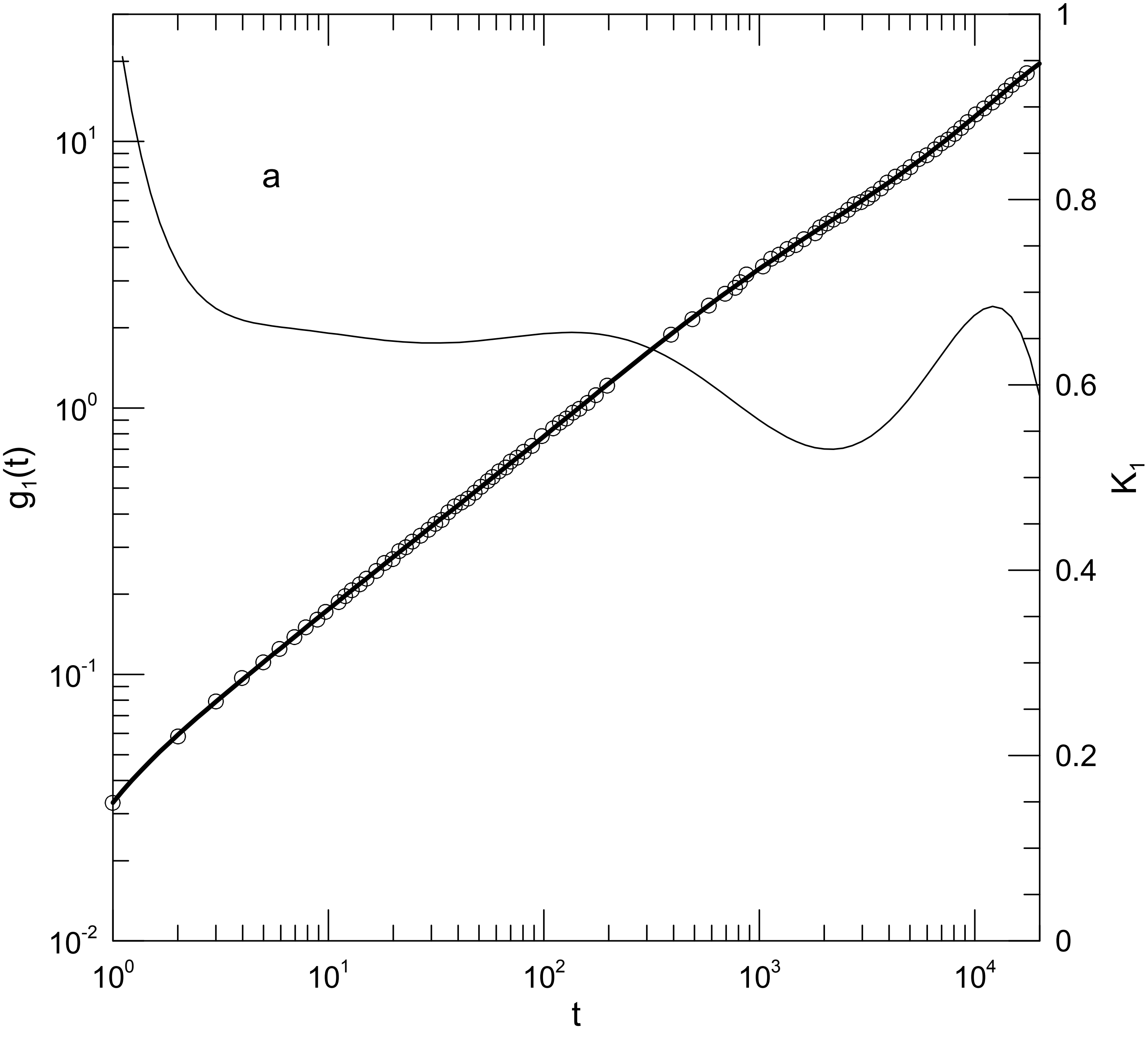

Figure S-18b. Mean-square displacements of (a) individual atoms, (b) chain centers of mass, and (c) individual blobs $g_{\rm bl}(t)$ for a simulated T = 450K melt of n-$C_{120}H_{242}$. Heavy line represents Padding and Briel's data.[12] Circles show the eighth-order polynomial fit. Thin solid line is the first derivative K_1 of the polynomial fit.

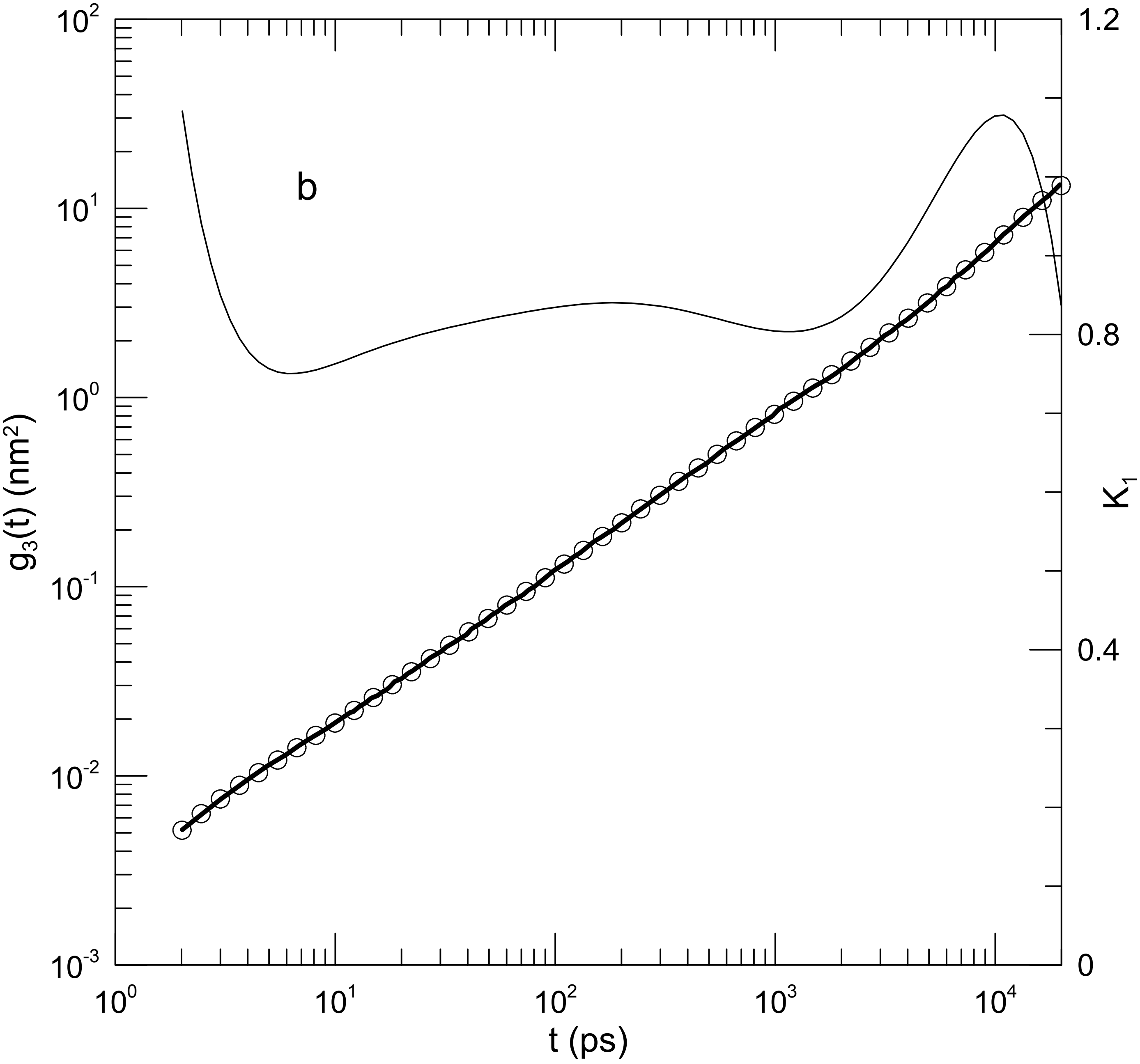

Figure S-18c. Mean-square displacements of (a) individual atoms, (b) chain centers of mass, and (c) individual blobs $g_{\text{bl}}(t)$ for a simulated $T = 450\text{K}$ melt of n-$C_{120}H_{242}$. Heavy line represents Padding and Briel's data.[12] Circles show the eighth-order polynomial fit. Thin solid line is the first derivative K_1 of the polynomial fit.

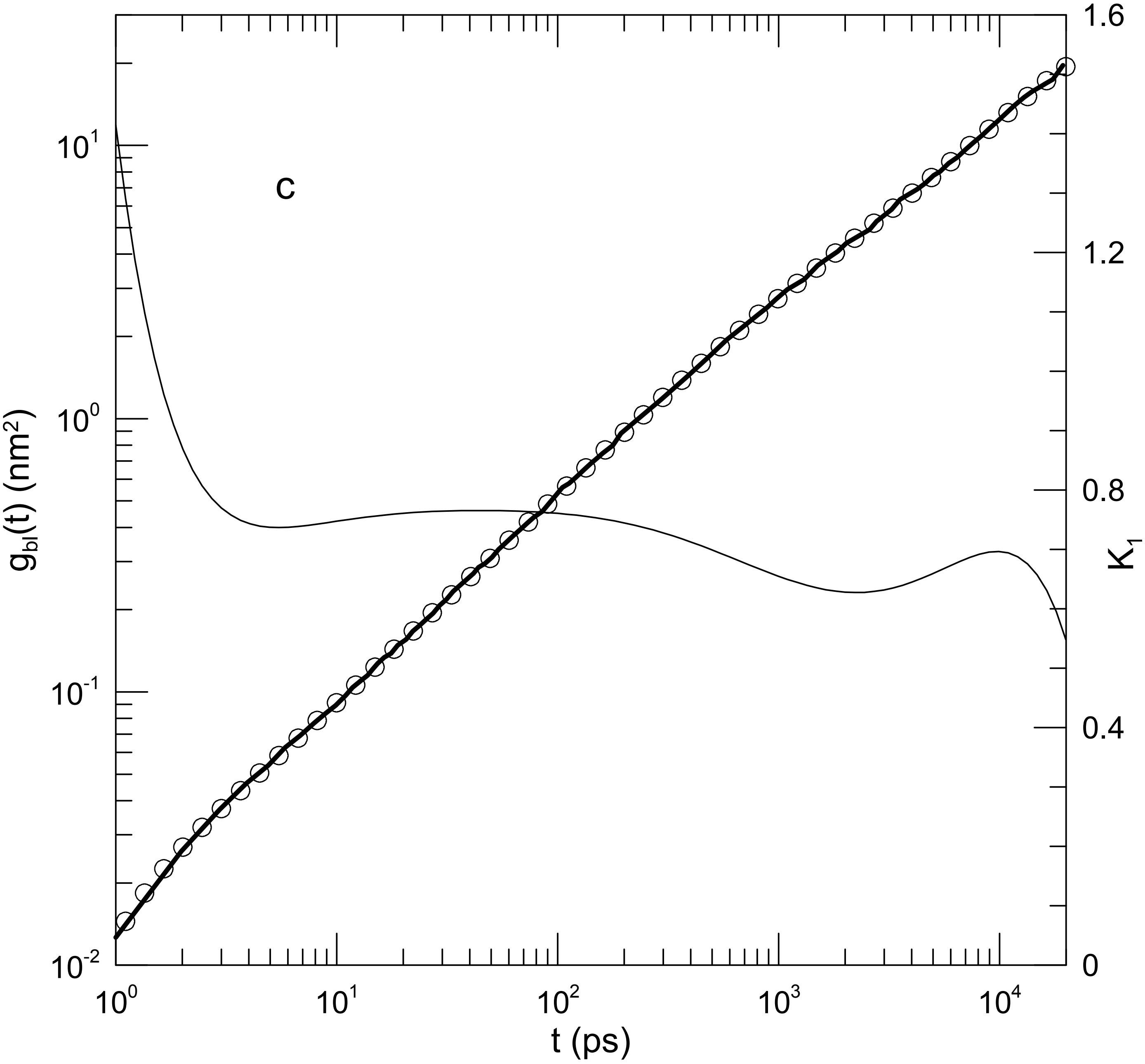

Figure S-19a. Mean-square displacements $g_{\mathrm{bl}}(t)$ of blobs in polyethylene melts containing 4 blobs (and, correspondingly, 80 monomers). Heavy line represents Padding and Briel's data[13]. Circles show the eighth-order polynomial fit. Thin solid line is the first derivative K_1 of the polynomial fit.

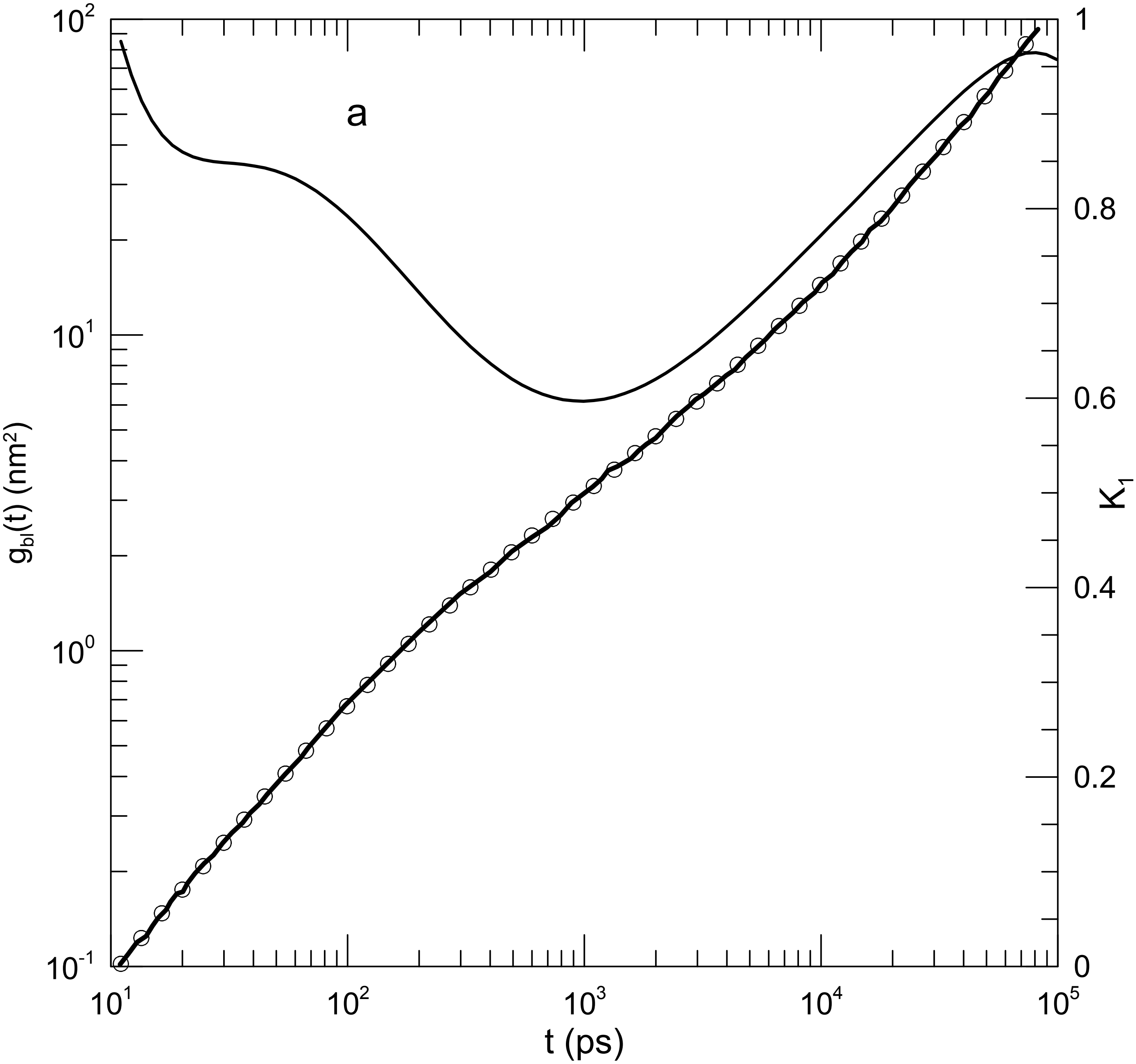

Figure S-19b. Mean-square displacements $g_{\rm bl}(t)$ of blobs in polyethylene melts containing 6 (and, correspondingly, 120 monomers). Heavy line represents Padding and Briel's data[13]. Circles show the eighth-order polynomial fit. Thin solid line is the first derivative K_1 of the polynomial fit.

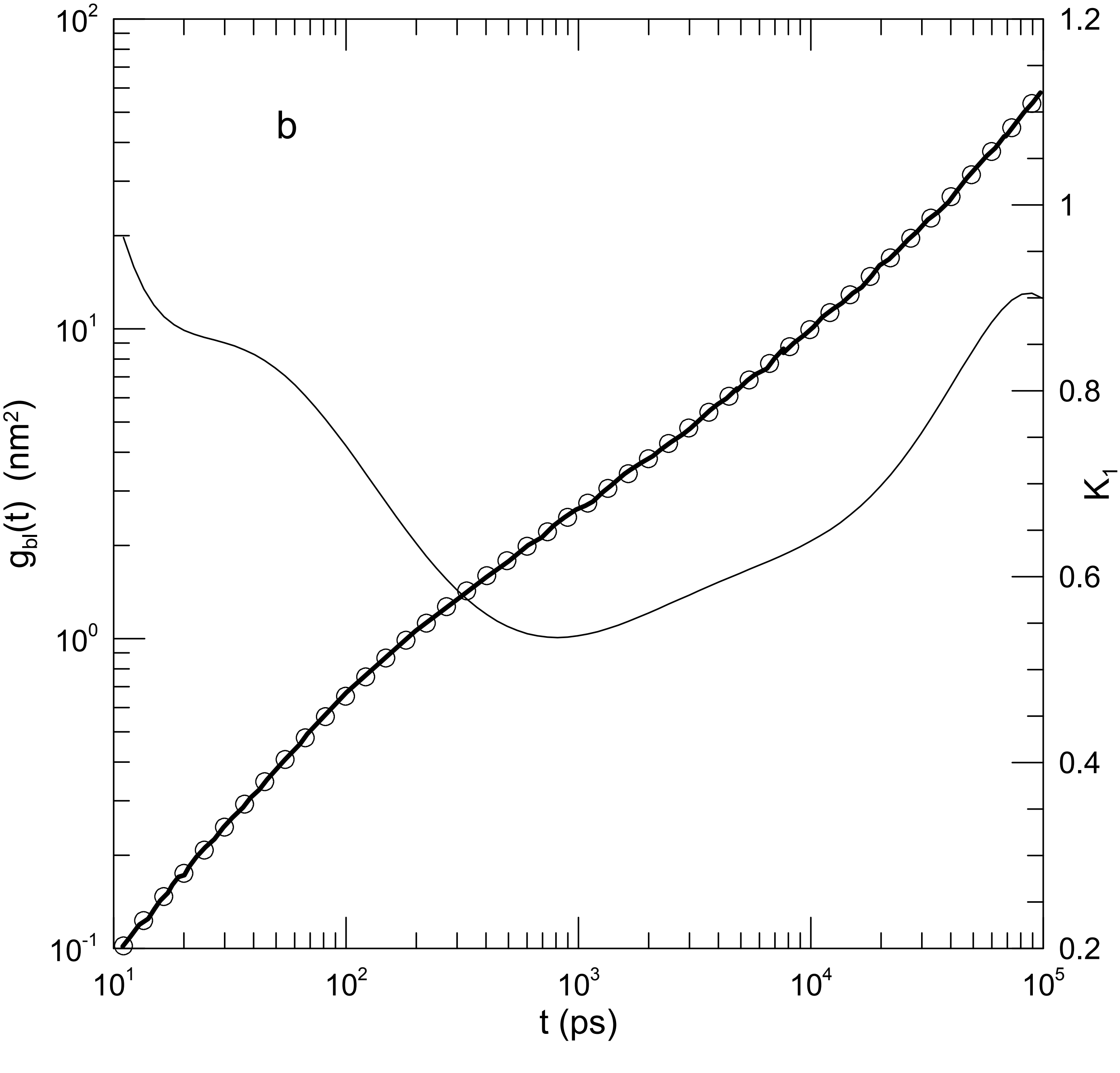

Figure S-19c. Mean-square displacements $g_{\mathrm{bl}}(t)$ of blobs in polyethylene melts containing 50 blobs (and, correspondingly, 1000 monomers). Heavy line represents Padding and Briel's data[13]. Circles show the eighth-order polynomial fit. Thin solid line is the first derivative K_1 of the polynomial fit.

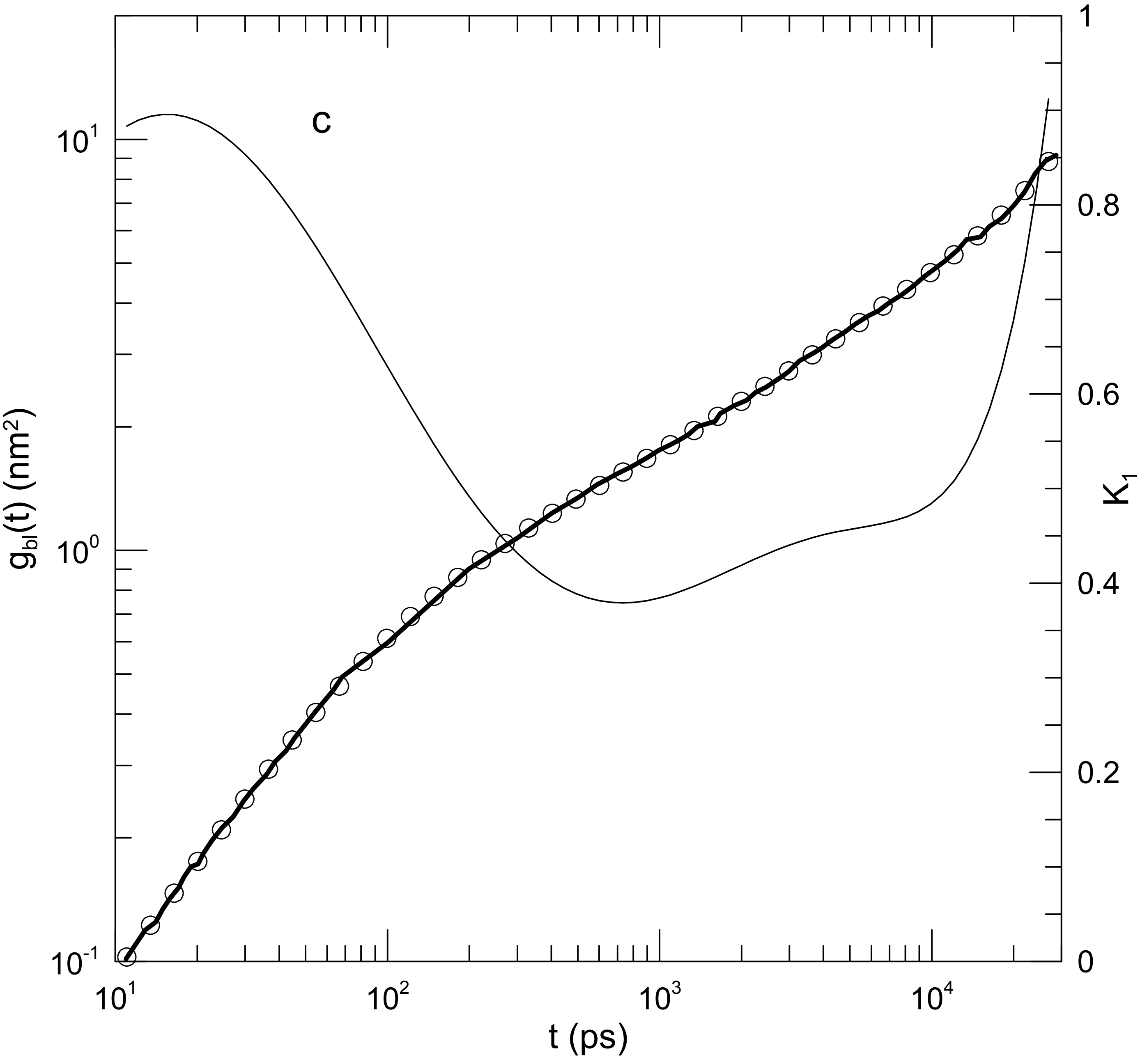

[1] Behbahani, A.F.; Schmid, F. Relaxation Dynamics of Entangled Linear Polymer Melts via Molecular Dynamics Simulations. *Macromolecules* **2025**, *58*, 767–786.

[2] R. Chang, R.; A. Yethiraj, A. Can Polymer Chains Cross Each Other and Still Be Entangled? *Macromolecules* **2019**, *52*, 2000–2006.

[3] Tsalikis, D.G.; Koukoulas, T.; Mavrantzas, V.G.; Pasquino, R.; Vlassopoulos, D.; Pyckhout-Hintzen, W.; Waschnewski, A.; Monkenbusch, M.; D. Richter, D. Microscopic Structure, Conformation, and Dynamics of Ring and Linear Poly(ethylene oxide) Melts from Detailed Atomistic Molecular Dynamics Simulation: Dependence on Chain Lengths and Direct Comparison with Experimental Data. *Macromolecules* **2017**, *50*, 2565–2584.

[4] Takahashi, K.Z.; Nishimura, R.; Yasuoka, K.; Masabuchi, Y. Molecular Dynamics Simulations for Resolving Scaling Laws of Polyethylene Melts. *Polymers* **2017**, *9*, 24.

[5] Peng, W.; Ranganathan, R.; Keblinski, P.; Ozisik, R. Viscoelastic and Dynamic Properties of Well-Mixed and Phase-Separated Binary Polymer Blends: A Molecular Dynamics Simulation Study. *Macromolecules* **2017**, *50*, 6293–6302.

[6] Hsu, H.-P.; Kremer, K. Static and Dynamic Properties of Large Polymer Melts in Equilibrium. *J. Chem. Phys.* **2016**, *144*, 154907.

[7] Brodeck, M.; Alvarez, F.; Moreno, A.J.; Colmenero, J.; Richter, D. Chain Motion in Nonentangled Dynamically Asymmetric Polymer Blends: Comparison between Atomistic Simulations of PEO/PMMA and a Generic Bead-Spring Model. *Macromolecules* **2010**, *43*, 3036–3051.

[8] Stephanou, P.S.; Baig, C.; Tsolou, G.; Mavrantzas, V.G.; Kroeger, M. Quantifying Chain Reptation in Entangled Polymer Melts: Topological and Dynamical Mapping of Atomistic Simulation Results onto the Tube Model. *J. Chem. Phys.* **2010**, *132*, 124904.

[9] Likhtman, A.E.; Sukumaran, S.K.; Ramirez, J. Linear Viscoelasticity from Molecular Dynamics Simulation of Entangled Polymers. *Macromolecules* **2007**, *40*, 6748–6757/

[10] Zhou, Q.;Larson, R.G. Direct Calculation of the Tube Potential Confining Entangled Polymers. *Macromolecules* **2006**, *39*, 6737–6743.

[11] Moreno, A.J.; Colmenero, J. Is There a Higher-Order Mode-Coupling Transition in Polymer Blends. *J. Chem. Phys.* **2006**, *124*, 184906.

[12] Padding, J.T.; Briels, W.J. Zero Shear Stress Relaxation and Long Time Dynamics of a Linear

Polethylene Melt: A Test of Rouse Theory. *J. Chem. Phys.* **2001**, *114*, 8685–8693.

[13] Padding, J.T.; Briels, W.J. Time and Length Scales of Polymer Melts Studied by Coarse-Grained Molecular Dynamics Simulations. *J. Chem. Phys.* **2002**, *117*, 925–943.
